# Supplementary material for: A MICA/B GAALIE-mutant antibody elicits potent natural killer cell-driven immunity in solid and hematologic malignancy models
Source: Cell Rep Med. 2026 Apr 17;7(5):102753. doi: 10.1016/j.xcrm.2026.102753 (PMC13198289; doi:10.1016/j.xcrm.2026.102753)
Supplement: Document S1. Figures S1–S17 [file mmc1.pdf]

Cell Reports Medicine, Volume *7*

Supplemental information

A MICA/B GAALIE-mutant antibody elicits potent natural killer cell-driven immunity in solid and hematologic malignancy models

Ruan Pimenta, Stefanie Maurer, Xiaoxuan Zhong, Bruna Taciane da Silva Bortoleti, Sophia Quasem, Luiza Ribeiro de Lima Brandão, Bridget Marcellino, Anindita Dutta, Juan M. Arriaga, John Mascarenhas, and Lucas Ferrari de Andrade

Supplementary figure 1

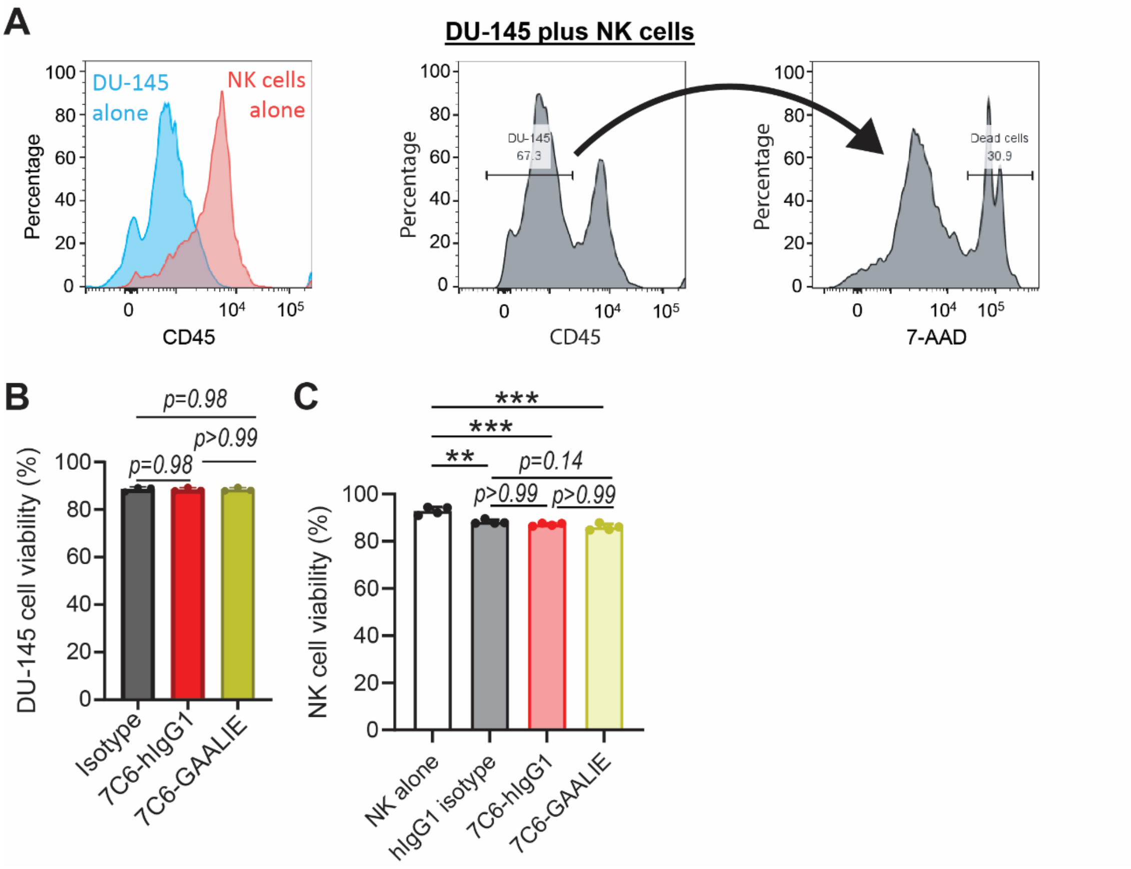

Supplementary figure 1. Representation of the flow cytometry-based NK cell cytotoxicity assay and analysis of tumor cell and NK cell viability. (A-C) DU-145 and NK cells were co-cultured for four hours, which was followed by labelling with anti-CD45 and 7-AAD. DU-145 alone and NK cells alone were used as controls. **(A)** Illustration of the gating strategy for analyzing DU-145 cell death. **(B)** Analysis of tumor cell viability in the absence of NK cells. **(C)** Analysis of NK cell viability after co-culture with tumor cells. Data are mean ± standard deviation (SD) of triplicates (B) or quadruplicates (C), represent three independent experiments with two NK cell donors (A, C), and were analyzed by one-away analysis of variance (ANOVA) with Bonferroni's test (B-C). **p<0.01, ***p<0.001. Related to Figure 1F-G.

Supplementary figure 2

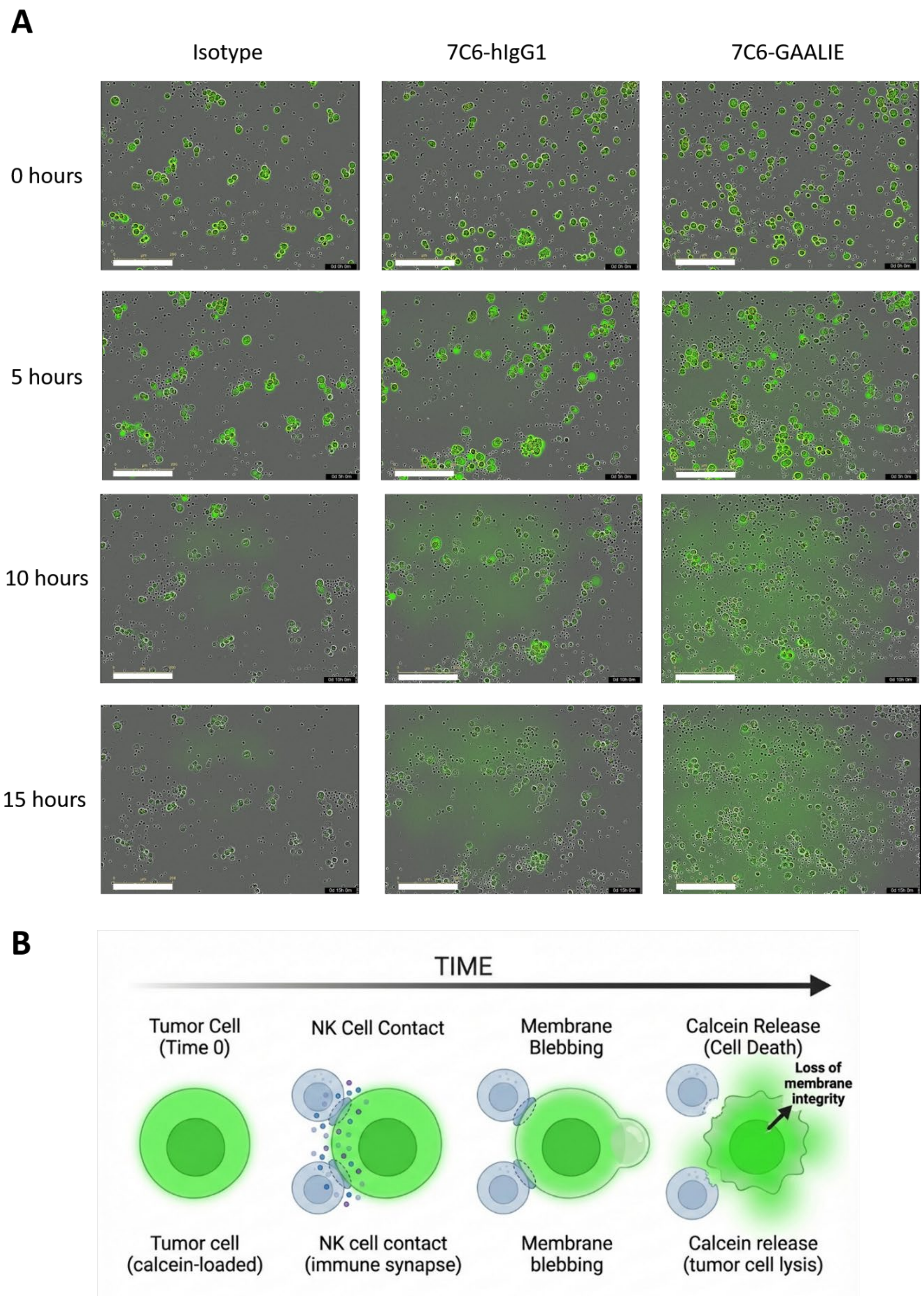

Supplementary figure 2. Analysis of human NK cell + DU-145 co-culture by IncuCyte. (A) NK cells were co-cultured with DU145 cells that were treated with the indicated antibodies and labeled with calcein (green color), and images were recorded by Incucyte at the indicated times. Data represent two independent experiments with two different NK cell donors. The scale bars represent 200 µm. **(B)** Illustration of the observed interaction between NK cells and tumor cells, whereby in the beginning of the assay all tumor cells are labeled with calcein but are then recognized and killed by NK cells, resulting in calcein release in the supernatant. Related to STAR Methods "Incucyte live-cell imaging assays."

Supplementary figure 3

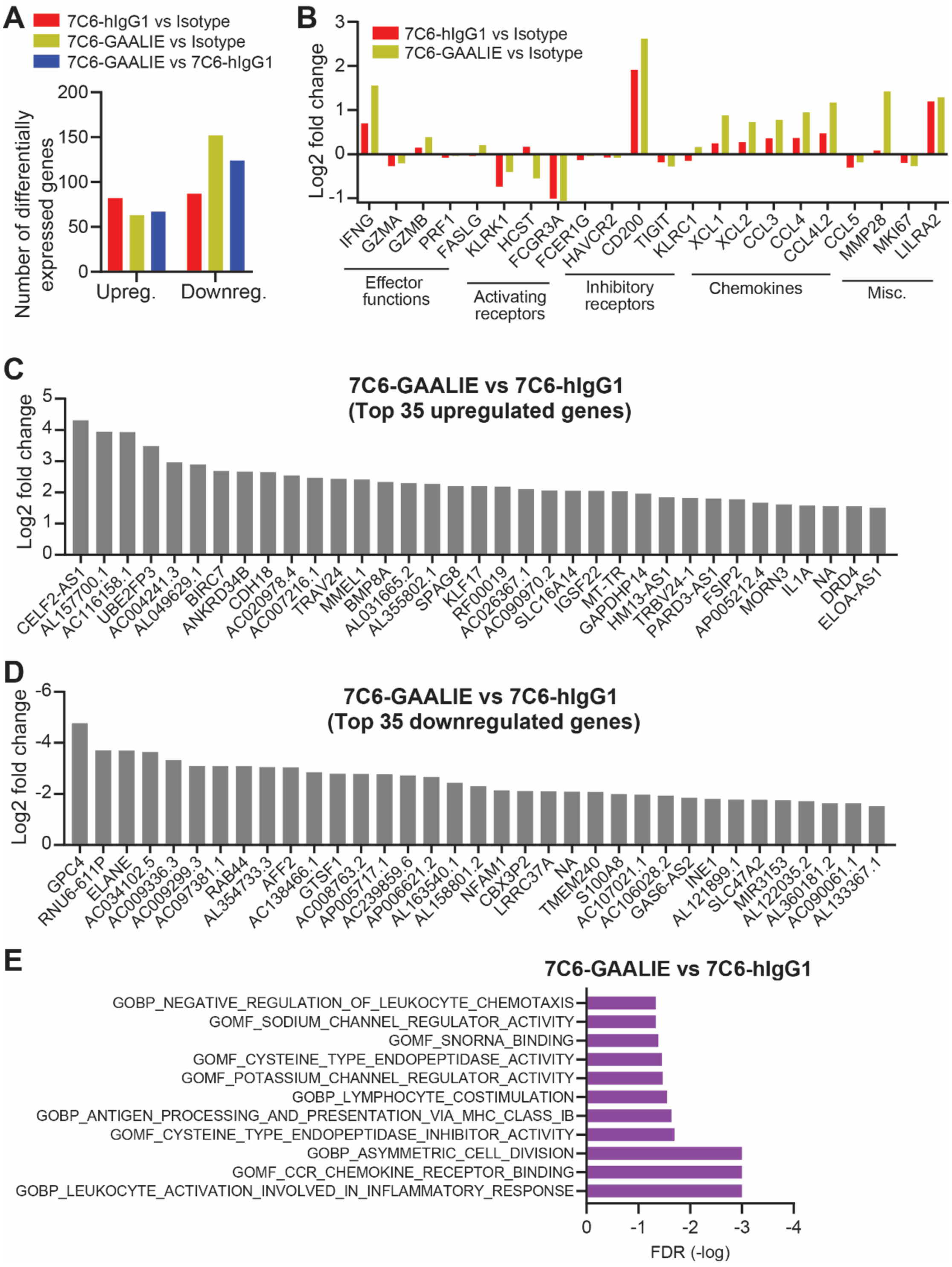

Supplementary figure 3. RNA-seq analysis of human NK cells that were co-cultured with DU-145. DU-145 cells were treated for 24-hours with hIgG1 isotype, 7C6-hIgG1, or 7C6-GAALIE, which was followed by co-culture with primary NK cells from one donor, which were then isolated and analyzed by RNA-seq. **(A)** Summary of differentially expressed genes, with 5 % significance and log2 fold change ≥ 1. Upreg. = upregulated; Downreg. = downregulated. **(B)** Gene expression analysis of some key genes related to NK cell-driven immunity. **(C - D)** The top 35 differentially expressed genes in NK cells, after the comparison of 7C6-GAALIE against 7C6-hIgG1, with 5 % significance and log2 fold change ≥ 1. **(E)** Gene set enrichment analysis of NK cells, comparing 7C6-GAALIE against 7C6-hIgG1. Related to STAR Methods "RNA-sequencing of human NK cells."

Supplementary figure 4

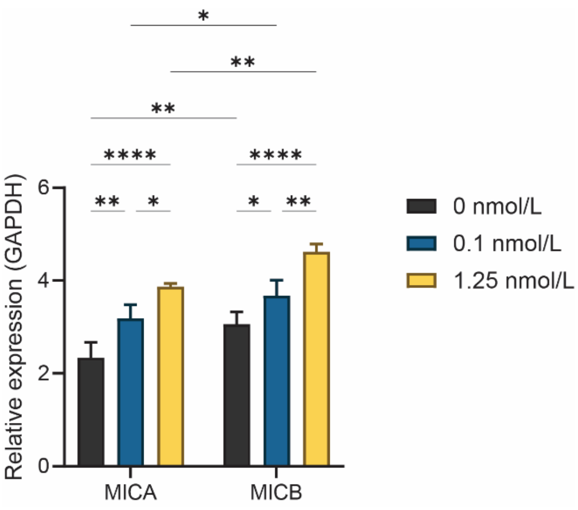

Supplementary figure 4. DU-145 cells were treated for one day with the indicated concentrations of romidepsin, and MICA/B gene expression was analyzed by quantitative polymerase chain reaction with housekeeping gene normalization. Data represent three independent experiments, are mean ± SD of triplicates, and were analyzed by two-way ANOVA with Bonferroni's test. *p<0.05, **p<0.01, ***p<0.001. Related to STAR Methods "MICA and MICB gene expression analysis."

Supplementary figure 5

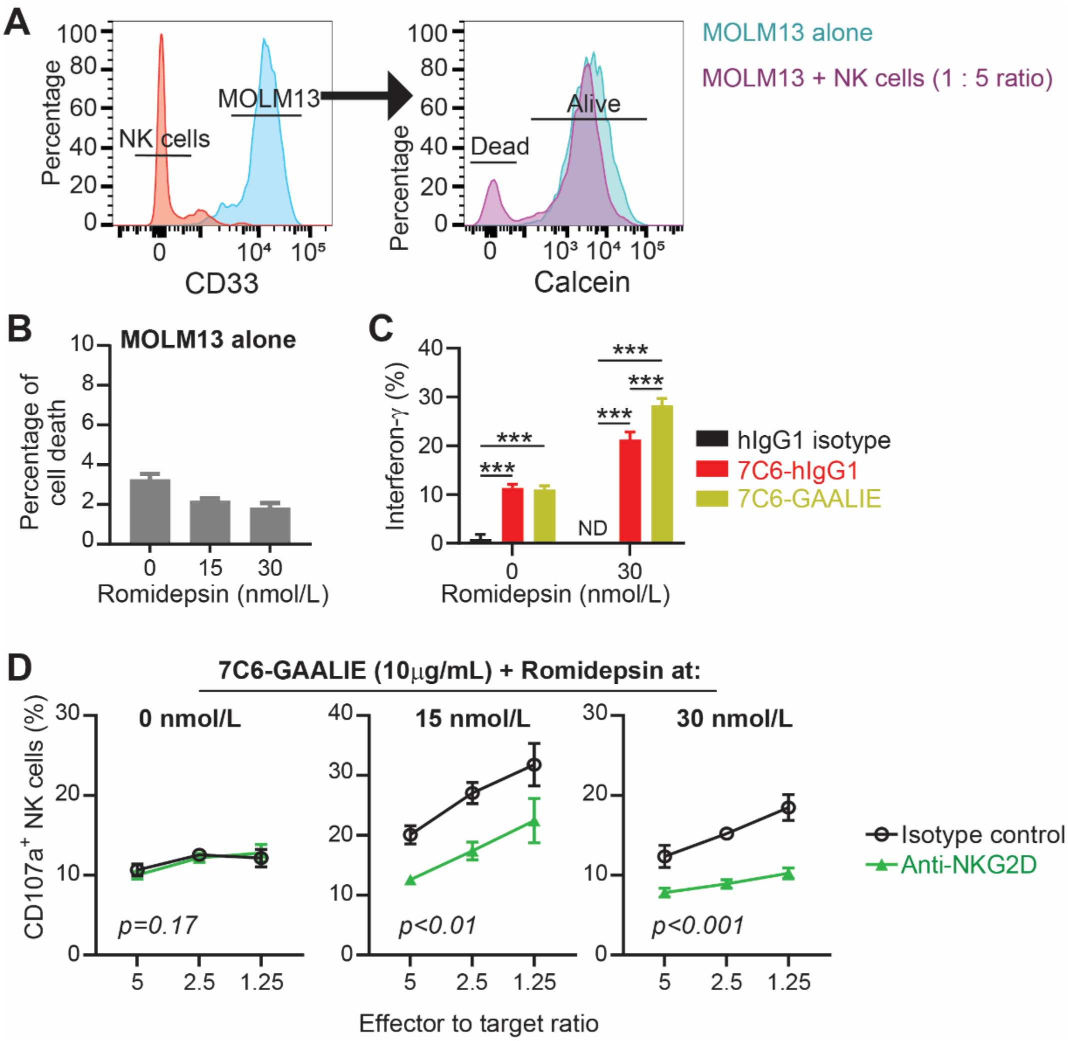

Supplementary figure 5. NK cell effector function assays with MOLM13 as the target cells. (A) Histograms of flow cytometry-based calcein assay, illustrating the identification of dead and alive MOLM13 cells after the co-culture with NK cells. **(B)** Romidepsin by itself did not kill MOLM13 cells. Quantification of dead MOLM13 cells by calcein release and flow cytometry. **(C)** 7C6-GAALIE increases the NK cell interferon-γ production. NK and MOLM13 cells were co-cultured for six hours in the presence of brefeldin A, followed by analysis of intracellular interferon-γ through flow cytometry. ND = not detected. In these experiments, MOLM13 were pre-treated with the indicated doses of romidepsin and mAbs for 24 hours. **(D)** MOLM13 was pre-treated for 24 hours with 7C6-GAALIE and the indicated doses of romidepsin. NK cells were co-cultured with the pre-treated MOLM13 cells for four hours in the presence of anti-CD107a antibody, with the addition of monensin after the first hour. Analyses by flow cytometry. Data represent three independent experiments (B-D) with three (A) or two NK cell donors (C-D), are mean ± SD (B-C) or standard error (SE) (D) of triplicates (B-D), and were analyzed by two-way ANOVA (C-D) with Bonferroni's test (C). ***p<0.001. Related to Figure 2.

Supplementary figure 6

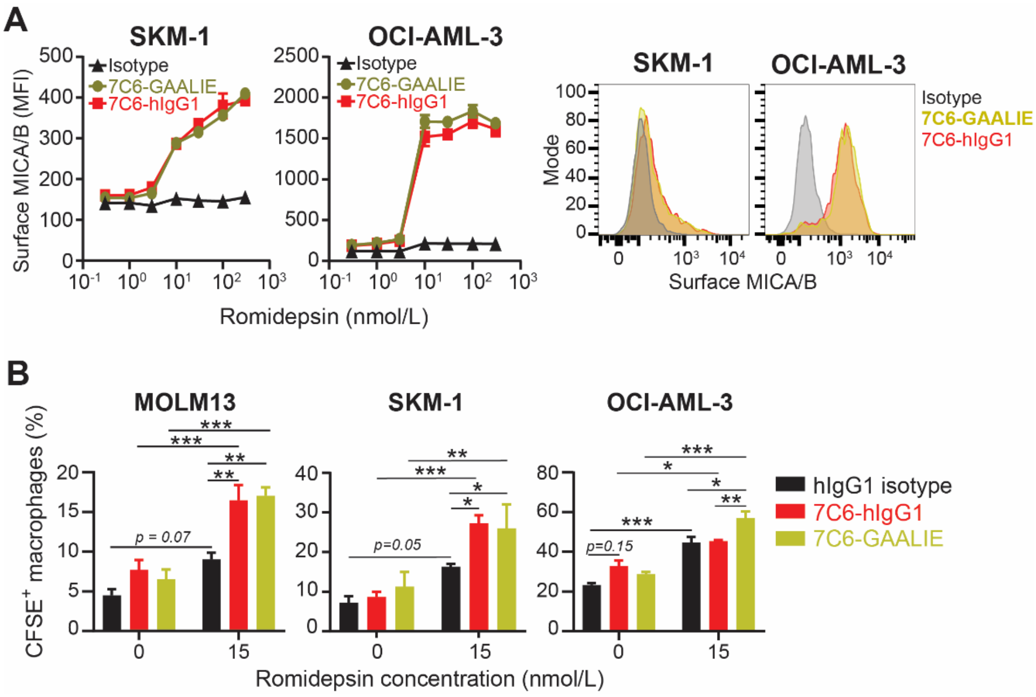

Supplementary figure 6. MICA/B surface expression upregulation in two human AML cell lines and ADCP assays. (A) SKM-1 and OCL-AML-3 cells were treated for 24 hours with the indicated antibodies and romidepsin doses, and MICA/B surface expression was analyzed by flow cytometry. The histograms represent MICA/B surface expression at romidepsin dose of 30 nmol/L. **(B)** 7C6-GAALIE and 7C6-hIgG1 trigger ADCP against romidepsin-treated MOLM13 and SKM-1, and only the first triggered ADCP against OCI-AML-3. The indicated leukemia cell lines were pre-treated for 24 hours with the indicated antibodies and romidepsin doses, labelled with CFSE, and used as target cells in 2-hours phagocytosis assays with human monocyte-derived macrophages. Analyses were by flow cytometry with macrophage identification by CD206 expression. Data represent three independent experiments (A-B) with at least two monocyte donors (B), are mean ± SD (A) or SE (B) of triplicates (A-B), and were analyzed by two-way ANOVA with Bonferroni's test (B). *p<0.05, **p<0.01, ***p<0.001. Related to Figure 2 and STAR Methods "Phagocytosis assays."

Supplementary figure 7

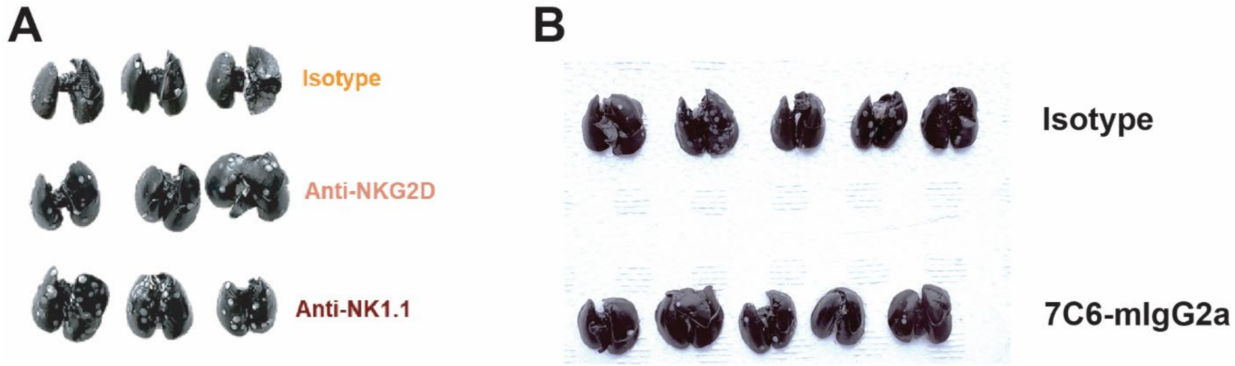

Supplementary figure 7. Representative images of lungs in the mouse prostate cancer metastasis model. (A-B) Representative images of the data that are presented in Figure 3D (A) and Figure 3G (B). Related to Figures 3D and 3G.

Supplementary figure 8

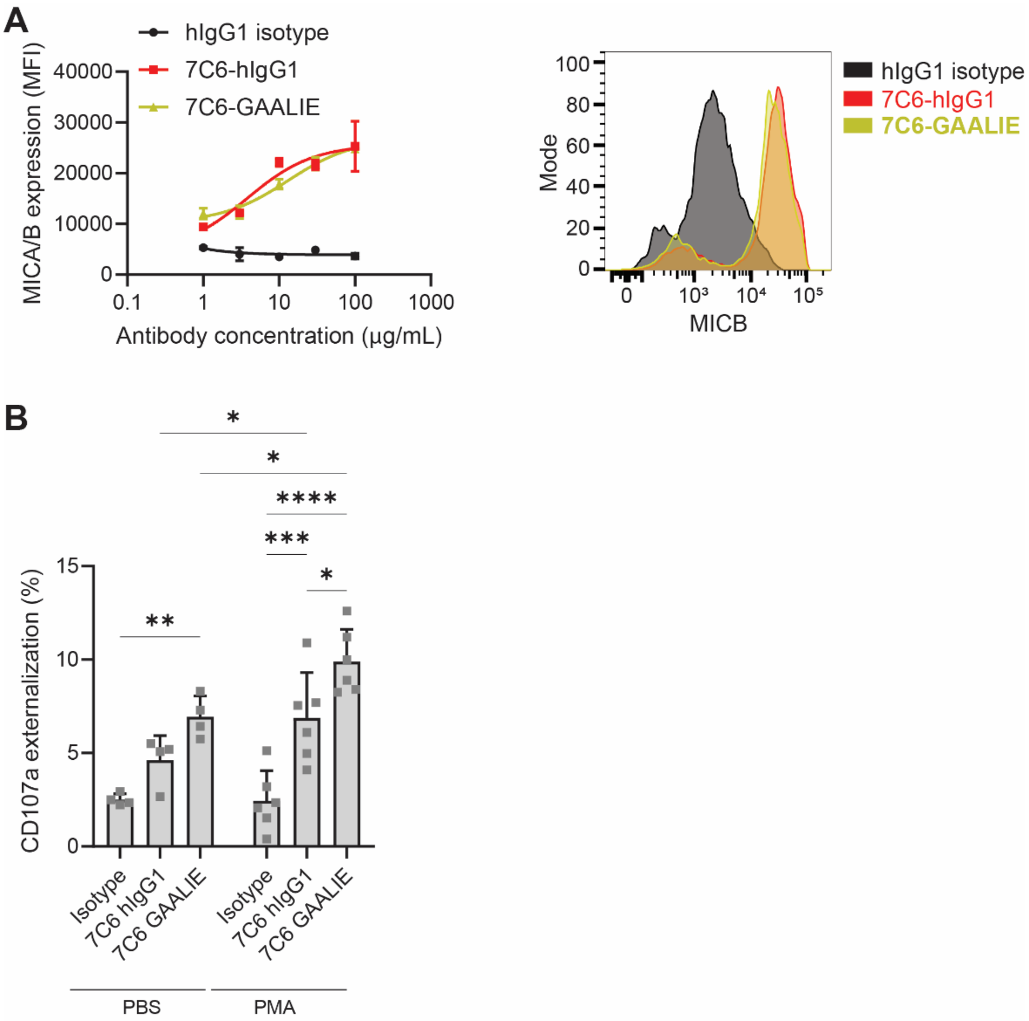

Supplementary figure 8. *In vitro* and *ex vivo* analyses of MICA/B shedding and NK cell degranulation, respectively. (A) NPK-MICB cells were treated for 24 hours with the indicated concentrations of the indicated antibodies, and MICB surface expression was analyzed by flow cytometry. The histograms represent the MICB surface expression levels at the antibody highest dose, 100 g/mL. **(B)** hFcR mice were subjected to the mouse prostate cancer metastasis model and antibody treatments as in Figure 4C. On day 14, lung NK cells were analyzed for degranulation *in vitro* after stimulation with PMA or treatment with PBS, a control. Data represent three (A) or are pooled of two (B) independent experiments, are mean ± SD (A-B), and were analyzed by non-linear regression (A) or two-way ANOVA with Bonferroni's test (B). *p<0.05, **p<0.01, ***p<0.001. Related to STAR Methods "MICA/B shedding assays with cell lines"and "Mouse prostate cancer metastasis model."

Supplementary figure 9

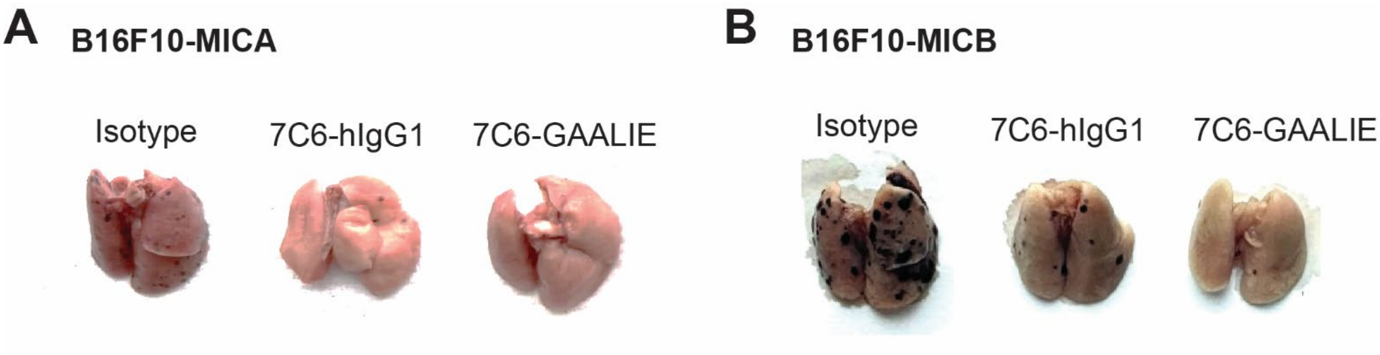

Supplementary figure 9. Representative lung images of hFcR mice that underwent the B16F10-MICA or B16F10-MICB metastasis models and treatment with antibodies. (A-B) Representative lung images of the data shown in Figure 5B. Related to Figure 5A-B.

Supplementary figure 10

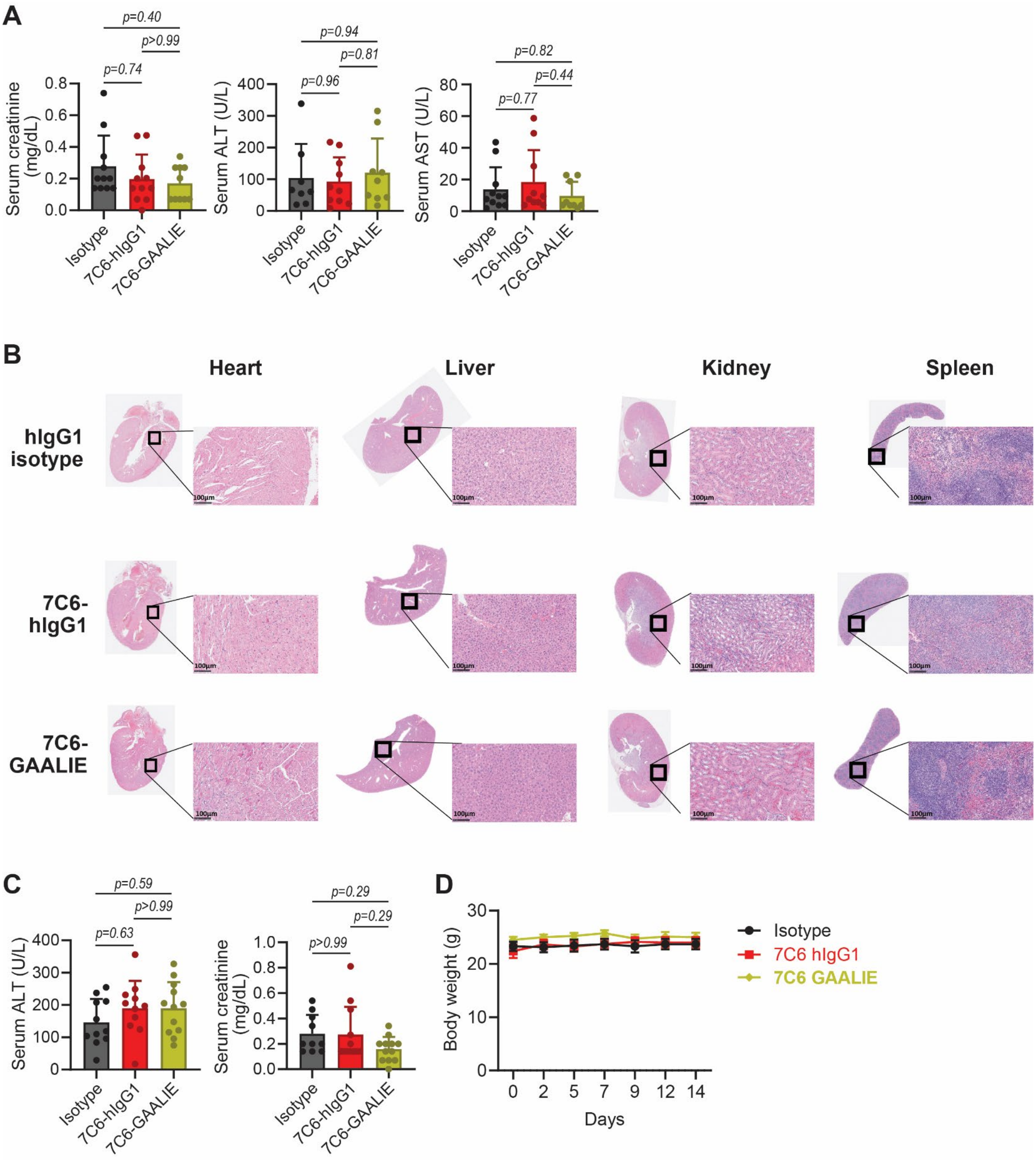

Supplementary figure 10. Toxicology of 7C6-hIgG1 and 7C6-GAALIE in mouse metastasis models. (A-B) Analysis of toxicology by serum biomarkers (A) and histopathology (B) of hFcR mice that were subjected to the mouse prostate cancer metastasis model and euthanized when reached the humane endpoint; these hFcR mice are the same of the survival experiment that is shown in Figure 4F. The scale bars represent 100 µm (B). **(C-D)** Analysis of toxicology by serum biomarkers (C) and longitudinal weight measurements (D) of hFcR mice that were subjected to the B16F10-MICA metastasis model; these analyses were performed in the same mice under the experiments that are shown in Figure 5B, B16F10-MICA. In D: Isotype hIgG1 n = 7, 7C6-hIgG1 n = 8, and 7C6-GAALIE n = 8. Data are pooled of two independent experiments (A, C-D), represent fifteen mice per antibody group (B), are mean ±

SD (A, C) or SE (D), and were analyzed by two-tailed unpaired Student's t tests with the indicated comparisons (A, C). Related to Figures 4 and 5.

Supplementary figure 11

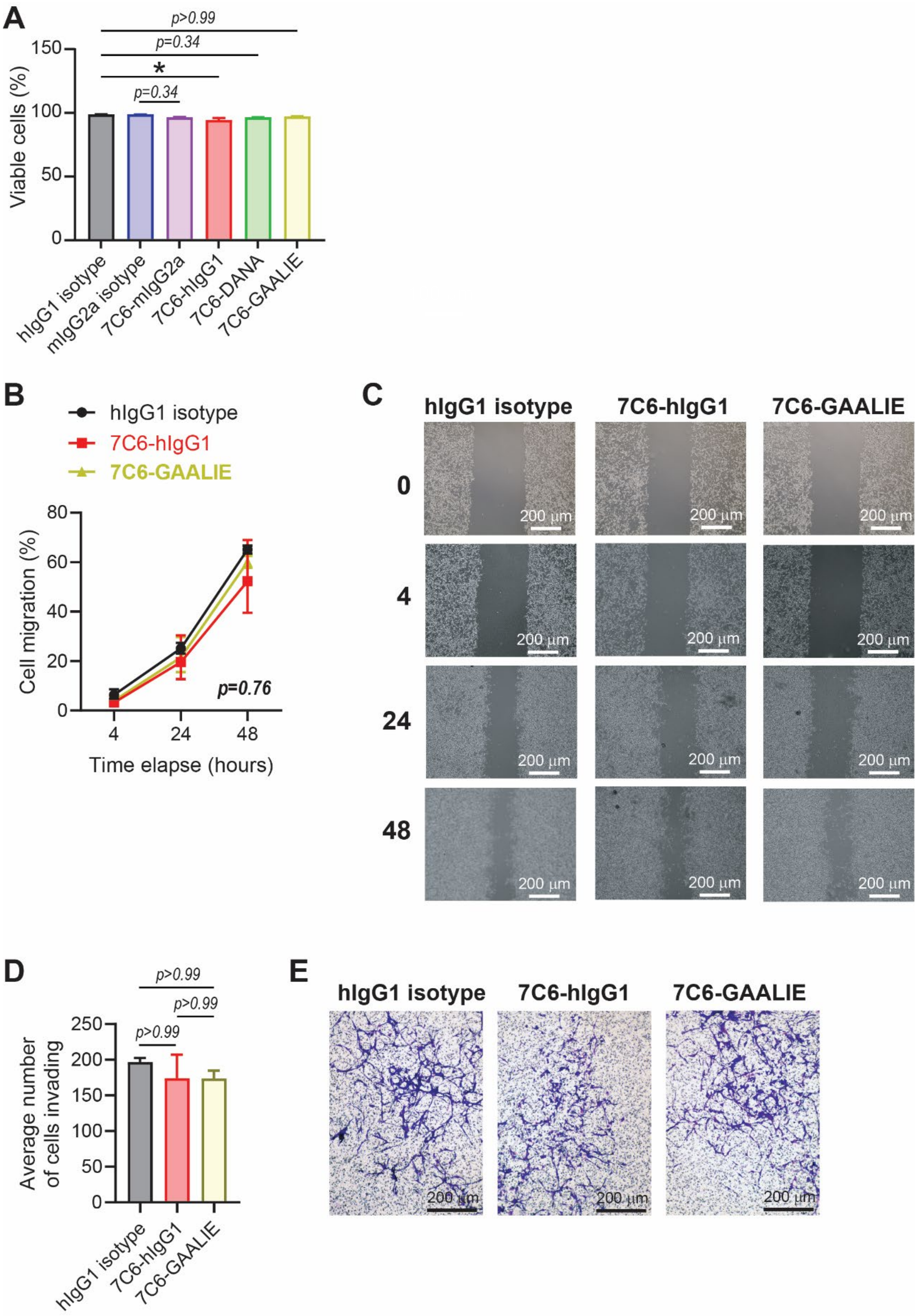

Supplementary figure 11. NPK-MICB viability, migration, and invasion analyses. (A) Analysis of NPK-MICB viability. The cells were treated for 24 hours with the indicated antibodies, and cell viability was analyzed by flow cytometry. **(B-C)** NPK-MICB migration assay. NPK-MICB cells were treated with the indicate antibodies and, at the same time, a scratch was done in the tissue culture plate, and illustrated in 'C'. Analyses of cell migration were done by microscopy on the indicated time points. **(D-E)** Analysis of NPK-MICB invasion in the transwell system. The cells were cultured for 48 hours in a transwell that was coated with matrigel, and the cells that invaded the lower surface were counted by microscopy. Data are mean ± SD (A, D) or SE (B) of duplicates (A, D) or triplicates (B), were analyzed by one-way (A, D) or two-way (B) ANOVA with Bonferroni's test (D), and represent three (A-B) or

two (C) independent experiments. *$p<0.05$. The scale bars represent 200 μm (C, E). Related to STAR Methods “Wound healing (scratch) assay” and “Transwell invasion assay.”

Supplementary figure 12

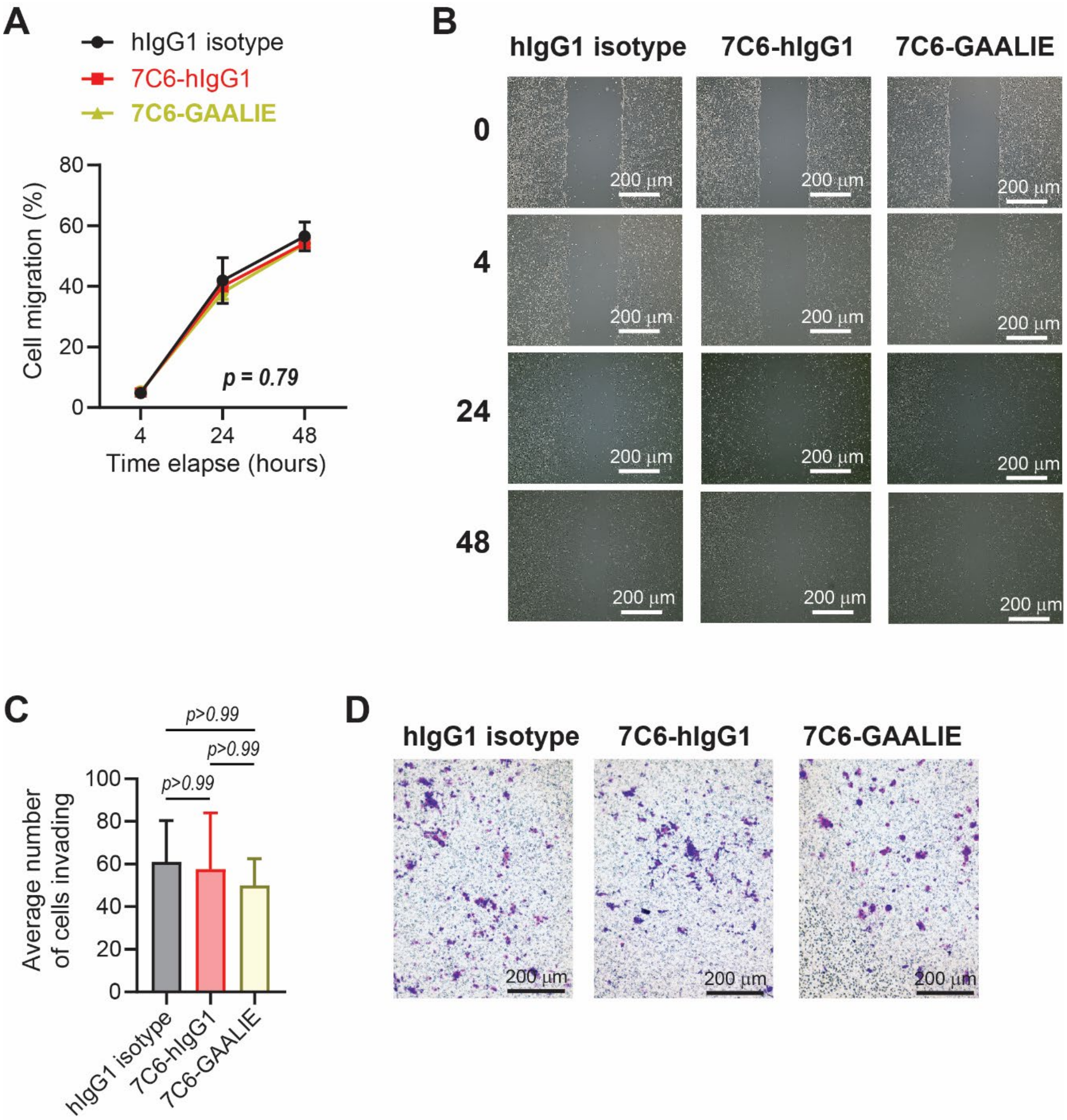

Supplementary figure 12. Migration and invasion assays with DU-145 cell line. (A-B) DU-145 cells were incubated with the indicated antibodies and a scratch was done on the tissue culture plates, as indicated in 'B'. Cell migration was analyzed by microscopy in the indicated hours. **(C-D)** DU-145 cells were cultured in transwell plates and treated with the indicated antibodies. After 48 hours, cell invasion to the lower surface was analyzed by microscopy. Data are mean ± SD (A, C) of triplicates (A, C), represent three (B) or two (D) independent experiments, and were analyzed by one-way (C) or two-way (A) ANOVA with Bonferroni's test (C). The scale bars represent 200 µm (B, D). Related to STAR Methods "Wound healing (scratch) assay" and "Transwell invasion assay."

Supplementary figure 13

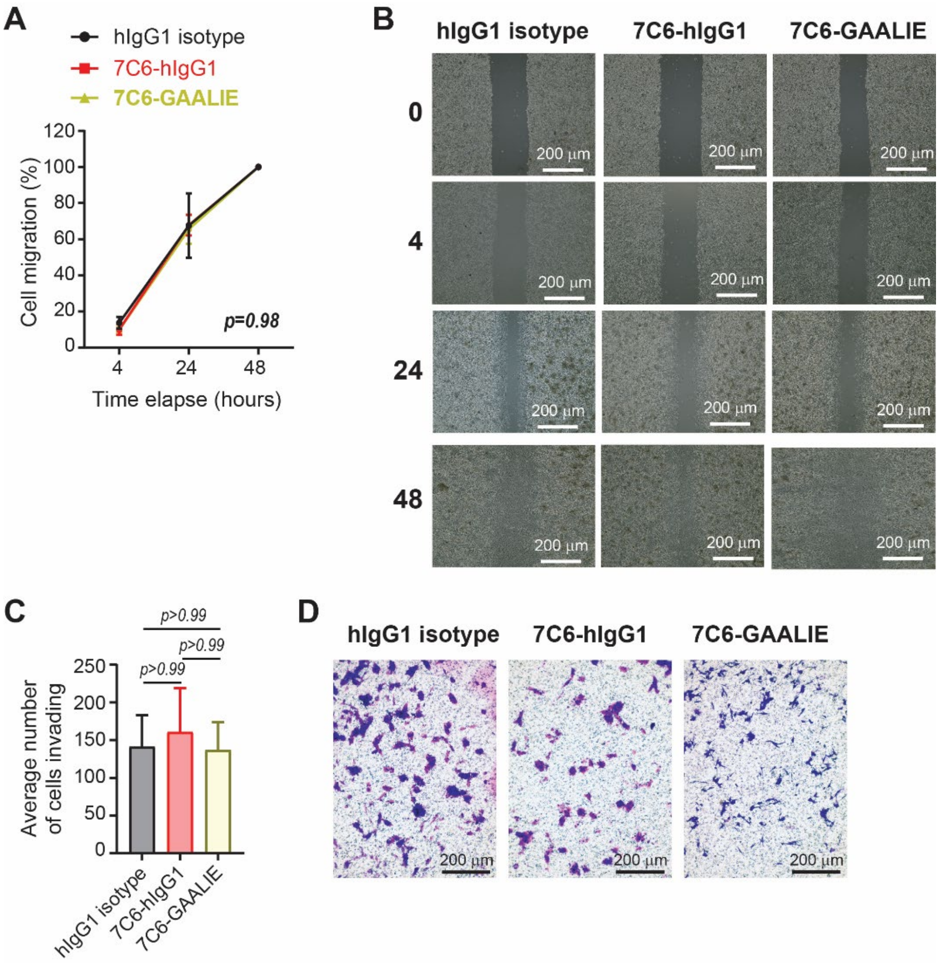

Supplementary figure 13. Migration and invasion assays with B16F10-MICA cell line. (A-B) B16F10-MICA cells were incubated with the indicated antibodies and a scratch was done on the tissue culture plates, as indicated in 'B'. Cell migration was analyzed by microscopy in the indicated hours. **(C-D)** B16F10-MICA cells were cultured in transwell plates and treated with the indicated antibodies. After 48 hours, cell invasion to the lower surface was analyzed by microscopy. Data are mean ± SD (A, C) of triplicates (A, C), represent three (A) or two (C) independent experiments, and were analyzed by one-way (C) or two-way (A) ANOVA with Bonferroni's test (C). The scale bars represent 200 µm (B, D). Related to STAR Methods "Wound healing (scratch) assay" and "Transwell invasion assay."

Supplementary figure 14

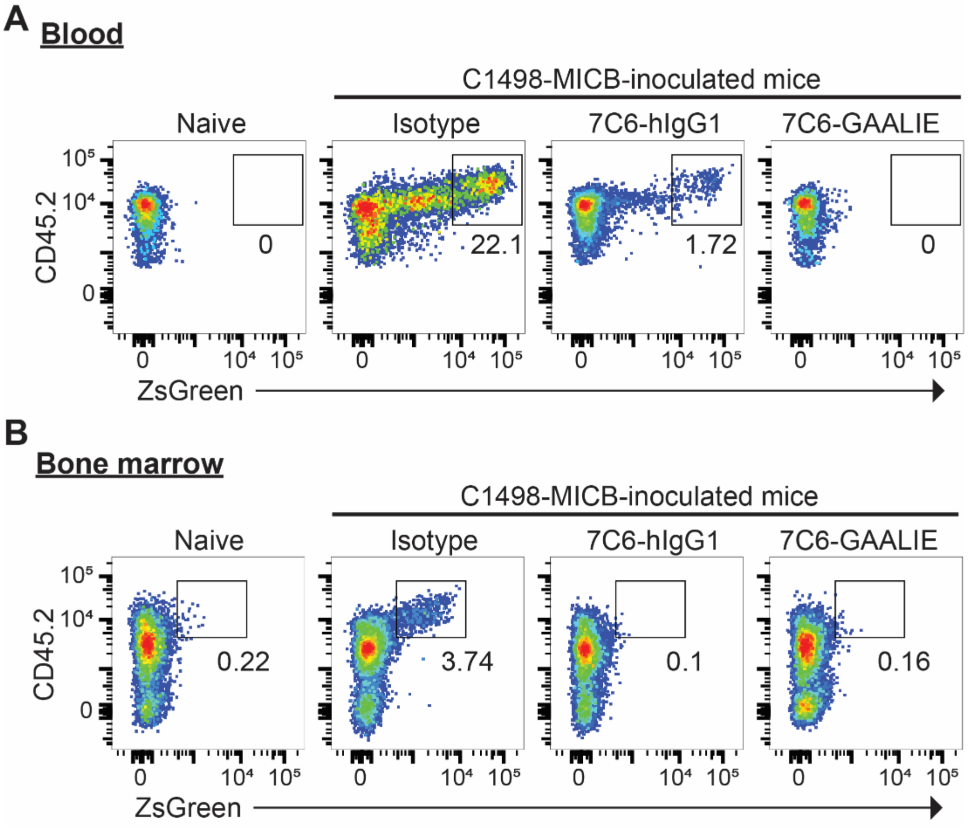

Supplementary figure 14. ZsGreen-based identification of C1498-MICB in the blood and bone marrow of hFcR mice. (A-B) Flow cytometry plots representing the data shown in Figure 5D. Related to Figure 5D.

Supplementary figure 15

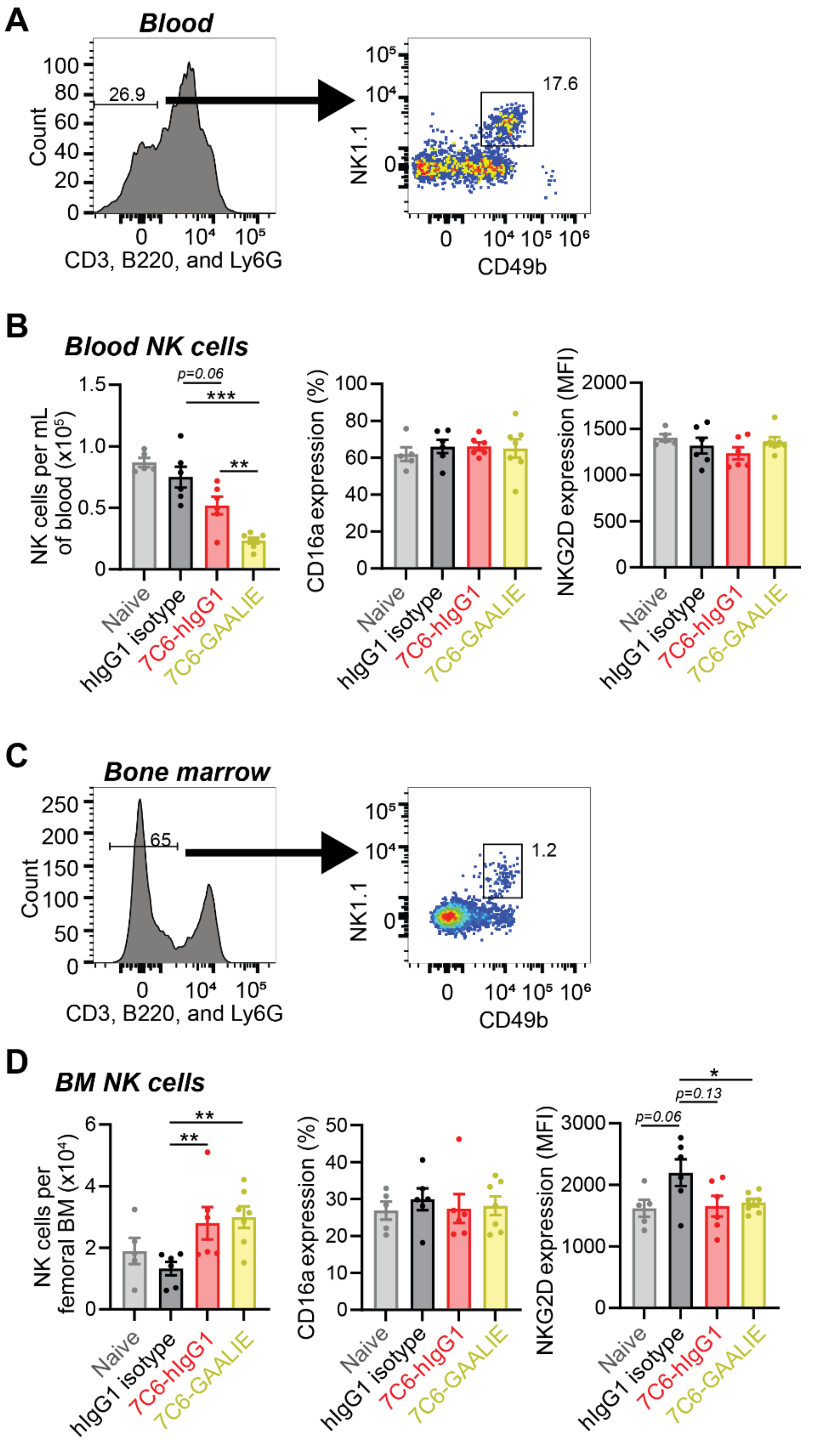

Supplementary figure 15. Characterization of NK cells in the mouse AML model in hFcR mice. (A-B) Blood NK cell analyses. **(A, C)** Gating strategies for identification of blood (A) or bone marrow (C) NK cells by flow cytometry. **(C-D)** Bone marrow (BM) NK cell analyses. Data represent two independent experiments (B, D), are mean ± SE (B, D), and were analyzed by two-tailed Mann-Whitney tests (B, D). Each dot represents one mouse (B, D). *p<0.05, **p<0.01, ***p<0.001. Related to Figure 5C-D.

Supplementary figure 16

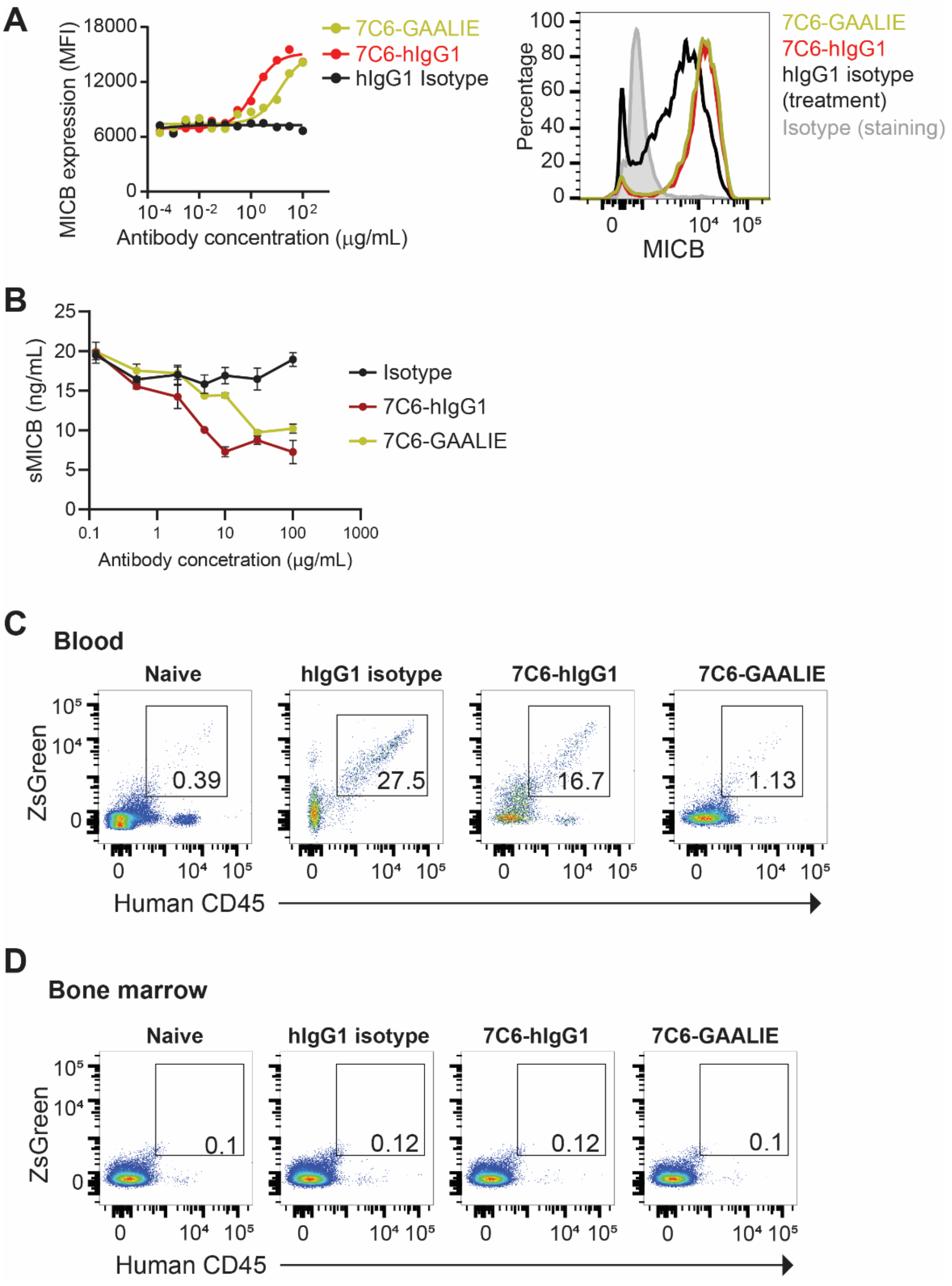

Supplementary figure 16. Validation of MOLM13-MICB and identification of MOLM13-MICB in the blood and bone marrow of FcResolv® hIL-15 NOG mice that were reconstituted with human NK cells. (A-B) MOLM13-MICB was treated for one day with the indicated concentrations of the indicated antibodies and surface MICB (A) and soluble MICB (sMICB) shed in supernatants (B) were analyzed by flow cytometry or ELISA, respectively. Data represent three independent experiments (A-B), are mean ± SD of triplicates (B) or one observation data point (A), and were analyzed by non-linear regression (A). The histograms show MICB surface expression after antibody treatment at the highest dose. **(C-D)** Flow cytometry plots representing the data of blood leukemia cells shown in Figure 5F. Mouse CD45.1^{+} cells were gated out (not shown) prior to calculation of absolute numbers. Related to Figure 5E-F.

Supplementary figure 17

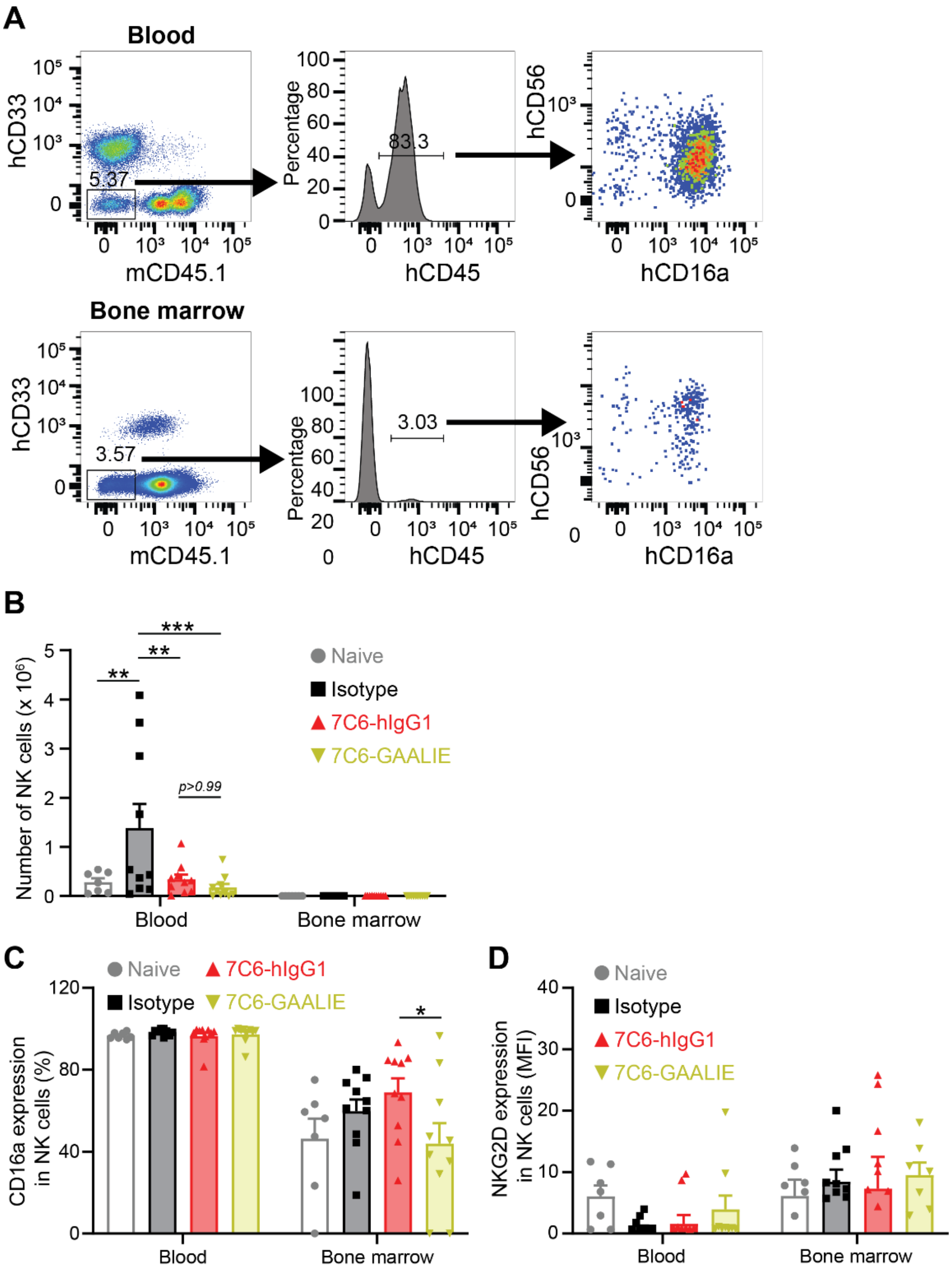

Supplementary figure 17. Characterization of human NK cells in the MOLM13-MICB model with FcResolv® hIL-15 NOG mice. (A-D) The mice were subjected to the NK cell reconstitution and MOLM13-MICB inoculation as illustrated in Figure 3E. **(A)** Flow cytometry analyses that enabled the identification of leukemia and NK cells in the blood and bone marrow. **(B)** Absolute numbers of NK cells in the blood and bone marrow. **(C)** Percentage of CD16a expression in NK cells. **(D)** NKG2D expression analyses, after normalization with the isotype control for the staining. MFI = mean fluorescence intensity. Data are mean ± SE (B-D), are pooled of two independent experiments (B-D), and were analyzed by two-way ANOVA with Bonferroni's test (B-D). Each dot represents one mouse (B-D). *$p<0.05$, **$p<0.01$, ***$p<0.001$. Related to Figure 5E-F.
