# Supplementary material for: A MICA/B GAALIE-mutant antibody elicits potent natural killer cell-driven immunity in solid and hematologic malignancy models
Source: Cell Rep Med. 2026 Apr 17;7(5):102753. doi: 10.1016/j.xcrm.2026.102753 (PMC13198289; doi:10.1016/j.xcrm.2026.102753)
Supplement: Document S2. Article plus supplemental information [file mmc2.pdf]

Cell Reports
Medicine

Article

A MICA/B GAALIE-mutant antibody elicits potent natural killer cell-driven immunity in solid and hematologic malignancy models

Graphical abstract

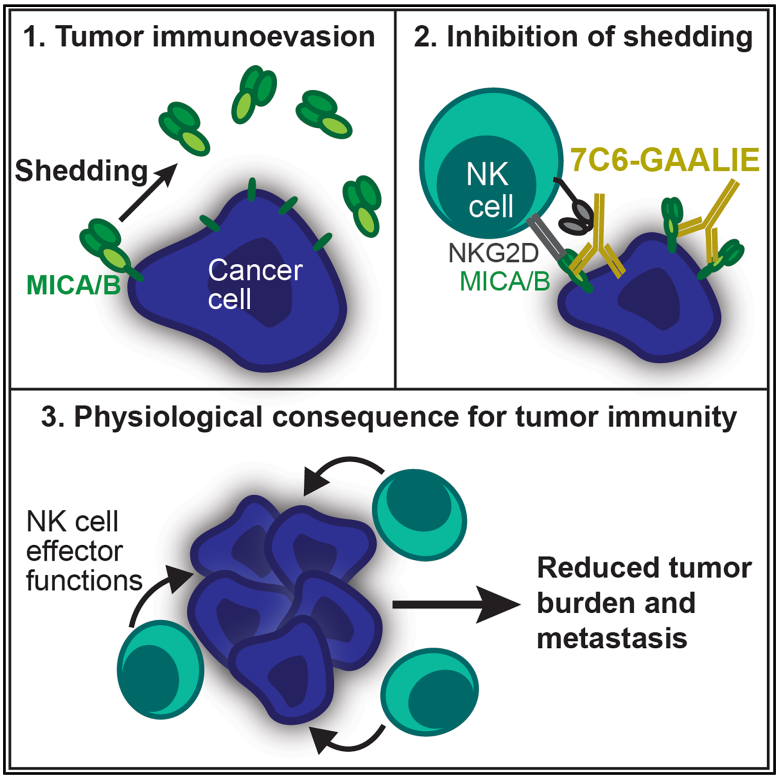

Highlights

- GAALIE mutations increase the binding affinity of 7C6 to Fc gamma-activating receptors
- 7C6-GAALIE inhibits MICA/B shedding similarly to Fc-wild-type 7C6
- 7C6-GAALIE more potently promotes ADCC by NK cells against cancer cells
- 7C6-GAALIE potently inhibits the *in vivo* development of metastases and leukemia

Authors

Ruan Pimenta, Stefanie Maurer, Xiaoxuan Zhong, ..., Juan M. Arriaga, John Mascarenhas, Lucas Ferrari de Andrade

Correspondence

lucas.ferrarideandrade@mssm.edu

In brief

Pimenta et al. engineer the Fc of an antibody (7C6), which inhibits MICA and MICB shedding, by GAALIE mutations that increase the binding affinity to Fc gamma-activating receptors. 7C6-GAALIE potently promotes antibody-dependent cellular cytotoxicity and inhibits cancer development in models of prostate cancer and melanoma metastases and acute myeloid leukemia.

Pimenta et al., 2026, Cell Reports Medicine 7, 102753
May 19, 2026 © 2026 The Author(s). Published by Elsevier Inc.
https://doi.org/10.1016/j.xcrm.2026.102753

CellPress

Cell Reports Medicine

CellPress
OPEN ACCESS

Article

A MICA/B GAALIE-mutant antibody elicits potent natural killer cell-driven immunity in solid and hematologic malignancy models

Ruan Pimenta,[1,5] Stefanie Maurer,[1,5] Xiaoxuan Zhong,[1] Bruna Taciane da Silva Bortoleti,[1] Sophia Quasem,[1] Luiza Ribeiro de Lima Brandão,[1] Bridget Marcellino,[2] Anindita Dutta,[3,4] Juan M. Arriaga,[3,4] John Mascarenhas,[2] and Lucas Ferrari de Andrade[1,3,6,*]

[1]Department of Immunology and Immunotherapy, and Marc and Jennifer Lipschultz Precision Immunology Institute, Icahn School of Medicine at Mount Sinai, New York, NY 10029, USA
[2]Division of Hematology-Oncology, Department of Medicine, Icahn School of Medicine at Mount Sinai, New York, NY 10029, USA
[3]Department of Oncological Sciences and Tisch Cancer Institute, Icahn School of Medicine at Mount Sinai, New York, NY 10029, USA
[4]Department of Urology, Icahn School of Medicine at Mount Sinai, New York, NY 10029, USA
[5]These authors contributed equally
[6]Lead contact
*Correspondence: lucas.ferrarideandrade@mssm.edu
https://doi.org/10.1016/j.xcrm.2026.102753

SUMMARY

Anti-MICA/B antibodies that inhibit the shedding hold promise for cancer immunotherapy and are in phase 1 clinical trials. Although MICA/B-shedding inhibition promotes natural killer (NK) cell-driven immunity by NKG2D engagement, the Fc enables antibody-dependent cellular cytotoxicity. We, therefore, engineer the Fc of an anti-MICA/B antibody (clone 7C6) through GAALIE mutations that increase the binding affinity to Fc-activating receptors. 7C6-GAALIE inhibits MICA/B shedding and potently triggers NK cell-effector functions against tumor cells. Furthermore, we establish a model of metastatic prostate cancer with which we formally demonstrate that 7C6-GAALIE is superior to 7C6 as wild-type human IgG1 in inhibiting metastases in Fc gamma receptor-humanized mice. In melanoma and leukemia models, 7C6-GAALIE had a therapeutic efficacy more consistent than that with 7C6 as wild-type human IgG1. Therefore, this study establishes the proof of concept of a next-generation anti-MICA/B antibody for cancer immunotherapy through an Fc optimization that enhances the antibody's ability to elicit NK cell-driven immunity.

INTRODUCTION

Malignant transformation can activate cellular stress pathways that, in response, trigger the expression of stress-induced proteins to mark transformed cells for clearance by natural killer (NK) cells. Two key examples are the major histocompatibility complex class I polypeptide-related sequences A (MICA) and B (MICB), here abbreviated as MICA/B.[1] They are commonly expressed by cancer cells and recognized by the NK group 2 member D (NKG2D) receptor, which promotes perforin and granzyme release and interferon-γ production by NK cells.[2,3] NKG2D is also expressed by T cells and provides co-stimulation.[4] However, cancer cells can evade NKG2D by shedding MICA/B from the surface through proteolytic cleavage.[5,6] Soluble MICA/B molecules, presumably produced by cancer cells, were detected in serum samples of several patients with advanced solid tumors or hematological malignancies.[7] To counteract this immune evasion mechanism, a previous study developed a panel of anti-MICA/B monoclonal antibodies (mAbs) that inhibited the shedding and retained MICA/B on the cellular surface without blocking NKG2D. 7C6 was one of them and the best characterized clone. 7C6 promoted NK cell-mediated killing of tumor cells in an NKG2D-dependent manner and engaged the CD16a fragment crystallizable (Fc) receptor to trigger antibody-dependent cellular cytotoxicity (ADCC). 7C6 also inhibited tumor growth in pre-clinical models.[8] Furthermore, pharmaceutical and biotechnology companies developed mAbs that, like 7C6, also inhibit MICA/B shedding.[9–11] One of them, CLN-619, recently transitioned to clinical trial phase, and preliminary analyses revealed that CLN-619 was well tolerated and generated objective responses with durable clinical benefits in patients with advanced solid tumors.[12] Given this initial success, another trial is testing CLN-619 in patients with refractory or relapsed multiple myeloma (NCT06381141).

Recent developments have revealed that Fcγ receptors play essential roles in the anti-tumor activity of antibody-based cancer immunotherapies by inducing NK cell and macrophage effector functions against antibody-opsonized tumor cells.[13] Consistent with that notion, human IgG1 (hIgG1) is an ideal format of anti-MICA/B antibodies because it preferentially binds Fcγ-activating receptors. 7C6-hIgG1 more potently induced NK cell-mediated cytotoxicity than an Fc-inert version of 7C6.[8]

© 2026 The Author(s). Published by Elsevier Inc.
This is an open access article under the CC BY license (http://creativecommons.org/licenses/by/4.0/).

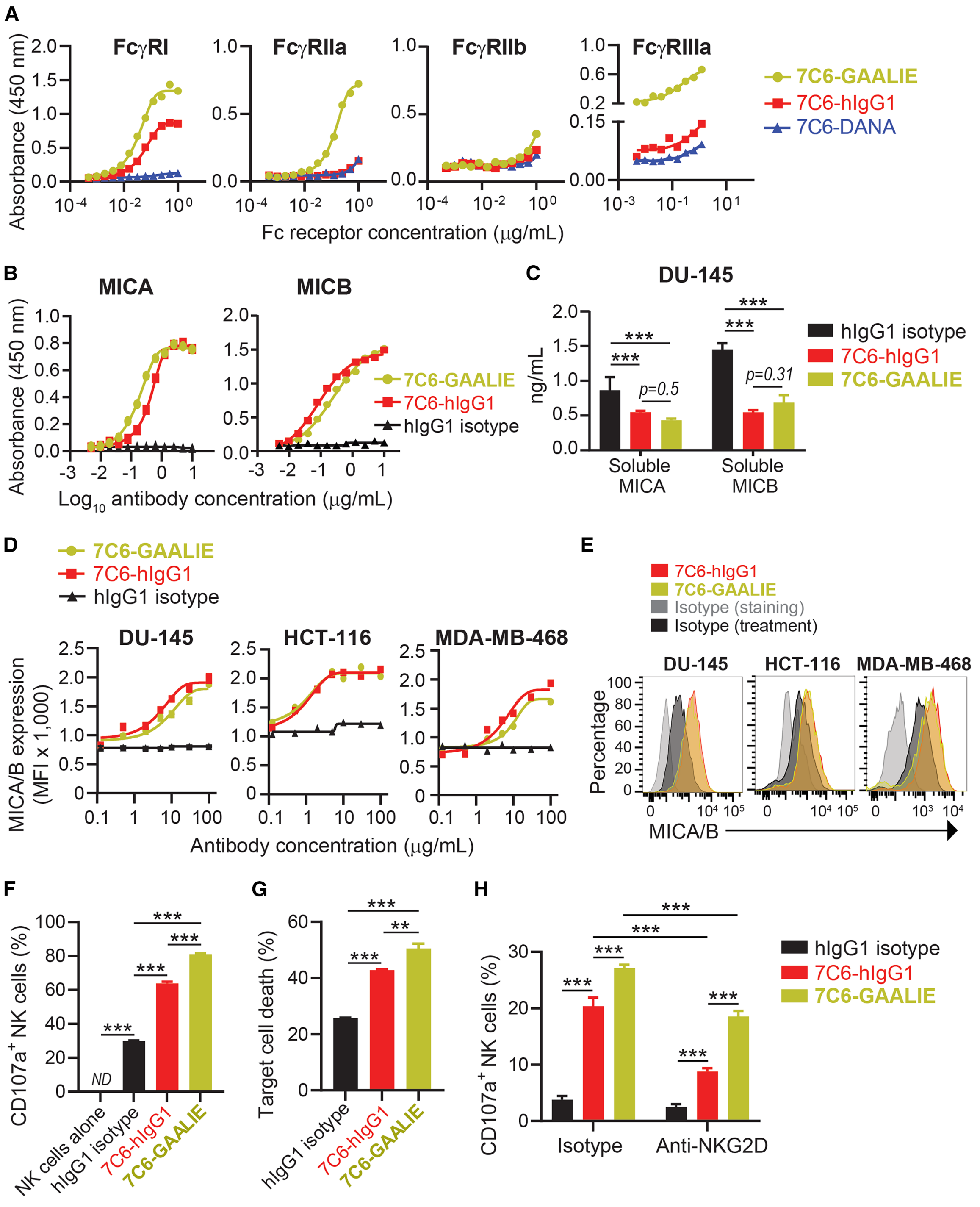

Figure 1. Profile of binding to Fcγ receptors and more potent induction of NK cell-mediated cytotoxicity against tumor cells by the inhibition of MICA/B shedding with Fc-enhanced 7C6

(A) The indicated versions of 7C6 were immobilized on the bottom of multi-well plates and incubated with biotinylated Fcγ receptors, peroxidase-labeled streptavidin, TMB, and sulfuric acid. Absorbance at 450 nm was analyzed in plate reader.

(B) ELISA for the binding of 7C6 to MICA (left graph) or MICB (right graph). Recombinant MICA or MICB proteins were immobilized on the bottom of multi-well plates and incubated with the indicated antibodies and biotinylated anti-human IgG secondary antibody. Reaction was revealed as in (A).

(C) ELISA-based quantification of soluble MICA/B molecules shed in supernatants of DU-145, which were treated for 24 h with 10 μg/mL of the indicated antibodies.

(D and E) The indicated cell lines were treated for 24 h with the indicated antibodies, and surface MICA/B expression was analyzed by flow cytometry.

(legend continued on next page)

However, although hIgG1 triggers ADCC, it binds with relatively low affinity to the only Fcγ-activating receptor in human NK cells, i.e., CD16a.[13] Furthermore, several correlative studies demonstrated that patients with a higher-affinity CD16a polymorphic variant responded better to therapies with Fc-enabled tumor cell-opsonizing antibodies.[14–16] The binding affinity can also be artificially increased by Fc engineering, for example, with the introduction of three point mutations in the Fc region: G236A, A330L, and I332E (GAALIE). GAALIE-mutant hIgG1 binds with higher affinity to all three Fcγ-activating receptors (CD16a, CD32a, and CD64), while maintaining the natural low affinity of hIgG1 to the Fcγ-inhibitory receptor (CD32b). As a consequence of this enhanced binding, GAALIE-mutant mAbs for virus antigens, for example, displayed greater therapeutic efficacy in influenza or severe acute respiratory syndrome coronavirus 2 virus models.[17,18] Furthermore, anti-CD47 mAbs, which block a "do not eat me" signal in tumor cells that otherwise would inhibit macrophages, displayed superior anti-tumor activity when expressed with GAALIE mutations.[19] Therefore, the Fc region of antibodies can be modified to increase the binding affinity to Fcγ receptors and modulate leukocyte effector functions.

Anti-MICA/B antibodies that inhibit the shedding are ideal candidates for Fc-effector optimization because they not only promote NKG2D engagement but also engage Fcγ receptors. We, therefore, hypothesized that the therapeutic efficacy of anti-MICA/B antibodies is enhanced through Fc optimization with GAALIE mutations. In this study, we developed a GAALIE-mutant version of 7C6 and compared it against 7C6 with the wild-type hIgG1 Fc, which also engages Fc gamma receptors but with a lower affinity than that for GAALIE-mutant Fc. We used models of metastatic prostate cancer, melanoma, and leukemia to demonstrate that GAALIE-mutant hIgG1 is a clinically actionable Fc variant of anti-MICA/B antibody for better eliciting NK cell-driven immunity.

RESULTS

Development and validation of the GAALIE-mutant version of 7C6

Although hIgG1 knowingly binds with higher affinity to the human Fcγ-activating receptors compared to other human IgG subclasses, such preferential binding can be increased by Fc engineering with GAALIE mutations.[13] In this study, we generated a GAALIE-mutant version of 7C6 and compared it against the previous version that had the wild-type Fc (i.e., 7C6-hIgG1). By biochemical assays, we validated that GAALIE mutations made 7C6 bind with higher affinity to Fcγ-activating receptors (FcγRI, FcγRIIa, and FcγRIIIa, also known as CD64, CD32a, and CD16a, respectively), while they maintained a low affinity of hIgG1 to the inhibitory receptor (FcγRIIb, CD32b) (Figure 1A). In these assays, 7C6 with the D265A and N297A (DANA) mutations served as a negative control, because DANA mutations knowingly abrogate the binding to Fcγ receptors.[13] On the other hand, both 7C6-GAALIE and 7C6-hIgG1 bound to a similar extent MICA and MICB (Figure 1B). We then applied 7C6-hIgG1 and 7C6-GAALIE to MICA/B shedding assays. Both mAbs inhibited the shedding of MICA/B molecules in the supernatants of DU145 prostate cancer cell line (Figure 1C). Furthermore, 7C6-GAALIE and 7C6-hIgG1 had comparable efficacy in stabilizing surface MICA/B in human cancer cell lines (Figures 1D and 1E). We then used the DU-145 prostate cancer cell line as "target cells" in NK cell effector function assays, including a flow cytometry-based cytotoxicity assay (Figure S1A). Although 7C6-hIgG1 induced NK cell degranulation and target cell killing, 7C6-GAALIE induced these same NK cell effector functions to a greater extent (Figures 1F and 1G). Neither 7C6-GAALIE nor 7C6-hIgG1 directly induced DU-145 cell death in the absence of NK cells (Figure S1B). Although NK cell viability was reduced in the co-cultures, as determined by comparing against NK cell alone, such an effect was not amplified by 7C6-hIgG1 or 7C6-GAALIE (Figure S1C). By Incucyte-based imaging, we observed that calcein-labeled DU145 formed cell blebs and released calcein in the supernatant after co-culture with NK cells, indicating NK cell-mediated cytotoxicity (Figures S2A and S2B). The calcein release was more evident by Incucyte in the co-cultures that were treated with 7C6-hIgG1 or 7C6-GAALIE (Figure S2A). Furthermore, 7C6-hIgG1 and 7C6-GAALIE reprogramed NK cell gene expression, with hundreds of genes being differentially expressed, as determined by RNA sequencing data analyses of human NK cells that were co-cultured with DU-145 (Figures S3A–S3E). Conceptually, 7C6 inhibits MICA/B shedding to promote NKG2D recognition of tumor cells.[8] Consistent with that notion, we detected contribution by NKG2D in a degranulation assay, whereby antibody-mediated NKG2D blockade lowered the percentage of CD107a^{+} NK cells after co-culture with DU-145 cells that were treated with 7C6-hIgG1 or 7C6-GAALIE (Figure 1H). Therefore, we developed the Fc-optimized version of an anti-MICA/B antibody (7C6-GAALIE) and validated that it more potently induced ADCC.

The combined administration of 7C6-GAALIE with romidepsin induced MICA/B upregulation by cancer cells

MICA/B expression is induced by not only cellular stress but also certain epigenetic therapies.[1] Romidepsin is a histone deacetylase (HDAC) inhibitor that, when administered together with 7C6, induces surface MICA/B expression in human leukemia cells.[20] Romidepsin induced MICA/B mRNA expression in the human prostate cancer cell line (Figure S4). Romidepsin plus 7C6 as hIgG1 or GAALIE upregulated surface MICA/B expression in human cancer cell lines, although the effects were variable depending on the cell lines tested (Figures 2A and 2B). We, therefore,

(F–H) DU-145 was pre-treated for 24 h with the indicated antibodies and used as "target cells" in NK cell effector function assays at a 5:1 NK-to-target-cell ratio. NK cell CD107a externalization (F and H) and target cell death (G) were analyzed by flow cytometry. ND, not detected. Two donors were used in (F and G), and another two donors were used in (H).
Data represent three independent experiments (A–H), are mean ± standard deviation (SD) (C and D) or standard error (SE) (F–H) of technical quadruplicates (D) or technical triplicates (C, F–H), and were analyzed by non-linear regression (A, B, and D) or two-way ANOVA with Bonferroni's test (C and F–H). $^{**}p < 0.01$, $^{***}p < 0.001$. The histograms in (E) show MICA/B expression levels with the antibodies at 100 μg/mL. See also Figures S1–S3.

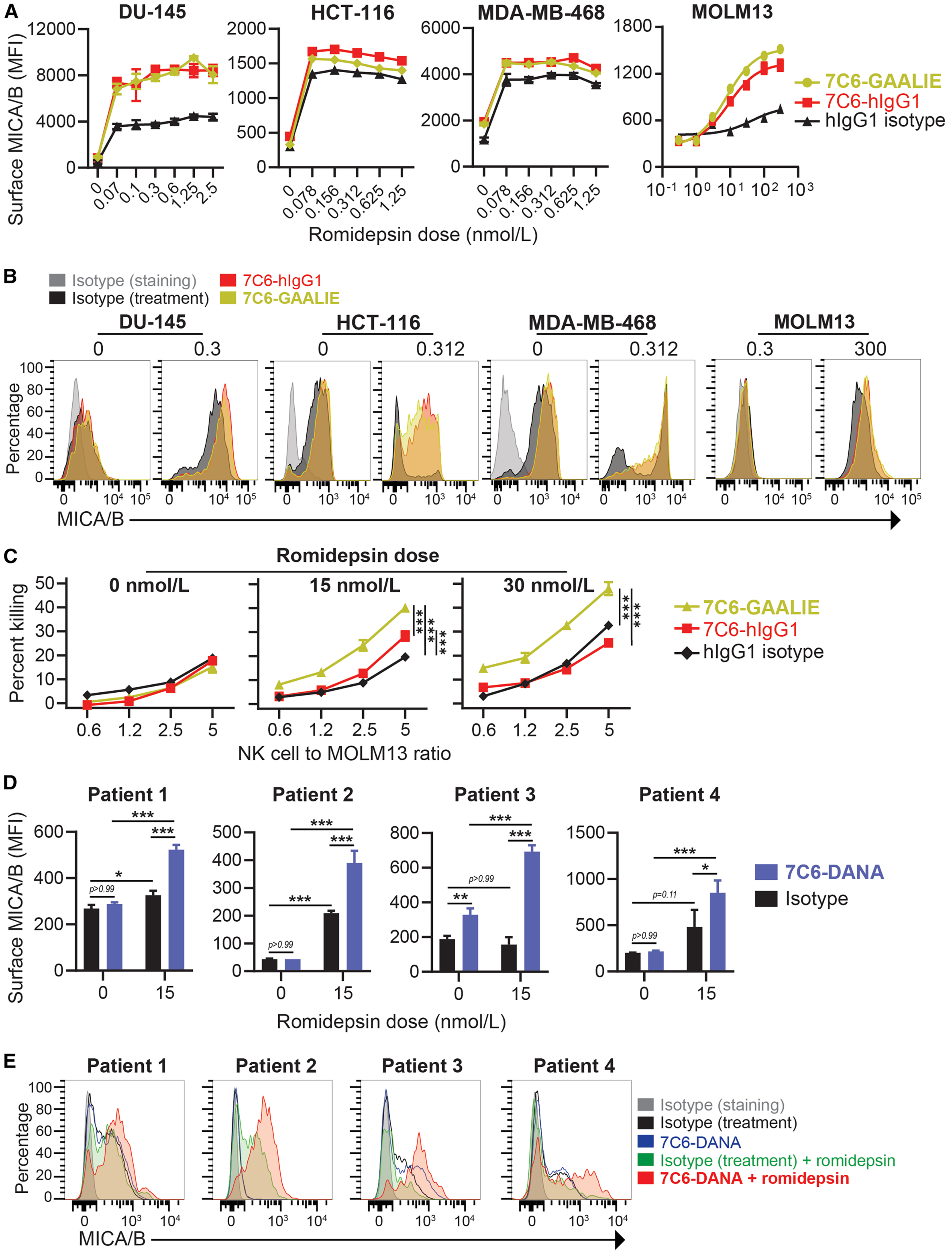

Figure 2. Romidepsin plus 7C6 increase surface MICA/B expression and promote NK cell-mediated cytotoxicity

(A–E) The indicated cell lines (A and B), MOLM13 that was later used as "target cells" (C), and primary leukemia cells from AML patients (D and E) were treated for 24 h with the indicated concentrations of romidepsin plus the indicated antibodies at 10 μg/mL. Subsequently, surface MICA/B was analyzed by flow cytometry

(legend continued on next page)

speculate that the human *MICA* and *MICB* genes are epigenetically regulated by the HDACs 1 and 2, which, on the other hand, can be inhibited by romidepsin, a narrow-spectrum HDAC inhibitor. Of note, romidepsin is particularly useful to enable 7C6-mediated opsonization of cancer cells that otherwise would have a low expression of MICA/B by a mechanism that involves downregulation at the mRNA level. The MOLM13 acute myeloid leukemia (AML) cell line is an example (Figures 2A and 2B). We then used MOLM13 as target cells in an ADCC assay (Figure S5A). We found that 7C6-GAALIE triggered NK cell-mediated killing of romidepsin-treated MOLM13, whereas 7C6-hIgG1 had a minor effect (Figure 2C). Romidepsin by itself did not kill MOLM13 at the tested doses (Figure S5B). Furthermore, 7C6-hIgG1 and 7C6-GAALIE increased the interferon-gamma production by NK cells that were co-cultured with MOLM13, and these effects were potentiated by romidepsin at greater degrees with 7C6-GAALIE (Figure S5C). Conceptually, GAALIE mutations optimize NK cell effector functions through Fc receptors but we also detected contribution by NKG2D in CD107a degranulation assays, whereby antibody-mediated NKG2D blockade lowered the percentages of CD107a^{+} NK cells upon co-culture with 7C6-GAALIE plus romidepsin-treated MOLM13 (Figure S5D). Furthermore, romidepsin and 7C6 increased surface MICA/B expression in primary leukemia cells that were isolated from the peripheral blood of four patients with *de novo* AML (Figures 2D and 2E). Therefore, the MICA/B expression upregulation after romidepsin and 7C6 combined administration was confirmed with 7C6-GAALIE, which enabled ADCC against an AML cell line.

Since 7C6-GAALIE had a better binding to all Fc gamma-activating receptors (Figure 1A), including CD32a and CD64 that are not expressed by NK cells, we analyzed the ability of 7C6-GAALIE to induce antibody-dependent cellular phagocytosis (ADCP) by human monocyte-derived macrophages. We used a target cell panel with three leukemia cell lines, because, in addition to MOLM13, we also identified another two human AML cell lines that upregulate MICA/B expression in response to the combined treatment with 7C6 + romidepsin (Figure S6A). Although 7C6-hIgG1 and 7C6-GAALIE induced, to a similar extent, ADCP by human macrophages against romidepsin-treated MOLM13 and SKM-1, only 7C6-GAALIE triggered ADCP against romidepsin-treated OCI-AML-3 (Figure S6B). Therefore, both versions of 7C6 (hIgG1 and GAALIE), for the most part, triggered ADCP by macrophages against romidepsin-treated leukemia cells, but such an effect is target cell dependent.

Establishment of a prostate cancer metastasis model to investigate the therapeutic efficacy of 7C6

Metastasis is a critical determinant of survival outcomes in solid tumors, where NK cells can have a prominent role, and MICB shedding was associated with disease progression.[7,21] NK cells control metastatic dissemination by immunosurveillance for metastatic cancer cells.[22] This is particularly relevant in metastatic prostate cancer, where resistance to immunotherapy remains a hallmark.[23] We, therefore, established a syngeneic metastasis model by isolating a tumor cell line (i.e., NPK) from a spontaneous bone metastasis of a genetically engineered mouse model of prostate cancer, which was described.[24] NPK cells expressed murine NKG2D ligands and classical and non-classical major histocompatibility class I molecules (Figures 3A and 3B). Furthermore, NPK cells were syngeneic in C57BL/6 mice and formed tumors in the lungs after intravenous inoculation. Antibody-mediated NKG2D blockade and NK cell depletion increased lung metastasis formation after the intravenous inoculation of NPK cells in C57BL/6 wild-type mice, with NK cell depletion having the strongest effect (Figures 3C, 3D, and S7A). On the other hand, although mice have NKG2D that cross-reacts with human NKG2D ligands, mice have no orthologous genes that would correspond to human *MICA* or *MICB*. To bypass that limitation, we developed a version of the NPK cells by lentiviral transduction for human MICB constitutive expression. We used MICB allele 005, which is the most common polymorphic variant in humans.[25] The mouse Fc-enabled version of the anti-MICA/B antibody (i.e., 7C6-mIgG2a) stabilized surface MICB in NPK-MICB cells (Figure 3E). 7C6-mIgG2a also inhibited the formation of tumors in the lungs, decreased the amount of soluble MICB shed in the sera, and prolonged the survival of wild-type mice after the inoculation with NPK-MICB cells (Figures 3F–3I and S7B). Therefore, NPK-MICB served as a prostate cancer metastasis model to probe the NK cell biology and investigate the therapeutic efficacy of anti-MICA/B antibodies.

Fc-enabled 7C6 inhibited NPK-MICB metastases in an Fc effector-dependent manner, and its ability to do so was increased by GAALIE mutations

To evaluate the *in vivo* contribution from Fc effector functions and NKG2D signaling after MICA/B antibody treatment, we compared 7C6-mIgG2a with the Fc-inert version (7C6-DANA) with or without NKG2D blockade. 7C6-mIgG2a, but not 7C6-DANA, inhibited NPK-MICB metastases in wild-type C57BL/6 mice, whereas NKG2D blockade interfered with such a biological effect (Figure 4A). We confirmed that 7C6-mIgG2a-treated mice had less serum MICB molecules, whereas 7C6-DANA did not significantly decrease the amount of soluble MICB molecules shed in the sera (Figure 4B). Furthermore, because both 7C6-hIgG1 and 7C6-GAALIE stabilized MICB surface expression in NPK-MICB cells to the same extent *in vitro* (Figure S8A), we applied the NPK-MICB model to Fcγ receptor-humanized (hFcR) mice with 7C6-GAALIE and 7C6-hIgG1 treatments (Figure 4C). hFcR mice are immunocompetent and had the murine Fcγ receptors genetically replaced with the human Fcγ receptors.[26] NPK-MICB is syngeneic in hFcR mice. Although both antibodies had trends to decrease the formation of tumors in the lungs of hFcR mice after the intravenous inoculation with NPK-MICB cells, 7C6-GAALIE significantly decreased the amount of metastasis when compared against isotype or 7C6-hIgG1 (Figures 4C and 4D). Furthermore, the concentrations of

(A and B, D, and E) or MOLM13 was used as "target cells" in NK cell-mediated killing assays with target cell death analyzed by flow cytometry (C). Data represent three independent experiments (A–C) with three different NK cell donors (C), are mean ± SE (A) or SD (C and D) of technical triplicates or duplicates (A and D) or quadruplicates (C), and were analyzed by non-linear regression (A, MOLM13) or two-way ANOVA with Bonferroni's test (C and D). *$p < 0.05$, **$p < 0.01$, ***$p < 0.001$. See also Figures S4–S6.

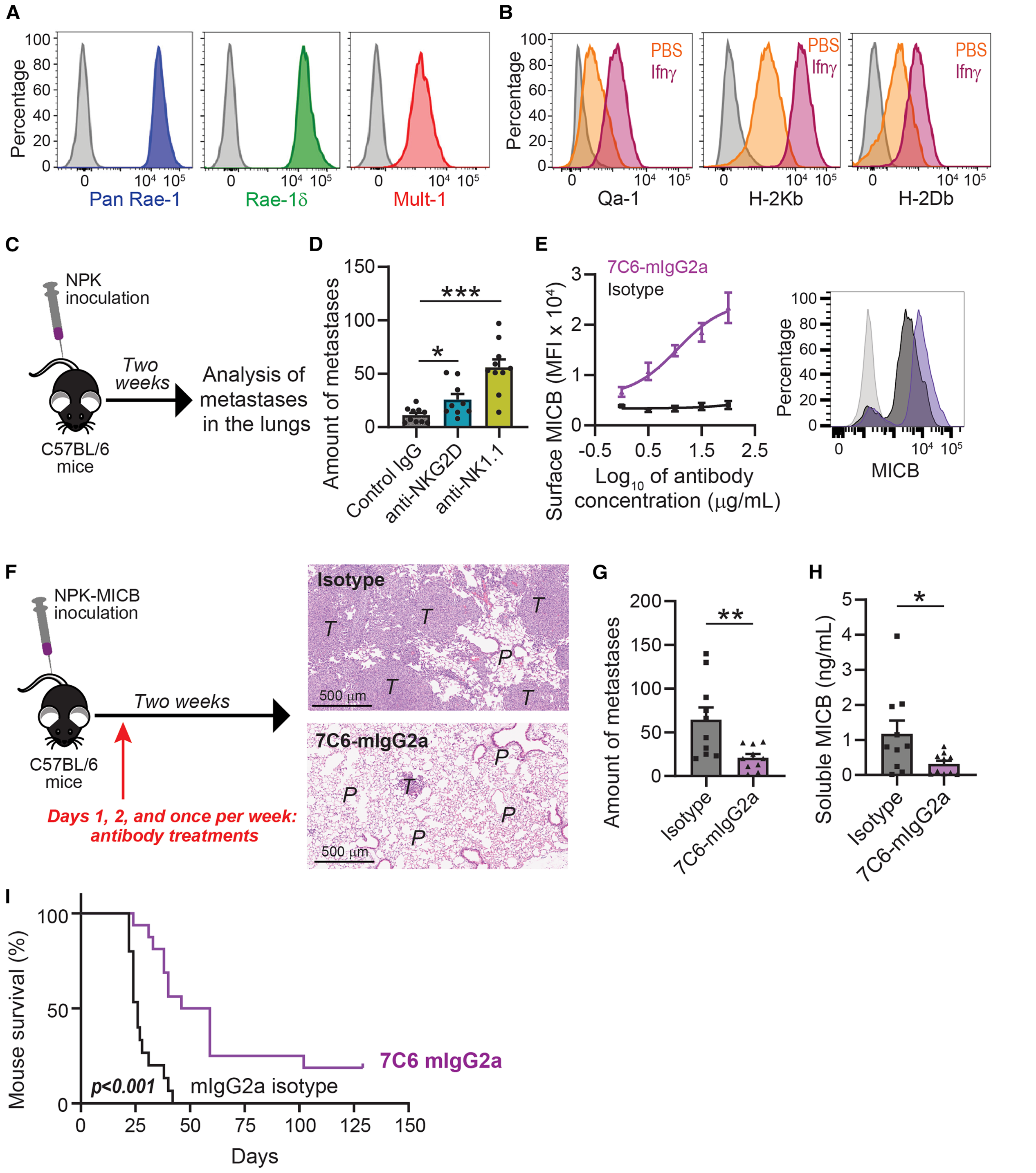

Figure 3. Establishment and validation of a mouse prostate cancer metastasis model

(A) Flow cytometry analyses of NKG2D ligand expression in NPK cells. The isotype control histogram is in gray color.

(B) NPK cells were treated for 24 h with 10 ng/mL mouse recombinant interferon-γ (Ifnγ), followed by flow cytometry analyses of classical and non-classical MHC class I molecule expression.

(C) Illustration of the mouse prostate cancer metastasis model, whereby NPK cells were inoculated intravenously and the analysis of metastases in the lungs was performed after 3 weeks by Indian ink infusion and stereomicroscopy.

(D) C57BL/6 mice were treated with a control IgG, anti-NKG2D antibody, or anti-NK1.1 antibody on days −1, 0, and once per week relative to NPK inoculation. Metastases in the lungs were analyzed 3 weeks after NPK inoculation. Control IgG n = 10, anti-NKG2D n = 9, anti-NK1.1 n = 10.

(legend continued on next page)

soluble MICB in the serum were lower for both antibody treatments, attesting to the antibodies' comparable abilities to inhibit MICB shedding (Figure 4E). 7C6-GAALIE significantly prolonged the survival of hFcR mice that were submitted to the prostate cancer metastasis model (Figure 4F). In *ex vivo* assays, we detected more degranulation by lung NK cells from hFcR mice that underwent the NPK-MICB model and were treated with 7C6-GAALIE, compared to isotype and 7C6-hIgG1 (Figure S8B). Therefore, 7C6-mIgG2a required Fc receptor engagement and NKG2D signaling for optimally inhibiting NPK-MICB metastases in wild-type mice, and 7C6-GAALIE, compared to 7C6-hIgG1, better inhibited NPK-MICB metastases in hFcR mice and only 7C6-GAALIE significantly prolonged mouse survival.

7C6-GAALIE had a more consistent efficacy than 7C6-hIgG1 in melanoma and leukemia models

The NPK-MICB prostate cancer metastasis model, which was shown above, enabled the proof of concept that 7C6-GAALIE had a superior ability to inhibit tumor growth *in vivo*, compared against 7C6-hIgG1. On the other hand, MICA/B expression and shedding are clinically relevant in a variety of solid tumor types besides prostate cancer, as well as in hematological malignancies like AML.[7] In a previous study, we established a melanoma model with B16F10 melanoma cells that were genetically engineered to express human MICA or MICB.[8] Since B16F10 is syngeneic in hFcR mice, we applied 7C6-GAALIE and 7C6-hIgG1 to this melanoma metastasis model (Figure 5A). 7C6-GAALIE was slightly more efficient in inhibiting the formation of B16F10-MICB melanoma metastases in the lungs of hFcR mice compared to 7C6-hIgG1, but both antibodies were similarly effective in inhibiting the formation of B16F10-MICA metastases (Figures 5A, 5B, S9A, and S9B). We also analyzed the toxicity profile of 7C6-hIgG1 and 7C6-GAALIE in the mouse prostate and melanoma metastasis models; neither 7C6-hIgG1 nor 7C6-GAALIE altered serum biomarkers of kidney and liver functions or caused histopathologic alterations in the heart, liver, kidney, or spleen of hFcR mice that underwent the prostate cancer metastasis model (Figures S10A and S10B). Furthermore, neither 7C6-hIgG1 not 7C6-GAALIE altered serum biomarkers of kidney and liver functions or weight of hFcR mice that underwent the B16F10-MICA metastasis model (Figures S10C and S10D). We also analyzed the *in vitro* migratory and invasive capacity of B16F10-MICA, NPK-MICB, and DU-145, and discovered that neither 7C6-hIgG1 nor 7C6-GAALIE altered the *in vitro* migratory or invasive activity of those three cell lines (Figure S11A–S11E, S12A–S12D, and S13A–S13D). Furthermore, C1498-MICB is a mouse leukemia cell line that was engineered to express human MICB.[20] Since C1498-MICB is also syngeneic in hFcR mice, we used it as a mouse AML model to investigate the *in vivo* anti-leukemia activity of 7C6-hIgG1 and 7C6-GAALIE (Figure 5C). In general, both antibodies inhibited leukemia outgrowth in the blood, but the efficacy of 7C6-GAALIE was more consistent in the blood and statistically confirmed in the bone marrow (Figures 5D, S14A, and S14B). Furthermore, we analyzed blood and bone marrow NK cells from hFcR mice that were subjected to the C1498-MICB model. 7C6-GAALIE reduced the absolute numbers of blood NK cells (Figures S15A and S15B). On the other hand, there were increases in the absolute numbers of bone marrow NK cells after treatment with 7C6-hIgG1 and 7C6-GAALIE (Figures S15C and S15D). We did not find major differences in the expression of CD16a or NKG2D, except for a downregulation of NKG2D in bone marrow NK cells of 7C6-GAALIE-treated mice (Figures S15B and S15D). To complement the mouse AML model, we developed a human AML model whereby the MOLM13 cell line was transduced with a lentivirus for MICB constitutive expression that bypasses the need for romidepsin and, thus, facilitates *in vivo* studies. 7C6-GAALIE and 7C6-hIgG1 inhibited MICB shedding by MOLM13-MICB (Figures S16A and S16B). For *in vivo* studies, we used FcResolv hIL-15 NOG mice, which lack endogenous NK and T cells, have murine *Fcgr2b* and *Fcer1g* genes knocked out to abolish endogenous Fcγ receptor-driven myeloid immunity, and express human IL-15 to support human NK cell engraftment.[27] Fresh human NK cells from volunteer donors were infused into these mice, which were then inoculated with MOLM13-MICB and treated with antibodies (Figure 5E), enabling us to directly assess whether 7C6-GAALIE can harness human NK cells to inhibit human AML progression. We found that 7C6-GAALIE significantly reduced the absolute number of leukemia cells in the blood, whereas 7C6-hIgG1 showed only a non-significant trend toward reduction (Figures 5F and S16C). A limitation of this experiment, however, was that MOLM13-MICB cells did not consistently engraft in the bone marrow of FcResolv hIL-15 NOG mice (Figures 5F and S16D). Similar to our observations in the hFcR AML model, both 7C6-GAALIE and, in this specific setting, 7C6-hIgG1 reduced the absolute number of human NK cells in the blood (Figures S17A and S17B), and without causing major alterations in CD16a or NKG2D expression levels on these cells *in vivo* (Figures S17C and S17D). Overall, these findings indicate that 7C6-GAALIE showed more consistent therapeutic activity in melanoma and AML models than 7C6-hIgG1.

(E) NPK-MICB cells were treated for 24 h with the indicated concentrations of antibodies and surface MICB was analyzed by flow cytometry. The histograms show MICB expression at an antibody dose of 100 μg/mL. Gray, isotype (staining); black, isotype (treatment); and purple, 7C6-mIgG2a.
(F) Illustration of the NPK-MICB model that enabled analysis of 7C6-mIgG2a therapeutic efficacy. Antibodies were given at dose of 0.2 mg per mouse on days 1 and 2, followed by a dose of 0.1 mg per mouse once per week until euthanasia. The histopathologic pictures represent 5 mice per antibody group. *T*, tumor; *P*, lung parenchyma. Scale bars represent 500 μm.
(G) Quantification of metastases in the lungs on day 21. mIgG2a isotype $n = 10$, 7C6-mIgG2a $n = 10$.
(H) Analyses of soluble MICB shed in the sera of mice, which underwent cheek bleeding on day 15. mIgG2a isotype $n = 10$, 7C6-mIgG2a $n = 10$.
(I) Analyses of mouse survival. mIgG2a isotype $n = 15$, 7C6-mIgG2a $n = 15$.
Data represent three (A, B, and E) or two (I) or are a pool of two (D and G–H) independent experiments, are mean ± SE (D, E, G, and H) of technical triplicates (E), and were analyzed by one-way ANOVA with Bonferroni's test (D), non-linear regression (E), two-tailed unpaired Student's *t* test (G and H), or Mantel-Cox test (I). Each dot represents one mouse (D, G, and H). *$p < 0.05$, **$p < 0.01$, ***$p < 0.001$. See also Figure S7.

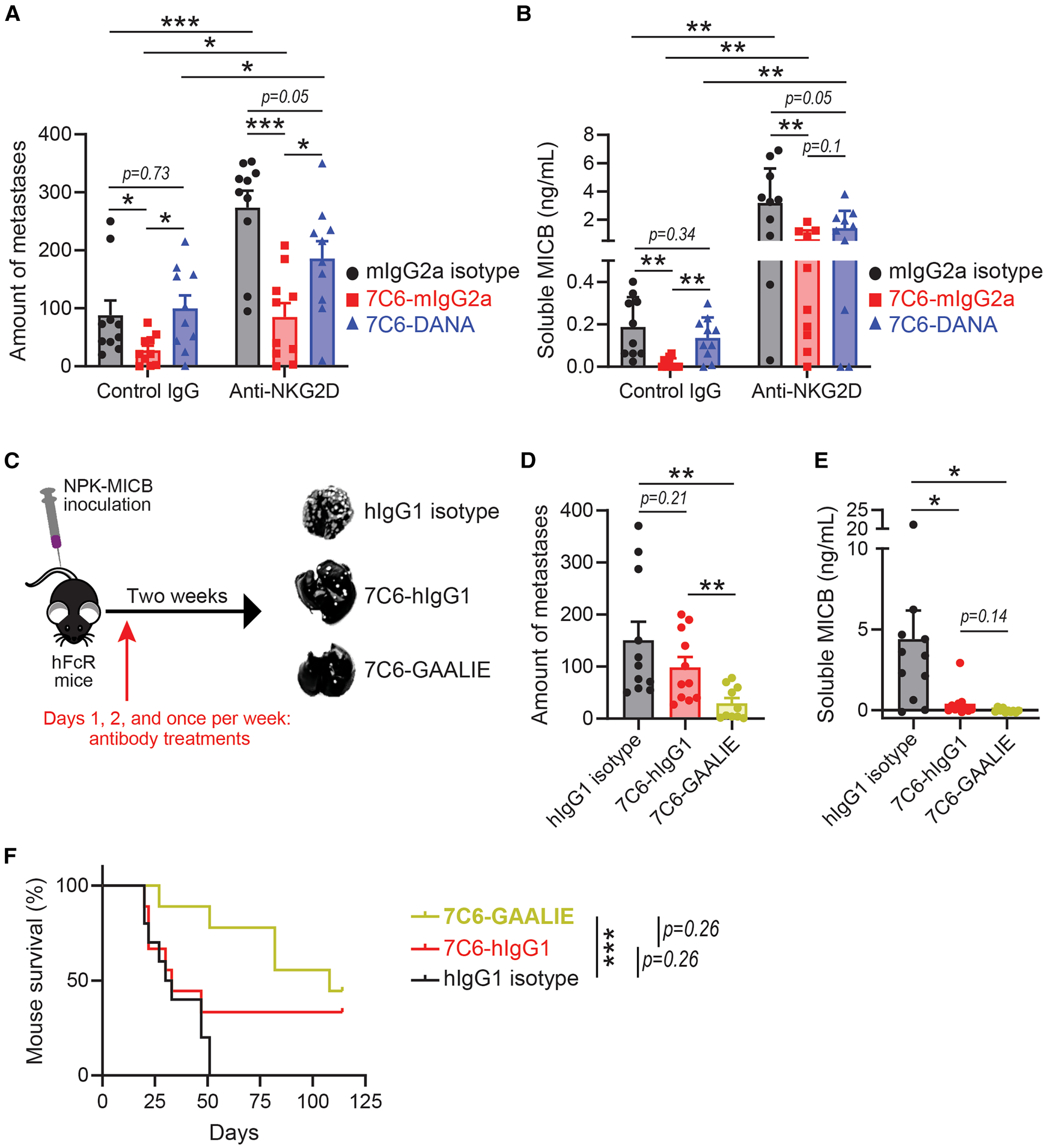

Figure 4. The ability of Fc-enabled 7C6 to inhibit prostate cancer metastases is Fc receptor mediated and enhanced with GAALIE mutations

(A and B) C57BL/6 mice were inoculated intravenously with the mouse prostate cancer cell line, which marks day 0 of the experiment. On days 1, 2, and once per week, the mice were treated with mIgG2a isotype, 7C6-mIgG2a, or 7C6-DANA. On days −1, 0, and 7, the mice were treated with control IgG or anti-NKG2D. Analyses of tumors in the lungs (A) and soluble MICB in the sera (B) were conducted on day 14. Isotype $n = 10$, 7C6-hIgG1 $n = 10$, 7C6-GAALIE $n = 10$.

(C) Illustration of the mouse prostate cancer metastasis model in hFcR mice, with antibody treatments at a dose of 0.2 mg per mouse on days 1 and 2 and 0.1 mg per mouse once per week.

(D) Quantification of metastases in the lungs, by Indian ink and stereomicroscopy. Isotype $n = 11$, 7C6-hIgG1 $n = 11$, 7C6-GAALIE $n = 10$.

(E) Quantification of soluble MICB shed in serum samples. Isotype $n = 11$, 7C6-hIgG1 $n = 9$, 7C6-GAALIE $n = 9$.

(F) Analysis of hFcR mouse survival, with the same experimental setting as in (C) but with euthanasia when mice reach the human endpoint. Isotype $n = 10$, 7C6-hIgG1 $n = 11$, 7C6-GAALIE $n = 10$.

Data are a pool of two independent experiments (A, B, and D–F), are mean ± SE (A, B, D, and E), and were analyzed by two-tailed unpaired Student's *t* test with the comparisons between the indicated groups (A, B, D, and E) or Mantel-Cox test (F). $^*p < 0.05$, $^{**}p < 0.01$, $^{***}p < 0.001$. See also Figure S8.

CellPress
OPEN ACCESS

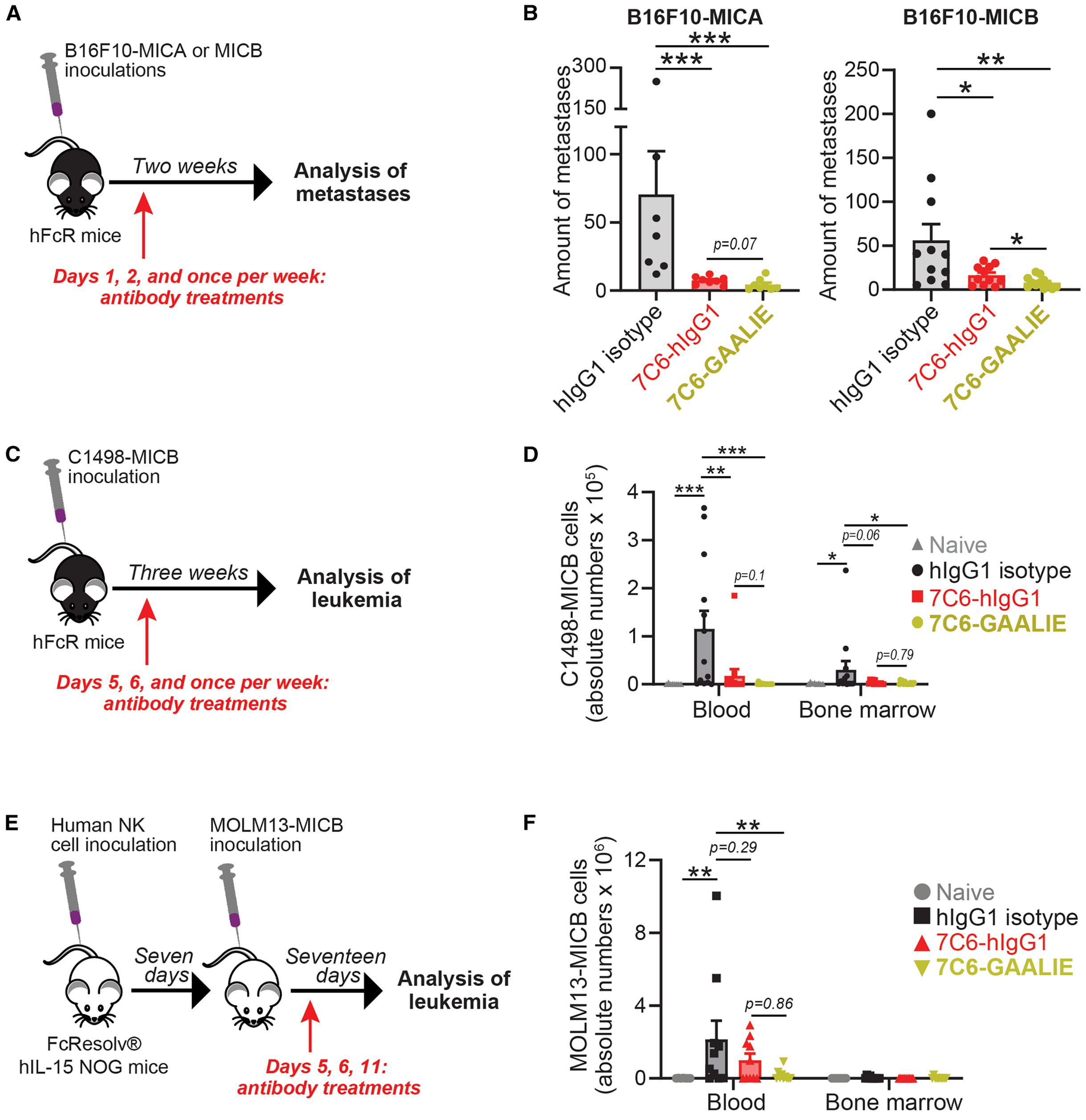

Figure 5. Therapeutic efficacy of 7C6-hIgG1 and 7C6-GAALIE in mouse and human tumor models

(A) Illustration of the B16F10-MICA and B16F10-MICB mouse melanoma metastasis models in hFcR mice. Antibodies were administered at doses of 0.2 mg per mouse per injection.

(B) Quantification of metastases in the lungs of hFcR mice 2 weeks after tumor cell inoculation. In the B16F10-MICA model: hIgG1 isotype $n = 7$, 7C6-hIgG1 $n = 8$, and 7C6-GAALIE $n = 8$. In the B16F10-MICB model: isotype $n = 11$, 7C6-hIgG1 $n = 11$, and 7C6-GAALIE $n = 12$.

(C) Illustration of the mouse AML model in hFcR mice. Antibodies were administered at doses of 0.2 mg per mouse per injection.

(D) Flow cytometry-based analyses of C1498-MICB leukemia cells in the blood and bone marrow of hFcR mice on day 21. Naive $n = 8$, isotype $n = 13$, 7C6-hIgG1 $n = 13$, and 7C6-GAALIE $n = 14$.

(E) Illustration of the human AML model in murine Fcγ-receptor-deficient human interleukin 15 transgenic mice that were reconstituted with human primary NK cells. Antibody treatments on the indicate days were at doses of 0.2 mg per mouse.

(F) Analyses, by flow cytometry, of MOLM13-MICB cells in the blood and bone marrow on day 17. Naive $n = 7$, isotype $n = 10$, 7C6-hIgG1 $n = 10$, and 7C6-GAALIE $n = 10$. Each experiment was done with different NK cell donors, thus two donors total.

Data are a pool of two (B, D, and F) independent experiments, are mean ± SE (B, D, and F), and were analyzed by multiple two-tailed Mann-Whitney tests (B and D) or two-way ANOVA with Bonferroni's test (F). Each dot represents one mouse (B, D, and F). $*p < 0.05$, $**p < 0.01$, $***p < 0.001$. See also Figures S9–S17.

DISCUSSION

Anti-MICA/B antibodies that inhibit the shedding hold promise for cancer immunotherapy and recently transitioned to phase 1 clinical trials, and one of them has revealed that they were well tolerated by patients with cancer and generated durable clinical benefits.[12] However, the ongoing trials use MICA/B antibodies as wild-type hIgG1. Our study compared 7C6-hIgG1 and 7C6-GAALIE in tumor models, which revealed remarkable differences in efficacy. For the most part, 7C6-hIgG1 inhibited AML and melanoma development, but 7C6-GAALIE had a more consistent therapeutic efficacy in the AML models, whereas it significantly inhibited metastases to a greater extent than that for 7C6-hIgG1 in the prostate cancer and melanoma models. The aggregate of *in vitro* experiments also consistently demonstrated that 7C6-GAALIE was superior to 7C6-hIgG1 in promoting ADCC against cancer cell lines. Although both antibodies had comparable abilities to inhibit MICA/B shedding, 7C6-GAALIE bound Fcγ-activating receptors with higher affinity, as expected for GAALIE-mutant antibodies. Therefore, this study highlighted the importance of Fcγ receptor engagement in the mechanism of action of anti-MICA/B antibodies and that 7C6-GAALIE is the most efficacious version for potently eliciting NK cell-driven immunity against cancers.

Not only GAALIE mutations but also non-fucosylation represent efficient means to increase the Fc effector functions of, for example, tumor cell-opsonizing antibodies.[28] Although antibodies with enhanced Fc may increase immunotoxicity, MICA/B proteins are stress-induced autoantigens with restricted expression in normal tissues.[1] They are detectable, for example, only in the small intestine mucosa of celiac patients exposed to gluten or in the liver of patients with hepatitis C.[29,30] This selective expression suggests that Fc-enhanced antibodies targeting MICA/B can effectively activate immune effector functions while minimizing on-target/off-tumor effects.

Certain Fc-enabled antibodies, like rituximab for B cell lymphoma and daratumumab for multiple myeloma, have dual impacts on NK cells: they trigger ADCC but decrease the numbers of circulating NK cells.[31,32] However, blood NK cell numbers increase after the end of daratumumab treatments and do not correlate with clinical outcomes.[32] Daratumumab decreases NK cell numbers by fratricide, whereby NK cells kill one another due to low expression levels of CD38, the antigen recognized by daratumumab.[33] Hence, one could assume that 7C6-hIgG1 and 7C6-GAALIE lowered the blood NK cell numbers by fratricide, which would be particularly relevant for human NK cells in FcResolv hIL-15 NOG mice. However, we observed decreases in NK cell numbers also in hFcR mice, which lack the human *MICA* or *MICB* genes. An alternative explanation is that 7C6-hIgG1 and 7C6-GAALIE redistributed NK cells to other organs. Consistent with that possibility, we detected higher numbers of bone marrow NK cells in hFcR mice that were treated with 7C6-hIgG1 and 7C6-GAALIE. Although the increase in bone marrow NK cell numbers could mean compensatory production due to more demand or "emergency NK cell poiesis," another study demonstrated that intermittent fasting caused similar redistribution to the bone marrow, where NK cells received stimulation by myeloid cells to enhance anti-tumor immunity.[34] Therefore, the mechanisms by which 7C6-hIgG1 and 7C6-GAALIE partially depleted blood NK cells remain to be fully elucidated, but it is probably not mediated only by fratricide.

MICA/B shedding represents a highly relevant therapeutic target in cancer immunology. In prostate cancer, for example, MICA/B proteins are absent in benign tissue but become highly expressed in high-grade prostatic intraepithelial neoplasia. On the other hand, membrane-bound MICA/B proteins decrease in advanced prostate cancer, followed by an increase in soluble MICA/B in patient serum.[21] In AML, high expression levels of MICA/B in leukemia cells correlate with longer survival and lower relapse rates after chemotherapy, whereas serum samples from AML patients have an average soluble MICA/B concentration that is 10-fold higher than that for healthy subjects.[35,36] The clinical significance of MICA/B release has also been demonstrated in, for example, non-Hodgkin lymphoma, multiple myeloma, metastatic melanoma, and non-small cell lung cancer.[7] Therefore, although our study used three tumor models (i.e., prostate cancer, melanoma, and AML) to establish the proof of concept, we speculate that anti-MICA/B antibodies that inhibit the shedding and with an Fc optimized for ADCC provide a therapeutic opportunity that is broadly applicable to different cancer types.

In summary, we developed a next-generation anti-MICA/B antibody that inhibits the shedding, by introducing GAALIE mutations in the Fc to increase the binding affinity to Fcγ-activating receptors. As a consequence, this Fc-optimized antibody more potently elicited NK cell-driven immunity and, in general, displayed therapeutic activity in preclinical models of prostate cancer, melanoma, and leukemia. Therefore, GAALIE mutations represent an effective means to enhance the therapeutic efficacy of anti-MICA/B antibodies for cancer.

Limitations of the study

A limitation in this study was that the binding affinity of human IgG subclasses to human Fcγ-activating receptors follows a gradient-like pattern that can be ranked as IgG1-GAALIE > IgG1 > IgG3 > IgG2 > IgG4. Therefore, 7C6-GAALIE and 7C6-hIgG1 are not the two extremes in the affinity spectrum. Another limitation in this study was that the *MICA* and *MICB* genes are highly polymorphic, but the *in vivo* models included only MICA allele 009 and MICB allele 005 in the engineered cell lines.

RESOURCE AVAILABILITY

Lead contact

Further information and requests for resources and reagents should be directed to and will be fulfilled by the lead contact, Lucas Ferrari de Andrade (lucas.ferrarideandrade@mssm.edu).

Materials availability

7C6-GAALIE and NPK-MICB are the unique/stable reagents generated in this study and are available from the lead contact with a completed materials transfer agreement.

Data and code availability

- Data: The RNA sequencing data of human NK cells that were co-cultured with DU-145, which were pre-treated with 7C6 (hIgG1 or GAALIE) or isotype, are available in Gene Expression Omnibus, GEO: GSE317314.

- Code: This paper does not report custom computer code.
- General statement: Any additional information required to reanalyze the data reported in this work paper is available from the lead contact upon request.

ACKNOWLEDGMENTS

We thank Kai Wucherpfennig (Dana-Farber Cancer Institute), Jeffrey Ravetch (The Rockefeller University), and Thomas Pabst (University of Bern) for kindly providing the previous versions of 7C6 (wild-type hIgG1, DANA, and mIgG2a), hFcR mice, and AML cell lines, respectively. We also thank Xiaoyu Song (Mount Sinai) for statistical support during conceptualization phases. This study was supported in part by the Tisch Cancer Institute Hematological Malignancies Tissue Bank and the Biorepository and Pathology Core at the Icahn School of Medicine at Mount Sinai. We thank the following funding sources to L.F.d.A.: National Institutes of Health grant R37CA269982, Blood Cancer United grant 6647-23, Department of Defense grants CA210940 and BC241026P1, Cancer Research Institute grant CRI3483, American Lung Association grant LCD-1022356, American Cancer Society grant RSG-24-1188643-01-IBCD, Elsa U. Pardee Foundation, and TCI Developmental Funds Award-National Cancer Institute grant P30CA196521.

AUTHOR CONTRIBUTIONS

Conceptualization, R.P., S.M., and L.F.d.A.; investigation and analyses, R.P., S.M., X.Z., B.T.d.S.B., S.Q., L.R.d.L.B., B.M., J.M.A., J.M., and L.F.d.A.; funding acquisition, J.M. and L.F.d.A.; project administration, L.F.d.A.; supervision, L.F.d.A.; writing – original draft, R.P. and L.F.d.A.; writing – review and editing, R.P., S.M., X.Z., B.T.d.S.B., S.Q., L.R.d.L.B., B.M., J.M.A., J.M., and L.F.d.A.

DECLARATION OF INTERESTS

L.F.d.A. is named inventor in patents that claim 7C6; they were filed through the Dana-Farber Cancer Institute, and L.F.d.A. received royalty payments in relation to this antibody. L.F.d.A. has also served in a translational advisory board for Cullinan MICA.

STAR★METHODS

Detailed methods are provided in the online version of this paper and include the following:

- KEY RESOURCES TABLE
- EXPERIMENTAL MODEL AND STUDY PARTICIPANT DETAILS
  - Cell lines
  - Animal models
  - Human samples
- METHOD DETAILS
  - Antibody production
  - Enzyme-linked immunosorbent assays (ELISA)
  - MICA/B shedding assays with cell lines
  - *MICA* and *MICB* gene expression analysis
  - Wound healing (scratch) assay
  - Transwell invasion assay
  - MICA/B shedding assays with AML patient samples
  - Human NK cell effector function assays
  - RNA-sequencing of human NK cells
  - Incucyte live-cell imaging assays
  - Phagocytosis assays
  - Characterization of parental NPK cells
  - Mouse prostate cancer metastasis model
  - Mouse melanoma metastasis model
  - Mouse AML model
  - Human AML *in vivo* model
- QUANTIFICATION AND STATISTICAL ANALYSIS

SUPPLEMENTAL INFORMATION

Supplemental information can be found online at https://doi.org/10.1016/j.xcrm.2026.102753.

Received: September 11, 2025
Revised: January 27, 2026
Accepted: March 18, 2026
Published: April 17, 2026

REFERENCES

1. Raulet, D.H., Gasser, S., Gowen, B.G., Deng, W., and Jung, H. (2013). Regulation of ligands for the NKG2D activating receptor. Annu. Rev. Immunol. *31*, 413–441. https://doi.org/10.1146/annurev-immunol-032712-095951.
2. Wu, J., Song, Y., Bakker, A.B., Bauer, S., Spies, T., Lanier, L.L., and Phillips, J.H. (1999). An activating immunoreceptor complex formed by NKG2D and DAP10. Science *285*, 730–732. https://doi.org/10.1126/science.285.5428.730.
3. Bauer, S., Groh, V., Wu, J., Steinle, A., Phillips, J.H., Lanier, L.L., and Spies, T. (1999). Activation of NK cells and T cells by NKG2D, a receptor for stress-inducible MICA. Science *285*, 727–729. https://doi.org/10.1126/science.285.5428.727.
4. Groh, V., Rhinehart, R., Randolph-Habecker, J., Topp, M.S., Riddell, S.R., and Spies, T. (2001). Costimulation of CD8alphabeta T cells by NKG2D via engagement by MIC induced on virus-infected cells. Nat. Immunol. *2*, 255–260. https://doi.org/10.1038/85321.
5. Groh, V., Wu, J., Yee, C., and Spies, T. (2002). Tumour-derived soluble MIC ligands impair expression of NKG2D and T-cell activation. Nature *419*, 734–738. https://doi.org/10.1038/nature01112.
6. Wang, X., Lundgren, A.D., Singh, P., Goodlett, D.R., Plymate, S.R., and Wu, J.D. (2009). An six-amino acid motif in the α3 domain of MICA is the cancer therapeutic target to inhibit shedding. Biochem. Biophys. Res. Commun. *387*, 476–481.
7. Xing, S., and Ferrari de Andrade, L. (2020). NKG2D and MICA/B shedding: a 'tag game' between NK cells and malignant cells. Clin. Transl. Immunol. *9*, e1230. https://doi.org/10.1002/cti2.1230.
8. Ferrari de Andrade, L., Tay, R.E., Pan, D., Luoma, A.M., Ito, Y., Badrinath, S., Tsoucas, D., Franz, B., May, K.F., Jr., Harvey, C.J., et al. (2018). Antibody-mediated inhibition of MICA and MICB shedding promotes NK cell-driven tumor immunity. Science *359*, 1537–1542. https://doi.org/10.1126/science.aao0505.
9. Whalen, K.A., Henry, C.C., Mehta, N.K., Rakhra, K., Yalcin, S., Meetze, K., Gibson, N.W., Baeuerle, P.A., and Michaelson, J.S. (2025). CLN-619, a MICA/B monoclonal antibody that promotes innate immune cell-mediated antitumor activity. J. Immunother. Cancer *13*, e008987. https://doi.org/10.1136/jitc-2024-008987.
10. Lombana, T.N., Matsumoto, M.L., Berkley, A.M., Toy, E., Cook, R., Gan, Y., Du, C., Schnier, P., Sandoval, W., Ye, Z., et al. (2019). High-resolution glycosylation site-engineering method identifies MICA epitope critical for shedding inhibition activity of anti-MICA antibodies. mAbs *11*, 75–93. https://doi.org/10.1080/19420862.2018.1532767.
11. Huang, R.Y.C., Kuhne, M., Deshpande, S., Rangan, V., Srinivasan, M., Wang, Y., and Chen, G. (2020). Mapping binding epitopes of monoclonal antibodies targeting major histocompatibility complex class I chain-related A (MICA) with hydrogen/deuterium exchange and electron-transfer dissociation mass spectrometry. Anal. Bioanal. Chem. *412*, 1693–1700. https://doi.org/10.1007/s00216-020-02409-x.
12. Ignacio Melero Bermejo, J.S.W., Gutierrez, M., Hamilton, E.P., Spira, A.I., Rasco, D.W., John, D., Powderly, I.I., Millward, M., Frentzas, S., Stec, R., Sharma, M., et al. (2024). Cln-619 (anti-MICA/B antibody) alone and in combination with pembrolizumab (P) for advanced solid tumors: Updated results of a Ph1 study. J. Clin. Oncol. *42*, suppl.2588.

13. Bournazos, S., Wang, T.T., Dahan, R., Maamary, J., and Ravetch, J.V. (2017). Signaling by Antibodies: Recent Progress. Annu. Rev. Immunol. *35*, 285–311. https://doi.org/10.1146/annurev-immunol-051116-052433.

14. Bibeau, F., Lopez-Crapez, E., Di Fiore, F., Thezenas, S., Ychou, M., Blanchard, F., Lamy, A., Penault-Llorca, F., Frébourg, T., Michel, P., et al. (2009). Impact of FcgammaRIIa-FcgammaRIIIa polymorphisms and KRAS mutations on the clinical outcome of patients with metastatic colorectal cancer treated with cetuximab plus irinotecan. J. Clin. Oncol. *27*, 1122–1129. https://doi.org/10.1200/JCO.2008.18.0463.

15. Cartron, G., Dacheux, L., Salles, G., Solal-Celigny, P., Bardos, P., Colombat, P., and Watier, H. (2002). Therapeutic activity of humanized anti-CD20 monoclonal antibody and polymorphism in IgG Fc receptor FcgammaRIIIa gene. Blood *99*, 754–758. https://doi.org/10.1182/blood.v99.3.754.

16. Gavin, P.G., Song, N., Kim, S.R., Lipchik, C., Johnson, N.L., Bandos, H., Finnigan, M., Rastogi, P., Fehrenbacher, L., Mamounas, E.P., et al. (2017). Association of Polymorphisms in FCGR2A and FCGR3A With Degree of Trastuzumab Benefit in the Adjuvant Treatment of ERBB2/HER2-Positive Breast Cancer: Analysis of the NSABP B-31 Trial. JAMA Oncol. *3*, 335–341. https://doi.org/10.1001/jamaoncol.2016.4884.

17. Yamin, R., Jones, A.T., Hoffmann, H.H., Schäfer, A., Kao, K.S., Francis, R.L., Sheahan, T.P., Baric, R.S., Rice, C.M., Ravetch, J.V., and Bournazos, S. (2021). Fc-engineered antibody therapeutics with improved anti-SARS-CoV-2 efficacy. Nature *599*, 465–470. https://doi.org/10.1038/s41586-021-04017-w.

18. Bournazos, S., Corti, D., Virgin, H.W., and Ravetch, J.V. (2020). Fc-optimized antibodies elicit CD8 immunity to viral respiratory infection. Nature *588*, 485–490. https://doi.org/10.1038/s41586-020-2838-z.

19. Osorio, J.C., Smith, P., Knorr, D.A., and Ravetch, J.V. (2023). The antitumor activities of anti-CD47 antibodies require Fc-FcgammaR interactions. Cancer Cell *41*, 2051. https://doi.org/10.1016/j.ccell.2023.10.007.

20. Alves da Silva, P.H., Xing, S., Kotini, A.G., Papapetrou, E.P., Song, X., Wucherpfennig, K.W., Mascarenhas, J., and Ferrari de Andrade, L. (2022). MICA/B antibody induces macrophage-mediated immunity against acute myeloid leukemia. Blood *139*, 205–216. https://doi.org/10.1182/blood.2021011619.

21. Wu, J.D., Higgins, L.M., Steinle, A., Cosman, D., Haugk, K., and Plymate, S.R. (2004). Prevalent expression of the immunostimulatory MHC class I chain-related molecule is counteracted by shedding in prostate cancer. J. Clin. Investig. *114*, 560–568. https://doi.org/10.1172/JCI22206.

22. López-Soto, A., Gonzalez, S., Smyth, M.J., and Galluzzi, L. (2017). Control of Metastasis by NK Cells. Cancer Cell *32*, 135–154. https://doi.org/10.1016/j.ccell.2017.06.009.

23. Wu, B., Zhang, B., Li, B., Wu, H., and Jiang, M. (2024). Cold and hot tumors: from molecular mechanisms to targeted therapy. Signal Transduct. Target. Ther. *9*, 274. https://doi.org/10.1038/s41392-024-01979-x.

24. Arriaga, J.M., Panja, S., Alshalalfa, M., Zhao, J., Zou, M., Giacobbe, A., Madubata, C.J., Kim, J.Y., Rodriguez, A., Coleman, I., et al. (2020). A MYC and RAS co-activation signature in localized prostate cancer drives bone metastasis and castration resistance. Nat. Cancer *1*, 1082–1096. https://doi.org/10.1038/s43018-020-00125-0.

25. Klussmeier, A., Massalski, C., Putke, K., Schäfer, G., Sauter, J., Schefzyk, D., Pruschke, J., Hofmann, J., Fürst, D., Carapito, R., et al. (2020). High-Throughput MICA/B Genotyping of Over Two Million Samples: Workflow and Allele Frequencies. Front. Immunol. *11*, 314. https://doi.org/10.3389/fimmu.2020.00314.

26. Smith, P., DiLillo, D.J., Bournazos, S., Li, F., and Ravetch, J.V. (2012). Mouse model recapitulating human Fcgamma receptor structural and functional diversity. Proc. Natl. Acad. Sci. USA *109*, 6181–6186. https://doi.org/10.1073/pnas.1203954109.

27. Katano, I., Ito, R., Kawai, K., and Takahashi, T. (2020). Improved Detection of in vivo Human NK Cell-Mediated Antibody-Dependent Cellular Cytotoxicity Using a Novel NOG-FcgammaR-Deficient Human IL-15 Transgenic Mouse. Front. Immunol. *11*, 532684. https://doi.org/10.3389/fimmu.2020.532684.

28. Mori, K., Iida, S., Yamane-Ohnuki, N., Kanda, Y., Kuni-Kamochi, R., Nakano, R., Imai-Nishiya, H., Okazaki, A., Shinkawa, T., Natsume, A., et al. (2007). Non-fucosylated therapeutic antibodies: the next generation of therapeutic antibodies. Cytotechnology *55*, 109–114. https://doi.org/10.1007/s10616-007-9103-2.

29. Allegretti, Y.L., Bondar, C., Guzman, L., Cueto Rua, E., Chopita, N., Fuertes, M., Zwirner, N.W., and Chirdo, F.G. (2013). Broad MICA/B expression in the small bowel mucosa: a link between cellular stress and celiac disease. PLoS One *8*, e73658. https://doi.org/10.1371/journal.pone.0073658.

30. Sharkawy, R.E., Bayoumi, A., Metwally, M., Mangia, A., Berg, T., Romero-Gomez, M., Abate, M.L., Irving, W.L., Sheridan, D., Dore, G.J., et al. (2019). A variant in the MICA gene is associated with liver fibrosis progression in chronic hepatitis C through TGF-beta1 dependent mechanisms. Sci. Rep. *9*, 1439. https://doi.org/10.1038/s41598-018-35736-2.

31. Lazar, G.A., Dang, W., Karki, S., Vafa, O., Peng, J.S., Hyun, L., Chan, C., Chung, H.S., Eivazi, A., Yoder, S.C., et al. (2006). Engineered antibody Fc variants with enhanced effector function. Proc. Natl. Acad. Sci. USA *103*, 4005–4010. https://doi.org/10.1073/pnas.0508123103.

32. Casneuf, T., Xu, X.S., Adams, H.C., Axel, A.E., Chiu, C., Khan, I., Ahmadi, T., Yan, X., Lonial, S., Plesner, T., et al. (2017). Effects of daratumumab on natural killer cells and impact on clinical outcomes in relapsed or refractory multiple myeloma. Blood Adv. *1*, 2105–2114. https://doi.org/10.1182/bloodadvances.2017006866.

33. Wang, Y., Zhang, Y., Hughes, T., Zhang, J., Caligiuri, M.A., Benson, D.M., and Yu, J. (2018). Fratricide of NK Cells in Daratumumab Therapy for Multiple Myeloma Overcome by Ex Vivo-Expanded Autologous NK Cells. Clin. Cancer Res. *24*, 4006–4017. https://doi.org/10.1158/1078-0432.CCR-17-3117.

34. Delconte, R.B., Owyong, M., Santosa, E.K., Srpan, K., Sheppard, S., McGuire, T.J., Abbasi, A., Diaz-Salazar, C., Chun, J., Rogatsky, I., et al. (2024). Fasting reshapes tissue-specific niches to improve NK cell-mediated anti-tumor immunity. Immunity *57*, 1923–1938.e7. https://doi.org/10.1016/j.immuni.2024.05.021.

35. Salih, H.R., Antropius, H., Gieseke, F., Lutz, S.Z., Kanz, L., Rammensee, H.G., and Steinle, A. (2003). Functional expression and release of ligands for the activating immunoreceptor NKG2D in leukemia. Blood *102*, 1389–1396. https://doi.org/10.1182/blood-2003-01-0019.

36. Mastaglio, S., Wong, E., Perera, T., Ripley, J., Blombery, P., Smyth, M.J., Koldej, R., and Ritchie, D. (2018). Natural killer receptor ligand expression on acute myeloid leukemia impacts survival and relapse after chemotherapy. Blood Adv. *2*, 335–346. https://doi.org/10.1182/bloodadvances.2017015230.

STAR★METHODS

KEY RESOURCES TABLE

REAGENT or RESOURCE	SOURCE	IDENTIFIER
**Antibodies**		
7C6-GAALIE	This paper	N/A
7C6-hIgG1	Ferrari de Andrade et al.[8]	N/A
7C6-DANA	Ferrari de Andrade et al.[8]	N/A
Anti-human NKG2D blocking antibody	Biolegend	320802; RRID:AB_492956
Anti-mouse NKG2D blocking antibody	Bio X Cell	BE0111; RRID:AB_10950118
Anti-mouse NK1.1 antibody	Bio X Cell and IchorBio	BE0036; RRID:AB_1107737; ICH1128; RRID:AB_2921496
Anti-human MICA/B antibody clone 6D4	Biolegend	320906; RRID:AB_493193
**Bacterial and virus strains**		
pHAGE-EF1a-MICB*005-IRES-ZsGreen	Addgene	Catalog number 114008
**Biological samples**		
Leukemic specimens from patients	Hematological Malignancies Tissue Bank (Mount Sinai)	N/A
Buffy coats	New York Blood Center	N/A
**Chemicals, peptides, and recombinant proteins**		
Recombinant human MICA protein	ACROBiosystems	MIAH5221100UG
Recombinant human MICB protein	ACROBiosystems	MIBH52H3100UG
Recombinant human CD16a protein	Sino Biological and Biolegend	10389H27HB20 and 777102
Recombinant human CD32a protein	Biolegend	781402
Recombinant human CD64 protein	Biolegend	790002
Recombinant human CD32b protein	Biolegend	781702
**Critical commercial assays**		
Human MICA duoset ELISA	R&D Systems	DY1300
Human MICB duoset ELISA	R&D Systems	DY1599
**Deposited data**		
NK cell RNA-sequencing data	This paper	Gene Expression Omnibus, GEO: GSE317314
**Experimental models: Cell lines**		
C1498-MICB	Alves da Silva et al.[20]	N/A
B16F10-MICA	Ferrari de Andrade et al.[8]	N/A
DU145	ATCC	HTB-81
HCT-116	ATCC	CCL-247
MDA-MB-468	ATCC	HTB-132
MOLM13	Thomas Pabst (University of Bern)	N/A
SKM1	Thomas Pabst (University of Bern)	N/A
OCI-AML-3	Thomas Pabst (University of Bern)	N/A
B16F10-MICB	Ferrari de Andrade et al.[8]	N/A
NPK	Arriaga et at.[24]	N/A
NPK-MICB	This paper.	N/A
**Experimental models: Organisms/strains**		
Fc gamma receptor-humanized mice	Jeffrey Ravetch (The Rockefeller University) and Smith et al.[26]	N/A
FcResolv® hIL-15 NOG mice	Taconic	19220-M

(Continued on next page)

Continued		
REAGENT or RESOURCE	SOURCE	IDENTIFIER
Oligonucleotides		
MICA forward primer: 5′-GGCA TCTTCCCTTTTGCACC-3′	This paper.	N/A
MICA reverse primer: 5′-CCAC GTTTTGGGAGAGGAAGA-3′	This paper.	N/A
MICB forward primer: 5′-GTCG CCTTCCCTTTTGCACC-3′	This paper.	N/A
MICB reverse primer: 5′-CTGG GGCACTGTCGATTCTT-3′	This paper.	N/A
GAPDH forward primer: 5′-ATG TTGCAACCGGGAAGGAA-3′	This paper.	N/A
GAPDH reverse primer: 5′-GCA GGAGGCATTGCTGATGA-3′	This paper.	N/A
Software and algorithms		
ImageJ	https://imagej.net/ij/	N/A
Prism	GraphPad	N/A
FlowJo	Waters Biosciences	N/A

EXPERIMENTAL MODEL AND STUDY PARTICIPANT DETAILS

Cell lines

MOLM13, OCI-AML-3, and SKM-1 were kind gifts from Thomas Pabst (University of Bern). C1498-MICB was described.[20] DU-145, HCT-116, MDA-MB-468 were from the American Type Tissue Culture Collection. To establish the syngeneic NPK cell line, *NPK* mice (*Nkx3.1*$^{CreERT2/+}$; *Pten*$^{fl/fl}$; *Kras*$^{LSL-G12D/+}$, *R26*$^{CAG-LSL-EYFP/+}$) were backcrossed with wild-type C57BL/6J mice for over six generations and confirmed inbred into a C57BL/6J background with MiniMUGA SNP genotyping array (Transnetyx). The *NPK* mice were described.[24] A cell line was then established from a spontaneous bone metastasis of an inbred *NPK* mouse, which occurred in the spine seven months after tumor induction by tamoxifen treatment, as described.[24] These cells expressed enhanced yellow fluorescent protein, confirming that they originated from *Nkx3-1*$^{+}$ prostate cells. The human cell lines were cultured in RPMI-1640 whereas the mouse cell lines were cultured in DMEM, and both medium types were complemented with 10% fetal bovine serum (FBS), Glutamax, HEPES, and penicillin/streptomycin. MOLM13 and NPK were engineered to express human MICB by transduction with a lentiviral vector (Addgene 114008) that was reported previously and sorted in a BD Aria or Aurora CS cell sorter (Cytek Biosciences).[8] All cell lines were routinely tested negative for mycoplasma contamination with MycoAlert Mycoplasma Detection Kit (Lonza). Cells were cultured in a humidified atmosphere at 37 $^{\circ}$C and with 5% CO_2.

Animal models

Fc gamma receptor-humanized mice, abbreviated as “hFcR mice,” and FcResolv hIL-15 NOG mice were maintained in the Center for Comparative Medicine and Surgery, which is a rodent barrier facility with water and food *ad libitum* at Mount Sinai (New York). All mice used in this study were age matched and adult (more than six weeks old). The mouse prostate cancer model and mouse and human AML models used male mice only. The mouse melanoma models used female mice only. The procedures involving mice in this study were approved by the Institutional Animal Care and Use Committee, under protocol PROTO201900599.

Human samples

Peripheral blood mononuclear cells (PBMC) from untreated patients with *de novo* AML were provided by the Hematological Malignancies Tissue Bank (Mount Sinai) with approval by the Institutional Review Board (study number 22–00852); research was conducted in accordance with the Declaration of Helsinki and the relevant guidelines and regulations, and, given the retrospective nature of this research, informed consent was not required by local regulatory authorities. De-identified buffy coat samples of volunteer donors were purchased from the New York Blood Center (New York), which obtained written informed consent for all donors. Human subject personal information was not used in this study. The use of AML patient-derived PBMC and buffy coats from volunteer donors in this study was also approved by the Institutional Review Board under protocol number STUDY-22-00038.

METHOD DETAILS

Antibody production

7C6-hIgG1, 7C6-DANA (hIgG1), and 7C6-mIgG2a were described.[8] GAALIE mutations were introduced in the Fc sequence of UCOE vector encoding 7C6-hIgG1 light and heavy chains connected by 2A peptide. CHO and 293T cells were transfected with 7C6-GAALIE-encoding plasmid through TransIT-293 (Mirus Bio) followed by puromycin selection. Supernatants were filtered, ran through protein G column, and eluted in glycine buffer pH 2.5. Endotoxin levels (Chromogenic Endotoxin Quant Kit, Thermo Fisher) were <0.01 units/mL, the lower-end detection limit. The hIgG1 (BE0297) and mIgG2a (C1184) isotype control, control IgG1 (MOPC21), anti-NK1.1 (PK136), and anti-NKG2D (HMG2D) were from Bio X Cell and IchorBio. Antibodies were in PBS pH 7.4 at 4°C for short-term or −80°C for long-term storage.

Enzyme-linked immunosorbent assays (ELISA)

In Fc receptor assays, 0.1 μg of 7C6-GAALIE, 7C6-hIgG1, and 7C6-DANA were immobilized in multi-well plates followed by blockade with bovine serum albumin (BSA), which was removed in the next steps. Biotinylated Fcγ receptor I, IIa, IIb, IIIa, and IV proteins (ACROBiosystems, Sino Biological, and Biolegend) were added in concentration ranges indicated in Figure 1A. In MICA ELISA, 0.1 μg MICA protein (ACROBiosystems) was immobilized in multi-well plates followed by BSA incubation, which remained to be used in the subsequent steps. 7C6-GAALIE, 7C6-hIgG1, or isotype were added in the concentration ranges shown in Figure 1B; biotinylated anti-human IgG secondary antibody (Biolegend) was added at 1 μg/mL. Peroxidase-labeled streptavidin (Jackson ImmunoResearch Laboratories) was added, which was followed by TMB substrate (Biolegend) and sulfuric acid. Washing steps were done with TBS-T buffer (Boston Bioproducts). 450 nm absorbance was read in PerkinElmer EnVision reader.

MICA/B shedding assays with cell lines

The cell lines were cultured for 24 h in 96-well plates. The antibodies, antibody doses, and romidepsin doses are indicated in the figure graphs/legends of this study. After 24 h, the cells had Fc receptors blocked with mouse or human TruStain FcX and were stained with phycoerythrin-conjugated MICA/B antibody (6D4) and Zombie Yellow (Biolegend) or 7-AAD (Biolegend). Cells were acquired in BD LSRII Fortessa and analyzed by FlowJo. Soluble MICA and MICB molecules were shed in supernatants by Human MICA or MICB DuoSet ELISAs (R&D Systems).

MICA and *MICB* gene expression analysis

DU-145 was treated for 24 h with romidepsin at concentrations of 0, 0.1, 1.25, and 5 nmol/L. Total RNA was extracted from DU-145 cells using the mirVana miRNA Isolation Kit (Ambion), which includes genomic DNA removal by column. Complementary DNA was synthesized from the total RNA using Verso cDNA Synthesis Kit (Thermo Fisher Scientific), and PCR was performed using DreamTa Green Master Mix (Thermo Fisher Scientific). PCR products were separated by agarose gel electrophoresis and visualized, and band intensities were quantified using ImageJ software. Gene expression levels were normalized to the housekeeping gene *GAPDH*. The following primers were used: MICA (forward: 5′-GGCATCTTCCCTTTTGCACC-3’; reverse: 5′-CCACGTTTTGGGAGAGGAAGA-3′), MICB (forward: 5′-GTCGCCTTCCCTTTTGCACC-3’; reverse: 5′-CTGGGGCACTGTCGATTCTT-3′), and GAPDH (forward: 5′-ATGT TGCAACCGGGAAGGAA-3’; reverse: 5′-GCAGGAGGCATTGCTGATGA-3′).

Wound healing (scratch) assay

DU-145and NPK-MICB cells were seeded into 6-well plates at a density of 1×10^6 cells per well and cultured until confluence. A linear wound was generated in each well using 1 mL pipette tip. Cells were gently washed with PBS to remove detached cells and debris, and serum-free culture medium was added. Cells were then treated with 10 μg/mL of hIgG1 isotype, 7C6-hIgG1, or 7C6-GAALIE. Phase-contrast images were acquired at 0, 4, 24, and 48 h after wound generation using an EVOS XL Core microscope (Thermo Fisher Scientific). Wound closure was quantified using ImageJ software, and cell migration was expressed as the percentage of wound closure relative to time zero.

Transwell invasion assay

DU-145 and NPK-MICB cells were used to assess invasion using transwell chambers with 8-μm pore size (Greiner Bio-One). Inserts were coated with matrigel matrix diluted 1:2 in serum-free culture medium. 1×10^4 cells per insert, resuspended in serum-free medium, were seeded into the upper chamber. The lower chamber was filled with culture medium containing 10% FBS as a chemoattractant. Cells were treated with 10 μg/mL hIgG1 isotype hIgG1, 7C6-hIgG1, or 7C6-GAALIE and incubated at 37°C in a humidified 5% CO_2 for 48 h. After incubation, non-invading cells on the upper surface of the membrane were gently removed. The cells on the lower surface were fixed with 4% formaldehyde in PBS and stained with 0.2% crystal violet in methanol. Invaded cells were counted under an optical microscope.

MICA/B shedding assays with AML patient samples

Primary PBMC from patients were treated for 24 h with 7C6-DANA at 10 μg/mL plus romidepsin that was titrated from 1,000 to 0.3 nmol/L, and cultured in StemSpan media (StemCell Technologies) complemented with 150 ng/mL stem cell factor, 100 ng/mL

thrombopoietin, 100 ng/mL Fms-related tyrosine kinase 3 ligand, and 50 ng/mL interleukin 3, and in a humidified atmosphere at 37°C and with 5% CO_2. MICA/B expressions were analyzed by flow cytometry, as above.

Human NK cell effector function assays

NK cells were from de-identified buffy coat samples of volunteer donors (New York Blood Center), isolated by negative selection through the MojoSort Human NK Cell Isolation Kit (Biolegend), and cultured for up to three weeks in G-rex plates (Wilson Wolf) and RPMI-1640 complemented as above plus 5% human AB serum, 20 ng/mL human interleukin 15, and 1,000 units/mL human interleukin-2. The target cells (MOLM13 and DU-145) were treated for 24 h with antibody and, when applicable, romidepsin doses that are indicated in the figures. MOLM13 was also labeled with calcein AM (Thermo Fisher). Specifically for the CD107a externalization assay, NK and the target cells were co-cultured in 1 : 1 ratio for one hour prior to administration of monensin, which was followed by another three hours, whereas CD107a (H4A3, Biolegend) and NKG2D (1D11, Biolegend) antibodies were added at the start of co-culture. Co-cultures were in V-bottom 96-well plates for 4 h total, which was followed by analyses in BD LSRII Fortessa after staining with antibodies (human CD45, CD33, and CD56, clones P67.6, 2D1, and HCD56, respectively) and 7-AAD (Biolegend). For interferon-γ production assay, NK and MOLM13 cells were prepared as above but without calcein labeling and co-cultured for six hours in 1 : 1 ratio and with Brefeldin A, followed by staining with CD33, CD45, and CD56 antibodies, fixation, permeabilization, and staining with human interferon-γ antibody (clone 4S.B3) (Biolegend).

RNA-sequencing of human NK cells

DU-145 cells were seeded into 6-well plates at a density of 2×10^6 cells per well and incubated overnight with 10 μg/mL of hIgG1 isotype control, 7C6-hIgG1, or 7C6-GAALIE. The following day, human NK cells that were isolated as described above were added to the cultures at an effector to target ratio of 1 (2×10^6 NK cells per well) and co-cultured for 4 h at 37°C and 5% CO_2. After co-culture, NK cells in suspension were carefully collected without disturbing the adherence tumor cells, and total mRNA was isolated using the RNeasy Plus Mini Kit (Qiagen), which included on-column genomic DNA removal, according to the manufacturer's instructions. Library preparation, sequencing, and data analysis were performed at Genewiz (South Plainfield, New Jersey).

Incucyte live-cell imaging assays

DU-145 cells were seeded in 96-well plates at a density of 1×10^4 cells per well and incubated with 10 μg/mL hIgG1 isotype control, 7C6-hIgG1, or 7C6-GAALIE. After antibody exposure for 24 h, tumor cells were labeled with 15 μM Calcein AM (Invitrogen) following the manufacturer's instructions. Calcein-labeled DU-145 cells were then combined with human NK cells, prepared as described above, at an effector target ratio of 5 : 1. Co-cultures were maintained under standard incubation conditions, and dynamic interactions were monitored using the Incucyte live-cell imaging system, with phase-contrast and fluorescence images captured every 30 min for a total duration of 23 h.

Phagocytosis assays

The sources of human monocyte-derived macrophages and murine bone marrow-derived macrophages were de-identified buffy coats of volunteer donors (New York Blood Center) and hFcR mice. Human monocytes were differentiated to macrophages by granulocyte macrophage colony stimulating factor for ten days. Bone marrow cells from hFcR mice were induced to differentiate via macrophage colony stimulating factor for two weeks. MOLM13, SKM-1, and OCI-AML3 were treated with romidepsin plus antibodies, and MOLM13-MICB and C1498-MICB were treated with antibodies as above; they were labeled with carboxyfluorescein succinimidyl ester (CFSE), and co-cultured for two hours with macrophages. Subsequently, macrophages were detached using EDTA and stained with mouse F4/80 or human CD206 antibodies, followed by analyses in BD LSRII Fortessa and Flowjo. Since macrophages acquire CFSE fluorescence upon phagocytosis of target cells, we analyzed the percentages of $CFSE^+$ macrophages.

Characterization of parental NPK cells

NPK cells were stained with anti-mouse Rae1 pan-specific (clone 186107), Rae1-δ (Charlotte d1.23), or MULT-1 (clone 237104) antibodies or an isotype control (Biolegend) and 7-AAD. For the analysis of major histocompatibility class I expression, NPK cells were treated for 24 h with 10 ng/mL recombinant mouse interferon-γ protein or PBS, which was followed by the labeling with anti-mouse H-2Db (KH95), H-2Kb (AF6-88.5), and Qa-1 (6A8.6F10.1A6) antibodies and 7-AAD. Cells were analyzed by flow cytometry.

Mouse prostate cancer metastasis model

Two mouse strains were used: wild-type C57BL/6 (JAX mice) and Fcγ receptor-humanized (hFcR) mice. The latter strain was described and a kind gift from Dr. Jeffrey Ravetch (The Rockefeller University).[26] The mice were male and 6–8 weeks old. For the analyses of metastases in the lungs of wild-type mice, NPK or NPK-MICB cells were intravenously inoculated at a dose of 5×10^3 cells via the tail vein. All antibodies were administered by intraperitoneal injection. Control IgG and anti-NKG2D and NK1.1 antibodies were given at a dose of 0.1 mg per mouse and on days −1 and 0 and once per week. 7C6-mIgG2a or isotype controls were given at a dose of 0.2 mg per mouse and on days 1 and 2, which was followed by a dose of 0.1 mg per mouse once per week. For experiments evaluating 7C6-hIgG1 and 7C6-GAALIE, hFcR mice were intravenously inoculated with 1×10^5 NPK-MICB cells to ensure robust tumor establishment. Treatments with isotype control, 7C6-hIgG1, and 7C6-GAALIE were as above. On day 15,

CellPress
OPEN ACCESS

mice were euthanized by CO_2 inhalation, and metastases were analyzed either by histopathology or a stereomicroscopy technique whereby the lungs were stained with Indian ink at 10% to enable the identification of metastatic foci. Serum samples were collected and used to quantify soluble MICB levels using a commercial ELISA kit (R&D Systems, DY1599), with optical density measured at 450 nm. Serum toxicity biomarkers were assessed using enzymatic assays for creatinine (Mouse Creatinine Assay, Crystal Chem, Elk Grove Village, IL, USA) and aspartate aminotransferase (AST; Darmstadt, Germany), as well as an immunoenzymatic assay for alanine aminotransferase (ALT/GPT; TSZ Scientific, USA), according to the manufacturers' instructions. Separate cohorts of mice were euthanized when they developed any disease signs (i.e., moribund state, curved posture, limb paralysis, loss of body weight $\geq$20%, and body condition score $\geq$2), and mouse organs were collected for histopathology.

Mouse melanoma metastasis model

The B16F10-MICB and B16F10-MICA cells were described.[8] Fcγ receptor-humanized (hFcR) mice (female, 6–8 weeks old) were intravenously injected via the tail vein with 5×10^5 B16F10-MICA or MICB cells. Treatments with 7C6-hIgG1, 7C6-GAALIE, or isotype were administered by intraperitoneal injections, at a dose of 0.2 mg per mouse on days 1 and 2 after tumor cell injection, followed by 0.1 mg per mouse once per week thereafter. Mouse weight was monitored over the entire course of the model. After 15 days, mice were euthanized with CO_2, and lung metastases were quantified by stereomicroscopy.

Mouse AML model

hFcR mice were male, 6–8 weeks old and inoculated intravenously with C1498-MICB at a dose of 3 x 10^6. Treatments with 0.2 mg 7C6-hIgG1, 7C6-GAALIE, or hIgG1 isotype were by intraperitoneal injections on days 5 and 6 and once per week. Analyses of leukemia after CO_2 euthanasia were performed on day 21. Blood was collected by cardiac puncture and placed in tubes with 10 mmol/L EDTA in PBS, whereas femurs had the epiphyses cut out and bone marrow flushed with PBS. Red blood cells were lysed with ACK buffer, and murine and human Fc receptors were blocked with Trustain FcX, which was followed by staining with anti-human CD16a/b (clone 3G8) antibody and anti-mouse NKG2D (CX5), NK1.1 (PK136), CD49b (HMa2), CD11b (M1/70), F4/80 (BM8), CD3 (17A2), B220 (RA3-6B2), and Ly6G (1A8) antibodies and Zombie Yellow.

Human AML *in vivo* model

FcResolv hIL-15 NOG mice (male and 6–8 weeks old, Taconic) were inoculated intravenously through the tail vein with 1 x 10^6 human NK cells isolated from volunteer donors' buffy coats (New York Blood Center). One week later, the mice were inoculated intravenously with 2 x 10^6 MOLM13-MICB. Treatments with 0.2 mg of 7C6-GAALIE, 7C6-hIgG1, or isotype were by intraperitoneal injections on days 5, 6, and 11 after leukemia inoculation. Euthanasia by CO_2 on day 17 was followed by analyses of NK and leukemia cells in the blood and femoral bone marrow, as above. Samples were stained with mouse CD45.1 (A20), human CD45 (2D1), CD33 (P67.6), CD56 (HCD56), CD16a/b (3G8), and NKG2D (1D11) and Zombie Yellow (Biolegend). Cells were counted in a hemocytometer and analyzed in BD LSRII Fortessa and FlowJo.

QUANTIFICATION AND STATISTICAL ANALYSIS

Two-tailed unpaired Student's t and Mann-Whitney tests, one-way or two-way analysis of variance (ANOVA) with Bonferroni's test, non-linear regression analysis, and Mantel-Cox test were performed in GraphPad Prism, with a $p < 0.05$ significance level. The Figure legends indicate the specific tests used in each graph, exact value of n and what it represents (e.g., number of animals or technical replicates), and dispersion and precision measures (e.g., mean, standard deviation, and standard error).

Cell Reports Medicine, Volume *7*

Supplemental information

A MICA/B GAALIE-mutant antibody elicits potent natural killer cell-driven immunity in solid and hematologic malignancy models

Ruan Pimenta, Stefanie Maurer, Xiaoxuan Zhong, Bruna Taciane da Silva Bortoleti, Sophia Quasem, Luiza Ribeiro de Lima Brandão, Bridget Marcellino, Anindita Dutta, Juan M. Arriaga, John Mascarenhas, and Lucas Ferrari de Andrade

Supplementary figure 1

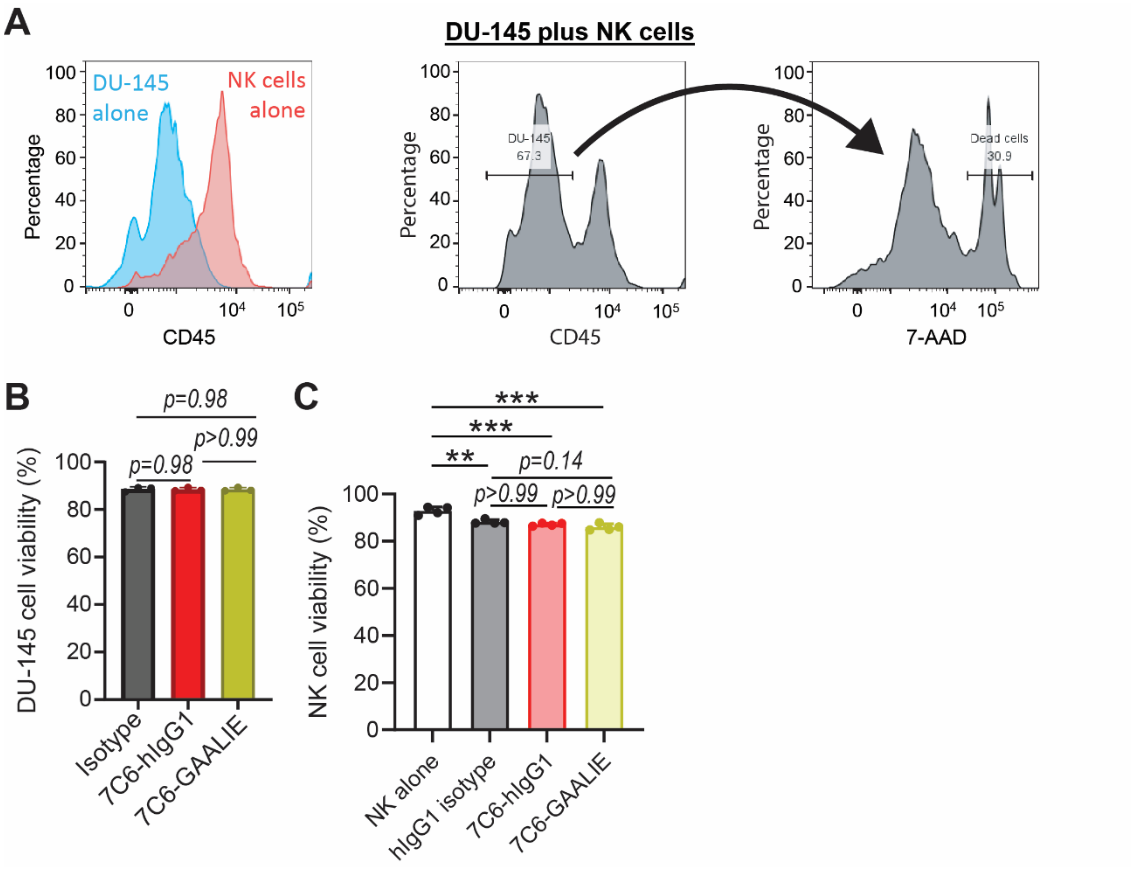

Supplementary figure 1. Representation of the flow cytometry-based NK cell cytotoxicity assay and analysis of tumor cell and NK cell viability. (A-C) DU-145 and NK cells were co-cultured for four hours, which was followed by labelling with anti-CD45 and 7-AAD. DU-145 alone and NK cells alone were used as controls. **(A)** Illustration of the gating strategy for analyzing DU-145 cell death. **(B)** Analysis of tumor cell viability in the absence of NK cells. **(C)** Analysis of NK cell viability after co-culture with tumor cells. Data are mean ± standard deviation (SD) of triplicates (B) or quadruplicates (C), represent three independent experiments with two NK cell donors (A, C), and were analyzed by one-away analysis of variance (ANOVA) with Bonferroni's test (B-C). **p<0.01, ***p<0.001. Related to Figure 1F-G.

Supplementary figure 2

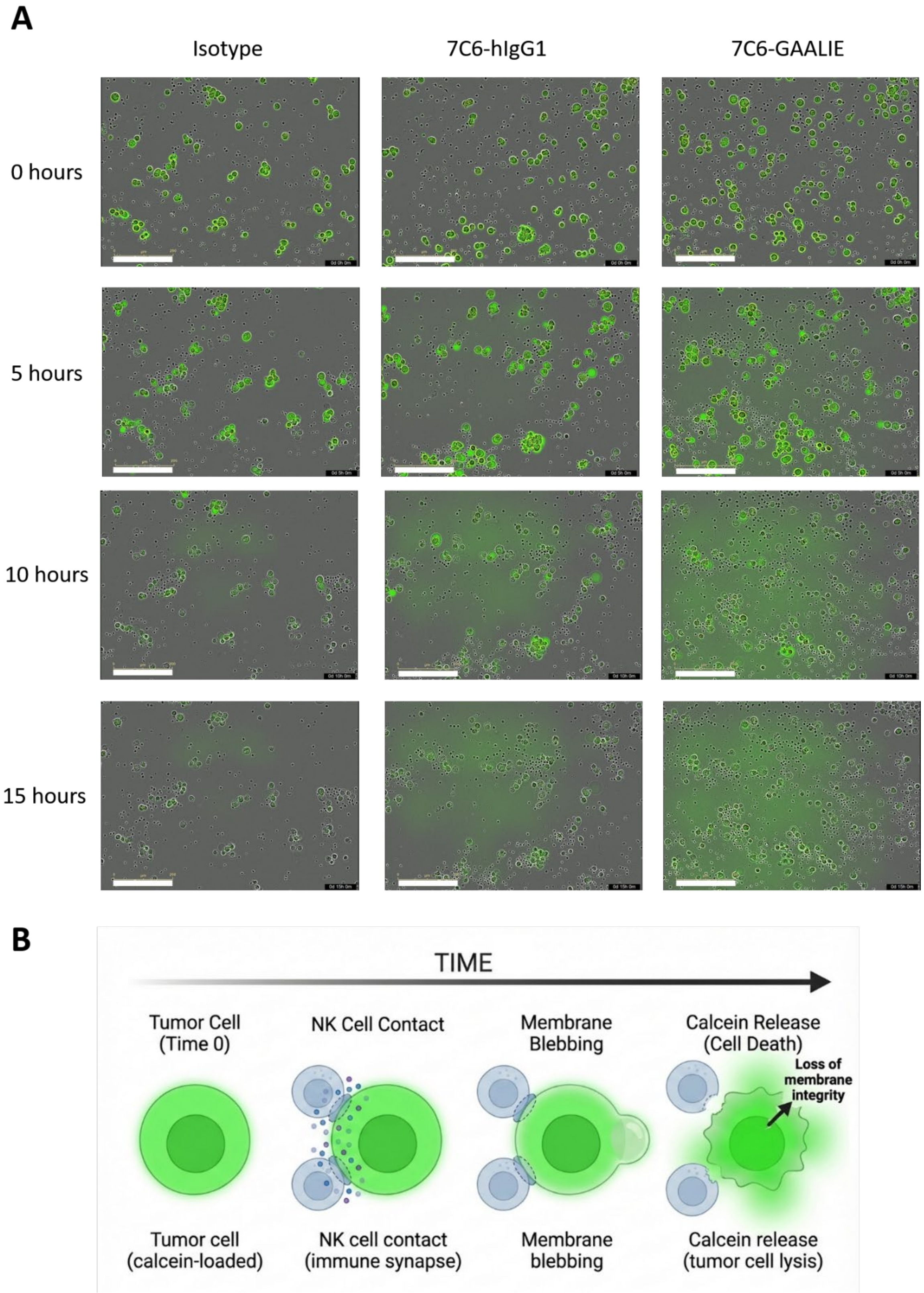

Supplementary figure 2. Analysis of human NK cell + DU-145 co-culture by IncuCyte. (A) NK cells were co-cultured with DU145 cells that were treated with the indicated antibodies and labeled with calcein (green color), and images were recorded by Incucyte at the indicated times. Data represent two independent experiments with two different NK cell donors. The scale bars represent 200 μm. **(B)** Illustration of the observed interaction between NK cells and tumor cells, whereby in the beginning of the assay all tumor cells are labeled with calcein but are then recognized and killed by NK cells, resulting in calcein release in the supernatant. Related to STAR Methods "Incucyte live-cell imaging assays."

Supplementary figure 3

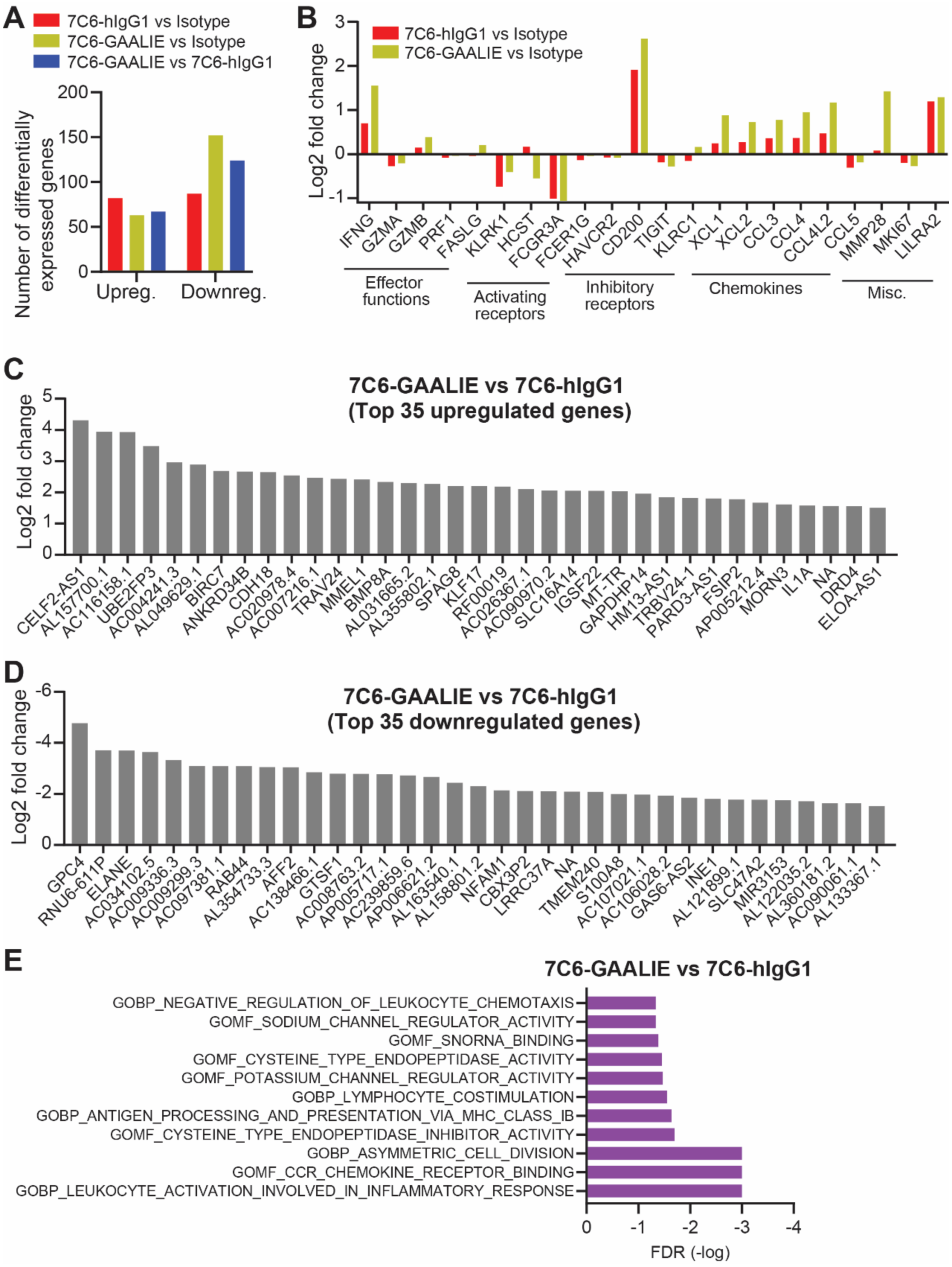

Supplementary figure 3. RNA-seq analysis of human NK cells that were co-cultured with DU-145. DU-145 cells were treated for 24-hours with hIgG1 isotype, 7C6-hIgG1, or 7C6-GAALIE, which was followed by co-culture with primary NK cells from one donor, which were then isolated and analyzed by RNA-seq. **(A)** Summary of differentially expressed genes, with 5 % significance and log2 fold change ≥ 1. Upreg. = upregulated; Downreg. = downregulated. **(B)** Gene expression analysis of some key genes related to NK cell-driven immunity. **(C - D)** The top 35 differentially expressed genes in NK cells, after the comparison of 7C6-GAALIE against 7C6-hIgG1, with 5 % significance and log2 fold change ≥ 1. **(E)** Gene set enrichment analysis of NK cells, comparing 7C6-GAALIE against 7C6-hIgG1. Related to STAR Methods “RNA-sequencing of human NK cells.”

Supplementary figure 4

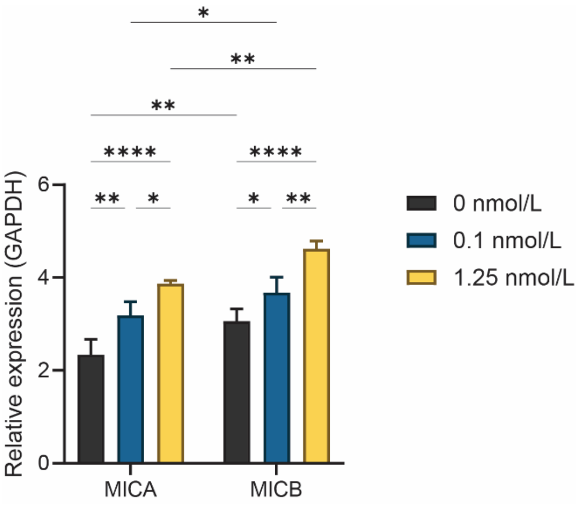

Supplementary figure 4. DU-145 cells were treated for one day with the indicated concentrations of romidepsin, and MICA/B gene expression was analyzed by quantitative polymerase chain reaction with housekeeping gene normalization. Data represent three independent experiments, are mean ± SD of triplicates, and were analyzed by two-way ANOVA with Bonferroni's test. *p<0.05, **p<0.01, ***p<0.001. Related to STAR Methods "MICA and MICB gene expression analysis."

Supplementary figure 5

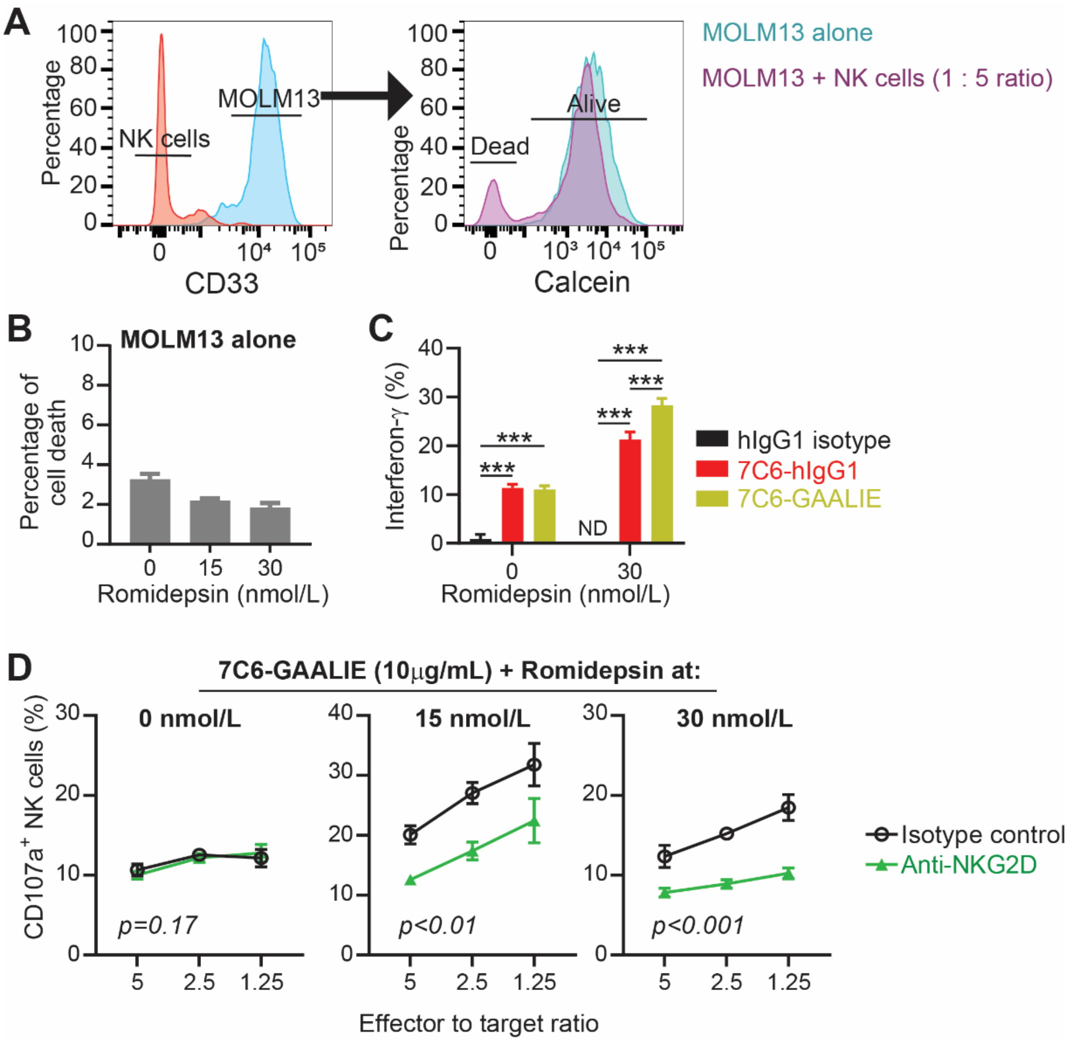

Supplementary figure 5. NK cell effector function assays with MOLM13 as the target cells. (A) Histograms of flow cytometry-based calcein assay, illustrating the identification of dead and alive MOLM13 cells after the co-culture with NK cells. **(B)** Romidepsin by itself did not kill MOLM13 cells. Quantification of dead MOLM13 cells by calcein release and flow cytometry. **(C)** 7C6-GAALIE increases the NK cell interferon-γ production. NK and MOLM13 cells were co-cultured for six hours in the presence of brefeldin A, followed by analysis of intracellular interferon-γ through flow cytometry. ND = not detected. In these experiments, MOLM13 were pre-treated with the indicated doses of romidepsin and mAbs for 24 hours. **(D)** MOLM13 was pre-treated for 24 hours with 7C6-GAALIE and the indicated doses of romidepsin. NK cells were co-cultured with the pre-treated MOLM13 cells for four hours in the presence of anti-CD107a antibody, with the addition of monensin after the first hour. Analyses by flow cytometry. Data represent three independent experiments (B-D) with three (A) or two NK cell donors (C-D), are mean ± SD (B-C) or standard error (SE) (D) of triplicates (B-D), and were analyzed by two-way ANOVA (C-D) with Bonferroni's test (C). ***p<0.001. Related to Figure 2.

Supplementary figure 6

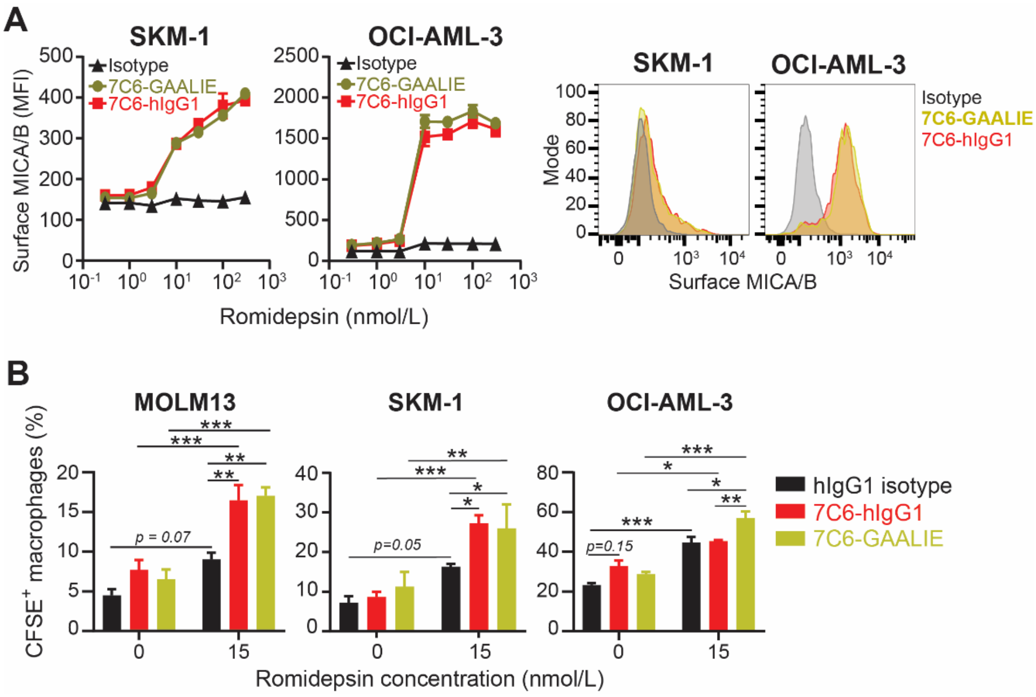

Supplementary figure 6. MICA/B surface expression upregulation in two human AML cell lines and ADCP assays. (A) SKM-1 and OCL-AML-3 cells were treated for 24 hours with the indicated antibodies and romidepsin doses, and MICA/B surface expression was analyzed by flow cytometry. The histograms represent MICA/B surface expression at romidepsin dose of 30 nmol/L. **(B)** 7C6-GAALIE and 7C6-hIgG1 trigger ADCP against romidepsin-treated MOLM13 and SKM-1, and only the first triggered ADCP against OCI-AML-3. The indicated leukemia cell lines were pre-treated for 24 hours with the indicated antibodies and romidepsin doses, labelled with CFSE, and used as target cells in 2-hours phagocytosis assays with human monocyte-derived macrophages. Analyses were by flow cytometry with macrophage identification by CD206 expression. Data represent three independent experiments (A-B) with at least two monocyte donors (B), are mean ± SD (A) or SE (B) of triplicates (A-B), and were analyzed by two-way ANOVA with Bonferroni's test (B). *p<0.05, **p<0.01, ***p<0.001. Related to Figure 2 and STAR Methods "Phagocytosis assays."

Supplementary figure 7

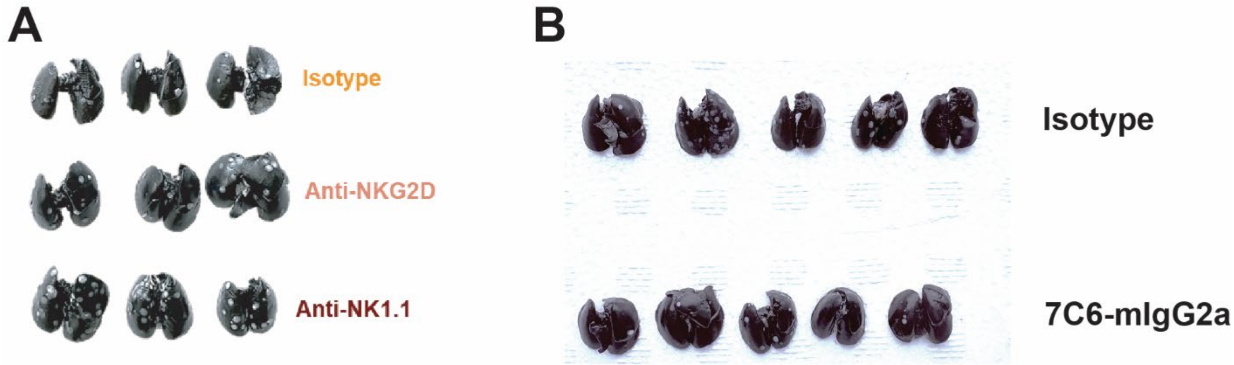

Supplementary figure 7. Representative images of lungs in the mouse prostate cancer metastasis model. (A-B) Representative images of the data that are presented in Figure 3D (A) and Figure 3G (B). Related to Figures 3D and 3G.

Supplementary figure 8

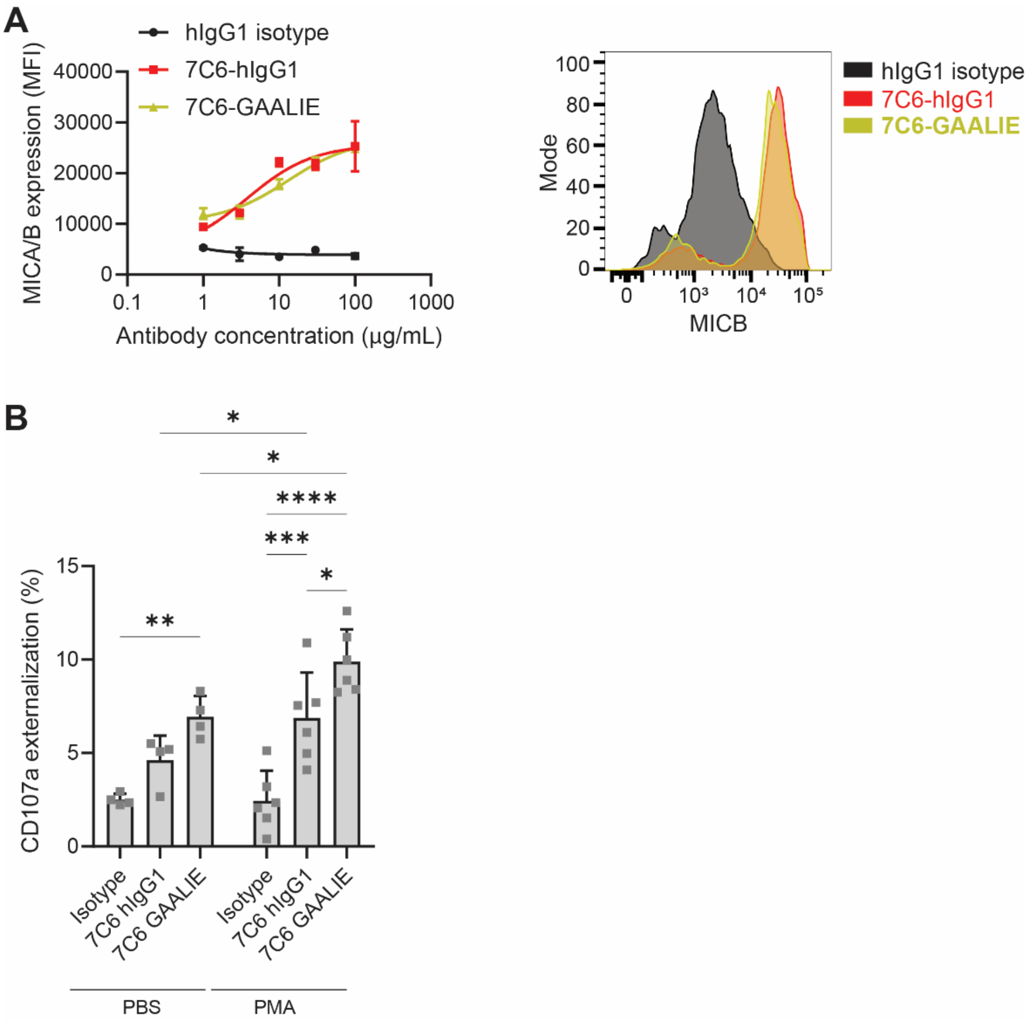

Supplementary figure 8. *In vitro* and *ex vivo* analyses of MICA/B shedding and NK cell degranulation, respectively. (A) NPK-MICB cells were treated for 24 hours with the indicated concentrations of the indicated antibodies, and MICB surface expression was analyzed by flow cytometry. The histograms represent the MICB surface expression levels at the antibody highest dose, 100 g/mL. **(B)** hFcR mice were subjected to the mouse prostate cancer metastasis model and antibody treatments as in Figure 4C. On day 14, lung NK cells were analyzed for degranulation *in vitro* after stimulation with PMA or treatment with PBS, a control. Data represent three (A) or are pooled of two (B) independent experiments, are mean ± SD (A-B), and were analyzed by non-linear regression (A) or two-way ANOVA with Bonferroni's test (B). *p<0.05, **p<0.01, ***p<0.001. Related to STAR Methods "MICA/B shedding assays with cell lines"and "Mouse prostate cancer metastasis model."

Supplementary figure 9

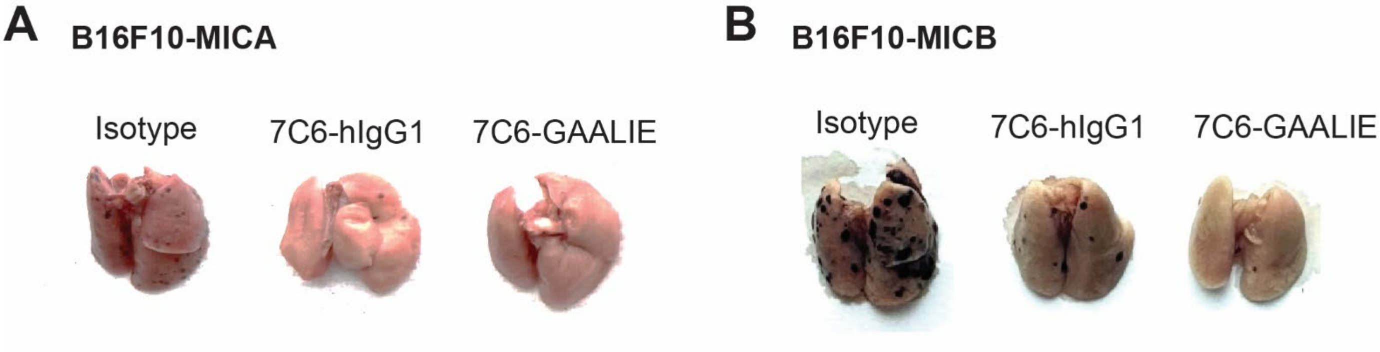

Supplementary figure 9. Representative lung images of hFcR mice that underwent the B16F10-MICA or B16F10-MICB metastasis models and treatment with antibodies. (A-B) Representative lung images of the data shown in Figure 5B. Related to Figure 5A-B.

Supplementary figure 10

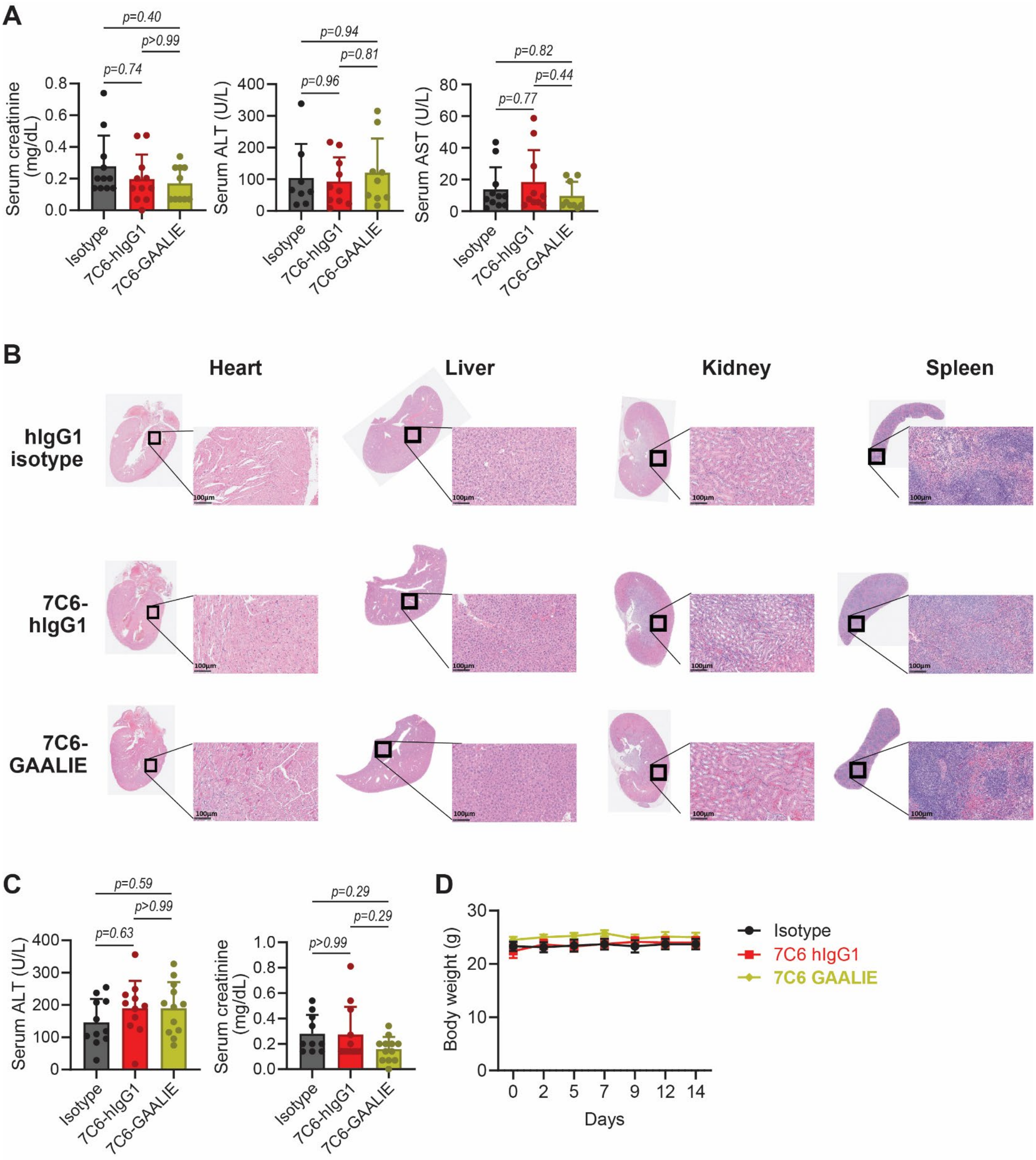

Supplementary figure 10. Toxicology of 7C6-hIgG1 and 7C6-GAALIE in mouse metastasis models. (A-B) Analysis of toxicology by serum biomarkers (A) and histopathology (B) of hFcR mice that were subjected to the mouse prostate cancer metastasis model and euthanized when reached the humane endpoint; these hFcR mice are the same of the survival experiment that is shown in Figure 4F. The scale bars represent 100 µm (B). **(C-D)** Analysis of toxicology by serum biomarkers (C) and longitudinal weight measurements (D) of hFcR mice that were subjected to the B16F10-MICA metastasis model; these analyses were performed in the same mice under the experiments that are shown in Figure 5B, B16F10-MICA. In D: Isotype hIgG1 n = 7, 7C6-hIgG1 n = 8, and 7C6-GAALIE n = 8. Data are pooled of two independent experiments (A, C-D), represent fifteen mice per antibody group (B), are mean ±

SD (A, C) or SE (D), and were analyzed by two-tailed unpaired Student's t tests with the indicated comparisons (A, C). Related to Figures 4 and 5.

Supplementary figure 11

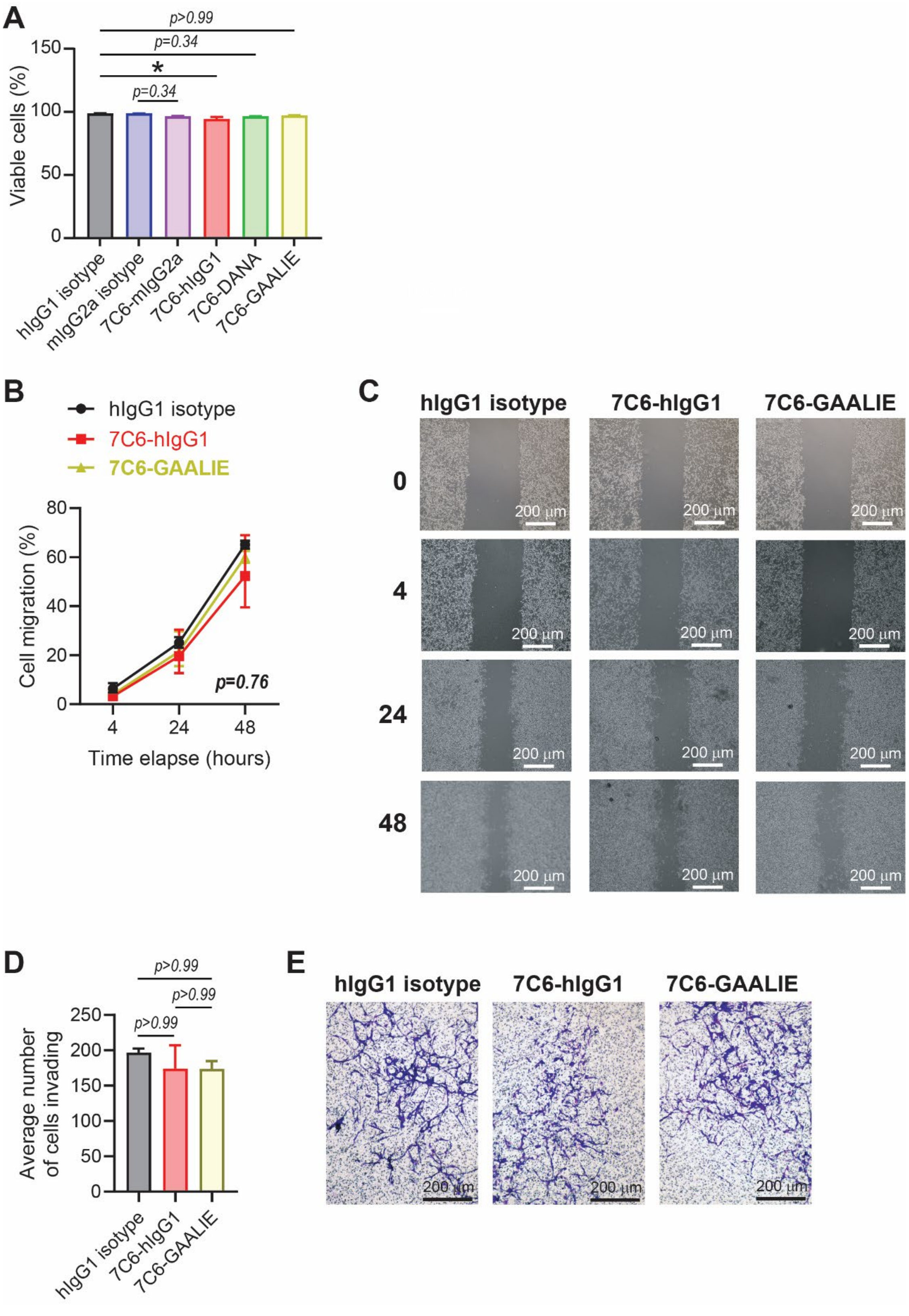

Supplementary figure 11. NPK-MICB viability, migration, and invasion analyses. (A) Analysis of NPK-MICB viability. The cells were treated for 24 hours with the indicated antibodies, and cell viability was analyzed by flow cytometry. **(B-C)** NPK-MICB migration assay. NPK-MICB cells were treated with the indicate antibodies and, at the same time, a scratch was done in the tissue culture plate, and illustrated in 'C'. Analyses of cell migration were done by microscopy on the indicated time points. **(D-E)** Analysis of NPK-MICB invasion in the transwell system. The cells were cultured for 48 hours in a transwell that was coated with matrigel, and the cells that invaded the lower surface were counted by microscopy. Data are mean ± SD (A, D) or SE (B) of duplicates (A, D) or triplicates (B), were analyzed by one-way (A, D) or two-way (B) ANOVA with Bonferroni's test (D), and represent three (A-B) or

two (C) independent experiments. *p<0.05. The scale bars represent 200 μm (C, E). Related to STAR Methods "Wound healing (scratch) assay" and "Transwell invasion assay."

Supplementary figure 12

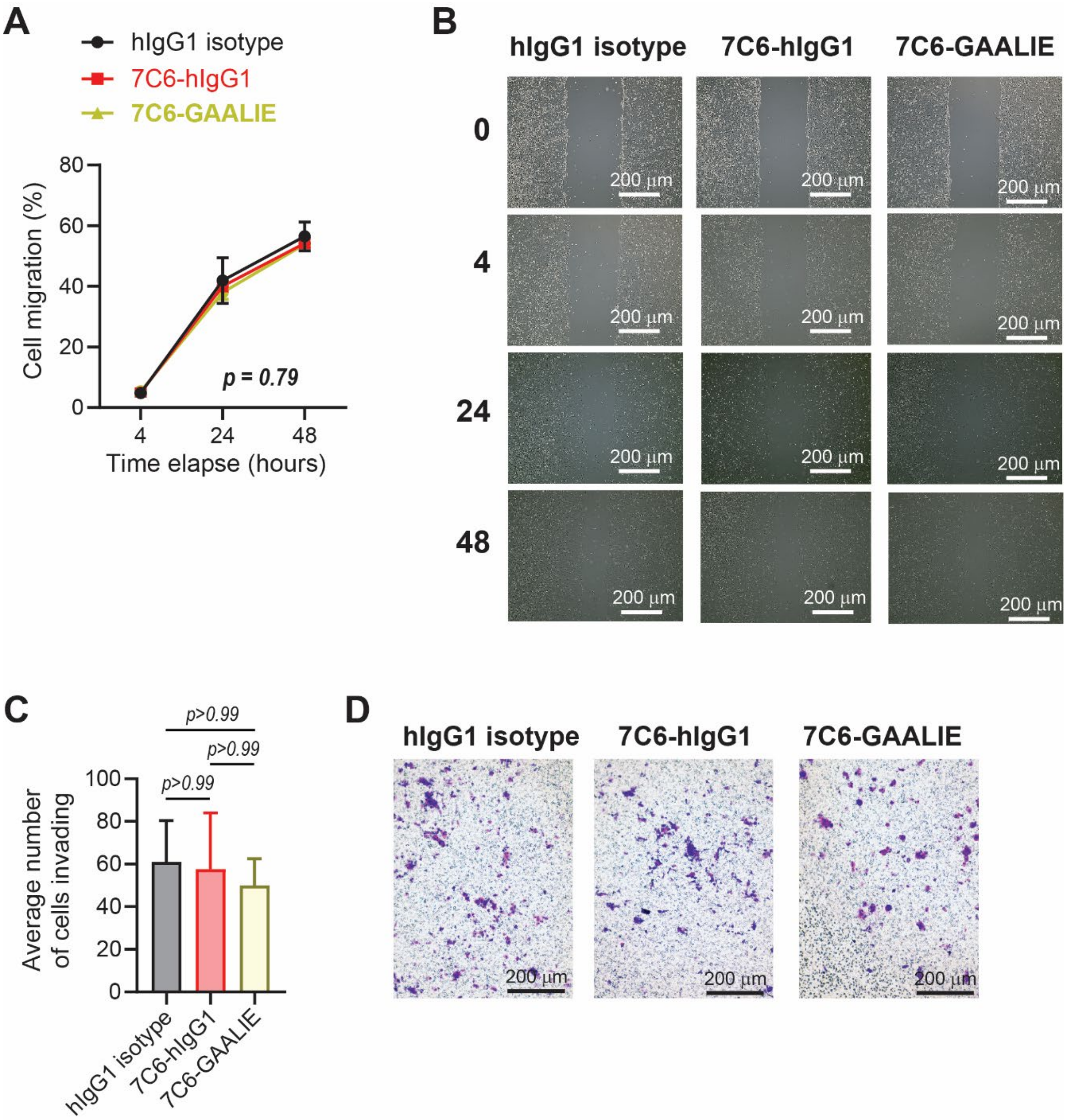

Supplementary figure 12. Migration and invasion assays with DU-145 cell line. (A-B) DU-145 cells were incubated with the indicated antibodies and a scratch was done on the tissue culture plates, as indicated in 'B'. Cell migration was analyzed by microscopy in the indicated hours. **(C-D)** DU-145 cells were cultured in transwell plates and treated with the indicated antibodies. After 48 hours, cell invasion to the lower surface was analyzed by microscopy. Data are mean ± SD (A, C) of triplicates (A, C), represent three (B) or two (D) independent experiments, and were analyzed by one-way (C) or two-way (A) ANOVA with Bonferroni's test (C). The scale bars represent 200 µm (B, D). Related to STAR Methods "Wound healing (scratch) assay" and "Transwell invasion assay."

Supplementary figure 13

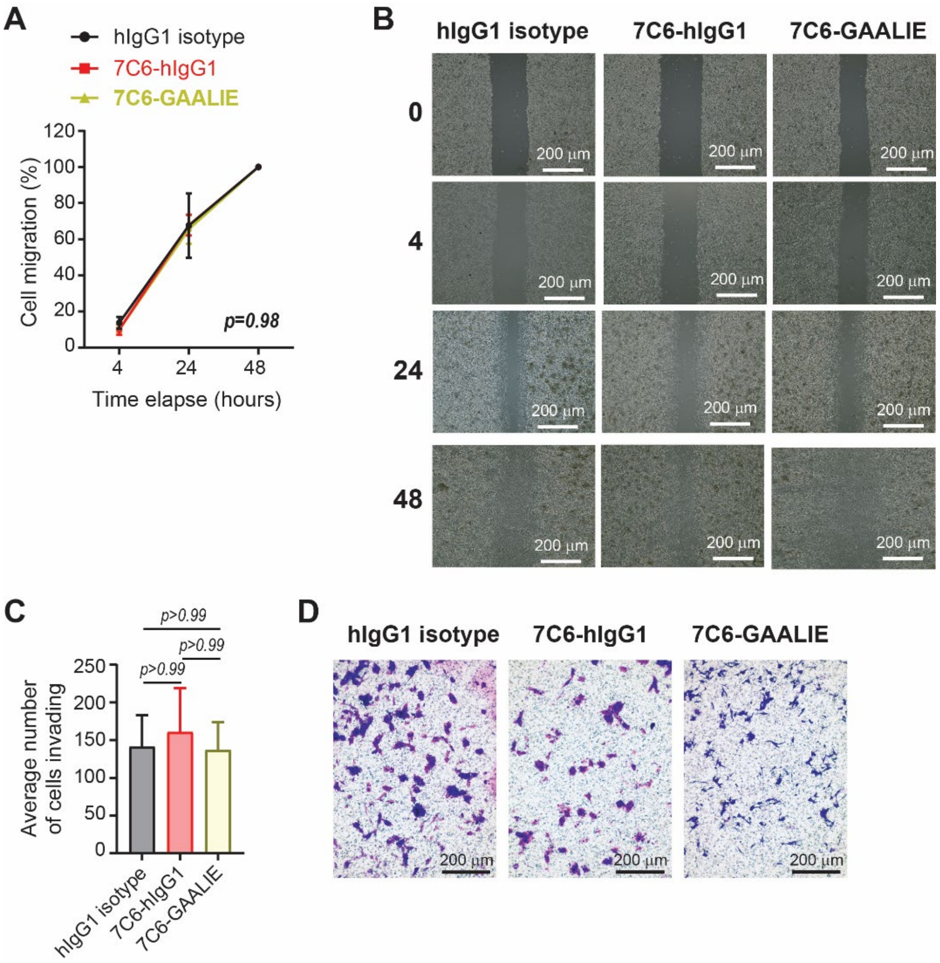

Supplementary figure 13. Migration and invasion assays with B16F10-MICA cell line. (A-B) B16F10-MICA cells were incubated with the indicated antibodies and a scratch was done on the tissue culture plates, as indicated in 'B'. Cell migration was analyzed by microscopy in the indicated hours. **(C-D)** B16F10-MICA cells were cultured in transwell plates and treated with the indicated antibodies. After 48 hours, cell invasion to the lower surface was analyzed by microscopy. Data are mean ± SD (A, C) of triplicates (A, C), represent three (A) or two (C) independent experiments, and were analyzed by one-way (C) or two-way (A) ANOVA with Bonferroni's test (C). The scale bars represent 200 μm (B, D). Related to STAR Methods "Wound healing (scratch) assay" and "Transwell invasion assay."

Supplementary figure 14

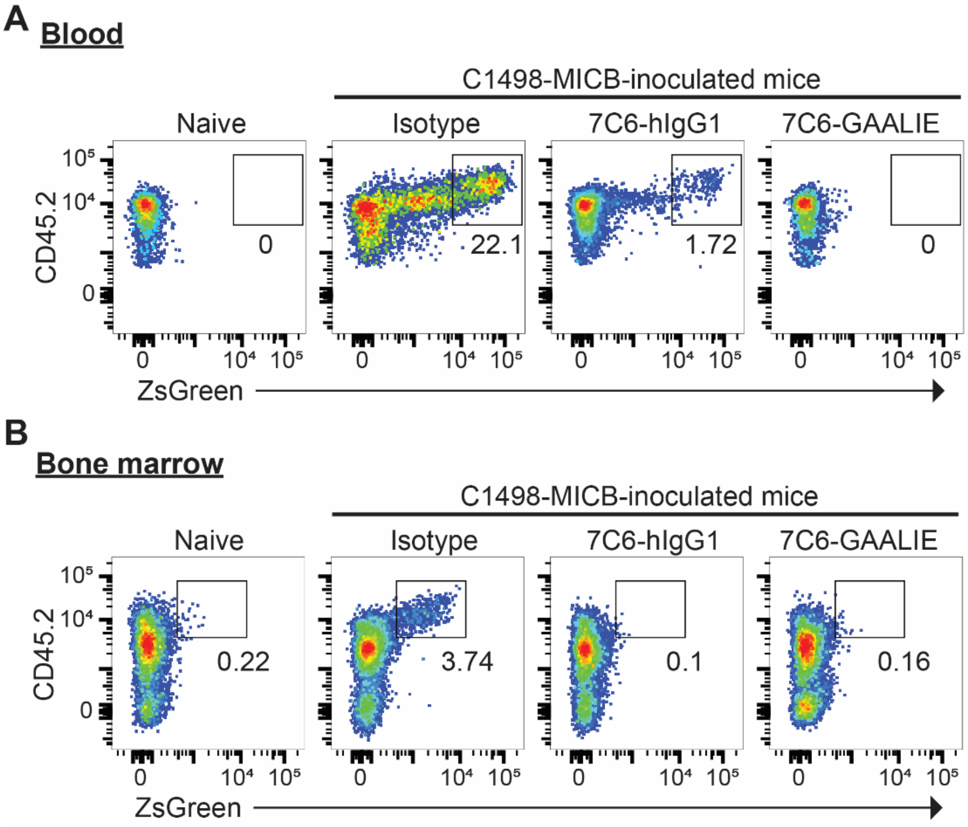

Supplementary figure 14. ZsGreen-based identification of C1498-MICB in the blood and bone marrow of hFcR mice. (A-B) Flow cytometry plots representing the data shown in Figure 5D. Related to Figure 5D.

Supplementary figure 15

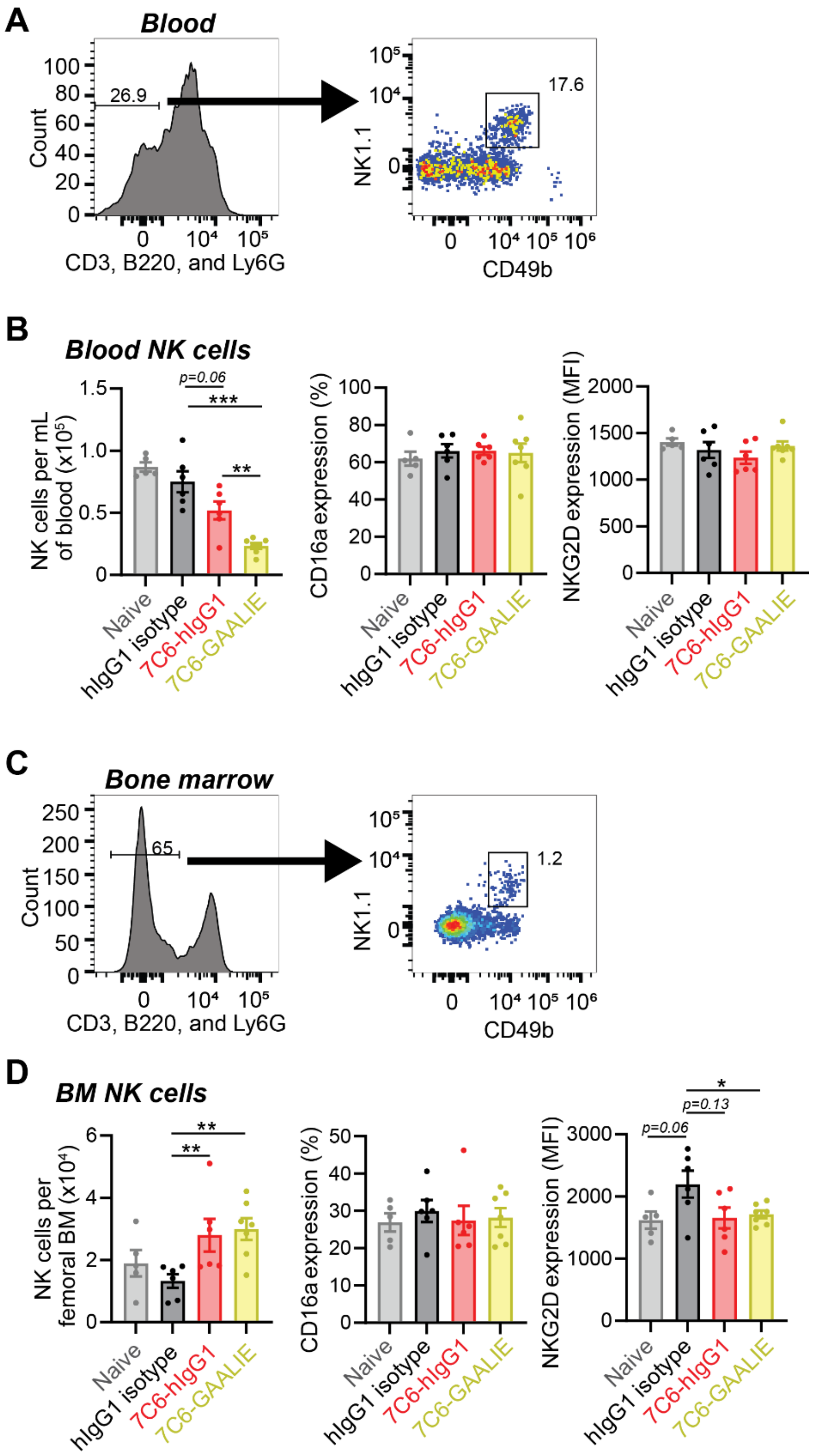

Supplementary figure 15. Characterization of NK cells in the mouse AML model in hFcR mice. (A-B) Blood NK cell analyses. **(A, C)** Gating strategies for identification of blood (A) or bone marrow (C) NK cells by flow cytometry. **(C-D)** Bone marrow (BM) NK cell analyses. Data represent two independent experiments (B, D), are mean ± SE (B, D), and were analyzed by two-tailed Mann-Whitney tests (B, D). Each dot represents one mouse (B, D). *p<0.05, **p<0.01, ***p<0.001. Related to Figure 5C-D.

Supplementary figure 16

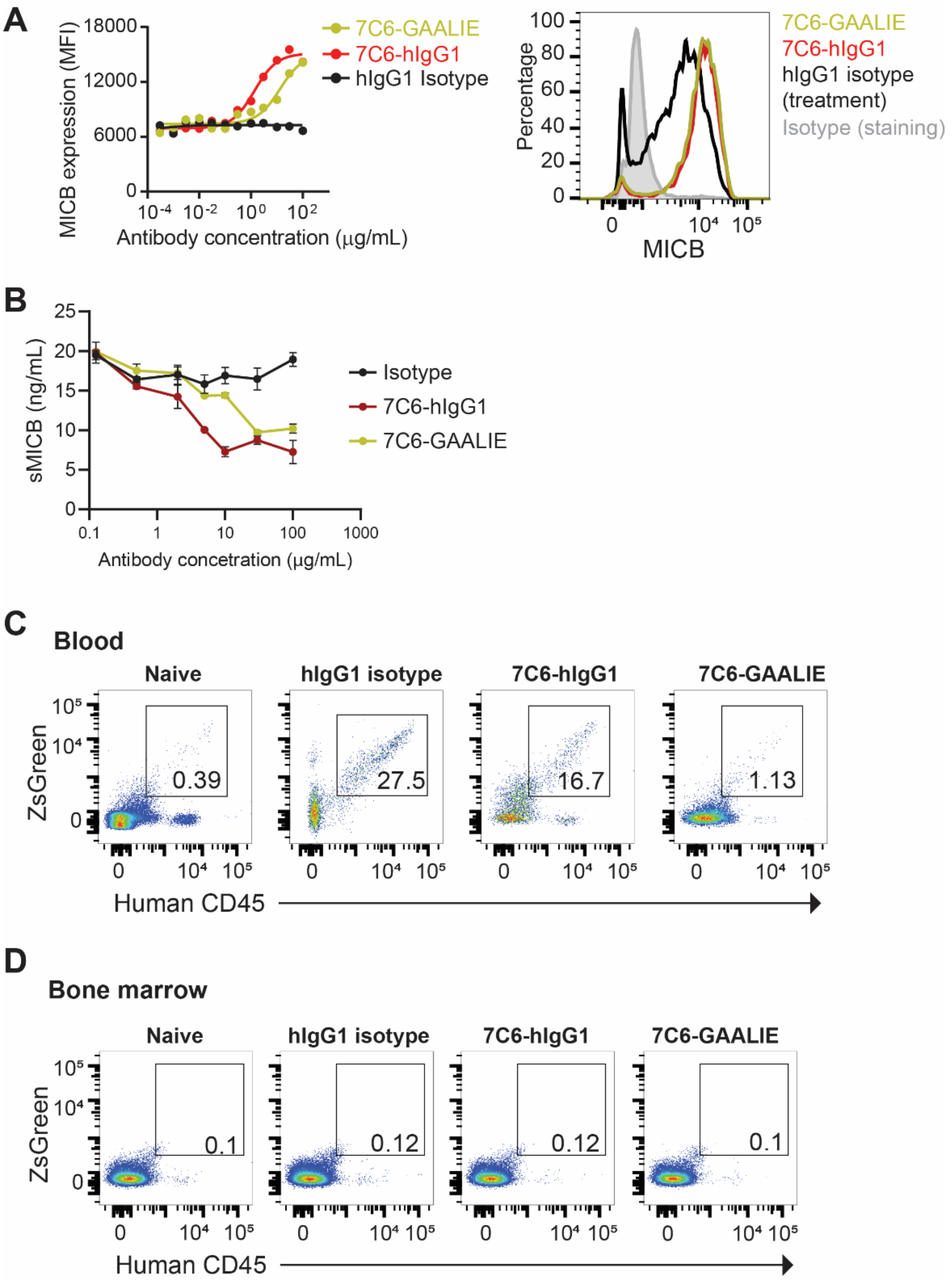

Supplementary figure 16. Validation of MOLM13-MICB and identification of MOLM13-MICB in the blood and bone marrow of FcResolv® hIL-15 NOG mice that were reconstituted with human NK cells. (A-B) MOLM13-MICB was treated for one day with the indicated concentrations of the indicated antibodies and surface MICB (A) and soluble MICB (sMICB) shed in supernatants (B) were analyzed by flow cytometry or ELISA, respectively. Data represent three independent experiments (A-B), are mean ± SD of triplicates (B) or one observation data point (A), and were analyzed by non-linear regression (A). The histograms show MICB surface expression after antibody treatment at the highest dose. **(C-D)** Flow cytometry plots representing the data of blood leukemia cells shown in Figure 5F. Mouse CD45.1^{+} cells were gated out (not shown) prior to calculation of absolute numbers. Related to Figure 5E-F.

Supplementary figure 17

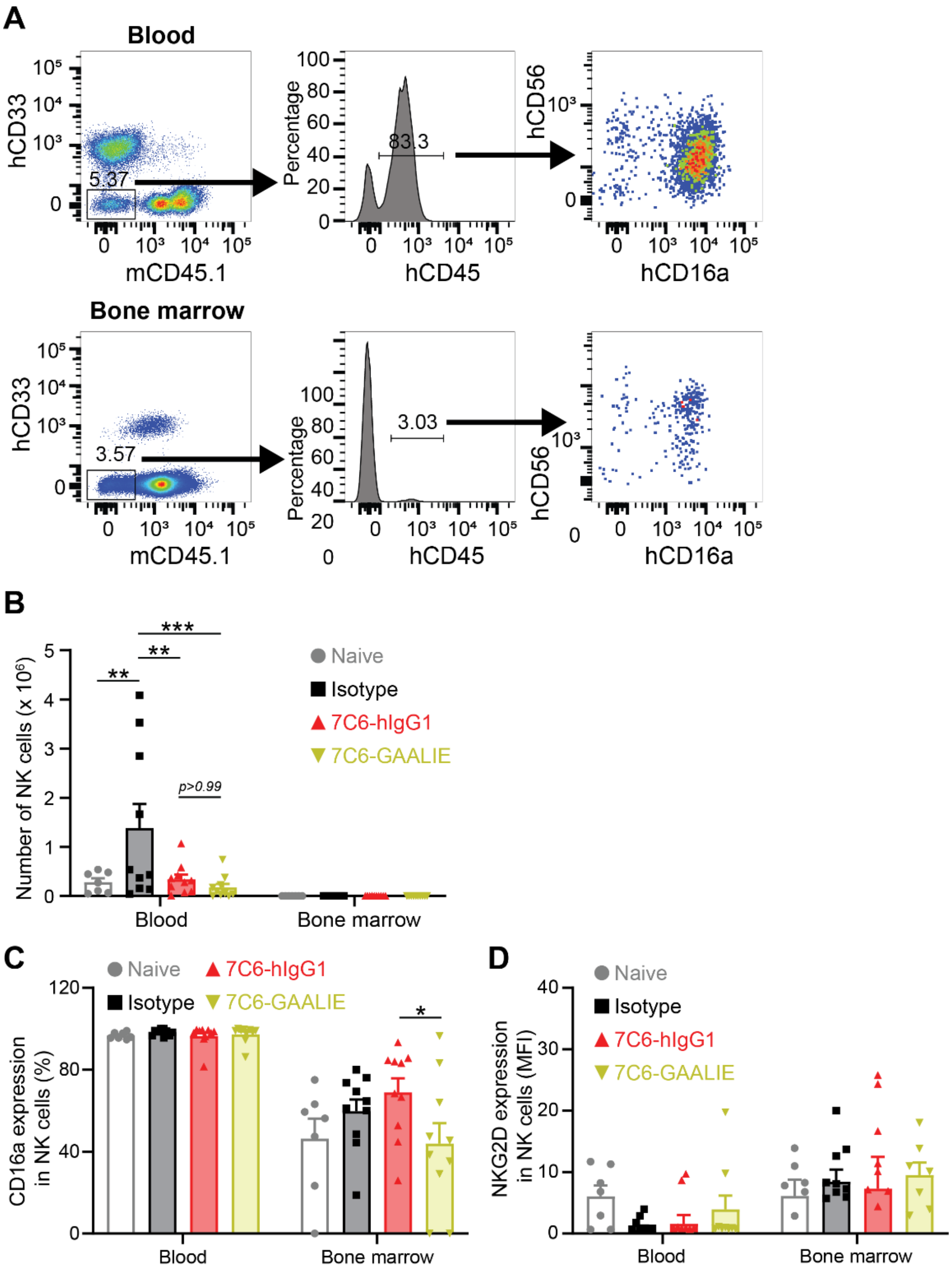

Supplementary figure 17. Characterization of human NK cells in the MOLM13-MICB model with FcResolv® hIL-15 NOG mice. (A-D) The mice were subjected to the NK cell reconstitution and MOLM13-MICB inoculation as illustrated in Figure 3E. **(A)** Flow cytometry analyses that enabled the identification of leukemia and NK cells in the blood and bone marrow. **(B)** Absolute numbers of NK cells in the blood and bone marrow. **(C)** Percentage of CD16a expression in NK cells. **(D)** NKG2D expression analyses, after normalization with the isotype control for the staining. MFI = mean fluorescence intensity. Data are mean ± SE (B-D), are pooled of two independent experiments (B-D), and were analyzed by two-way ANOVA with Bonferroni's test (B-D). Each dot represents one mouse (B-D). *p<0.05, **p<0.01, ***p<0.001. Related to Figure 5E-F.
